# Diverse Lifestyles and Strategies of Plant Pathogenesis Encoded in the Genomes of Eighteen *Dothideomycetes* Fungi

**DOI:** 10.1371/journal.ppat.1003037

**Published:** 2012-12-06

**Authors:** Robin A. Ohm, Nicolas Feau, Bernard Henrissat, Conrad L. Schoch, Benjamin A. Horwitz, Kerrie W. Barry, Bradford J. Condon, Alex C. Copeland, Braham Dhillon, Fabian Glaser, Cedar N. Hesse, Idit Kosti, Kurt LaButti, Erika A. Lindquist, Susan Lucas, Asaf A. Salamov, Rosie E. Bradshaw, Lynda Ciuffetti, Richard C. Hamelin, Gert H. J. Kema, Christopher Lawrence, James A. Scott, Joseph W. Spatafora, B. Gillian Turgeon, Pierre J. G. M. de Wit, Shaobin Zhong, Stephen B. Goodwin, Igor V. Grigoriev

**Affiliations:** 1 United States Department of Energy (DOE) Joint Genome Institute (JGI), Walnut Creek, California, United States of America; 2 Faculty of Forestry, Forest Sciences Centre, University of British Columbia, Vancouver, British Columbia, Canada; 3 Architecture et Fonction des Macromolécules Biologiques, Aix-Marseille Université, CNRS, Marseille, France; 4 NIH/NLM/NCBI, Bethesda, Maryland, United States of America; 5 Department of Biology, Technion - IIT, Haifa, Israel; 6 Department of Plant Pathology & Plant-Microbe Biology, Cornell University, Ithaca, New York, United States of America; 7 Bioinformatics Knowledge Unit, Technion - IIT, Haifa, Israel; 8 Department of Botany and Plant Pathology, Oregon State University, Corvallis, Oregon, United States of America; 9 Institute of Molecular BioSciences, Massey University, Palmerston North, New Zealand; 10 Natural Resources Canada, Ste-Foy, Quebec, Canada; 11 Plant Research International, Wageningen, The Netherlands; 12 Virginia Bioinformatics Institute & Department of Biological Sciences, Blacksburg, Virginia, United States of America; 13 Division of Occupational & Environmental Health, Dalla Lana School of Public Health, University of Toronto, Toronto, Canada; 14 Laboratory of Phytopathology, Wageningen University, Wageningen, The Netherlands; 15 Department of Plant Pathology, North Dakota State University, Fargo, North Dakota, United States of America; 16 United States Department of Agriculture, Agricultural Research Service, Purdue University, West Lafayette, Indiana, United States of America; University of Melbourne, Australia

## Abstract

The class *Dothideomycetes* is one of the largest groups of fungi with a high level of ecological diversity including many plant pathogens infecting a broad range of hosts. Here, we compare genome features of 18 members of this class, including 6 necrotrophs, 9 (hemi)biotrophs and 3 saprotrophs, to analyze genome structure, evolution, and the diverse strategies of pathogenesis. The *Dothideomycetes* most likely evolved from a common ancestor more than 280 million years ago. The 18 genome sequences differ dramatically in size due to variation in repetitive content, but show much less variation in number of (core) genes. Gene order appears to have been rearranged mostly within chromosomal boundaries by multiple inversions, in extant genomes frequently demarcated by adjacent simple repeats. Several *Dothideomycetes* contain one or more gene-poor, transposable element (TE)-rich putatively dispensable chromosomes of unknown function. The 18 *Dothideomycetes* offer an extensive catalogue of genes involved in cellulose degradation, proteolysis, secondary metabolism, and cysteine-rich small secreted proteins. Ancestors of the two major orders of plant pathogens in the *Dothideomycetes*, the *Capnodiales* and *Pleosporales*, may have had different modes of pathogenesis, with the former having fewer of these genes than the latter. Many of these genes are enriched in proximity to transposable elements, suggesting faster evolution because of the effects of repeat induced point (RIP) mutations. A syntenic block of genes, including oxidoreductases, is conserved in most *Dothideomycetes* and upregulated during infection in *L. maculans*, suggesting a possible function in response to oxidative stress.

## Author Summary

*Dothideomycetes* is the largest and most ecologically diverse class of fungi that includes many plant pathogens with high economic impact. Currently 18 genome sequences of *Dothideomycetes* are available, 14 of which are newly described in this paper and in several companion papers, allowing unprecedented resolution in comparative analyses. These 18 organisms have diverse lifestyles and strategies of plant pathogenesis. Three feed on dead organic matter only, six are necrotrophs (killing the host plant cells), one is a biotroph (forming an association with and thus feeding on the living cells of the host plant cells) and 8 are hemibiotrophs (having an initial biotrophic stage, and killing the host plant at a later stage). These various lifestyles are also reflected in the gene sets present in each group. For example, sets of genes involved in carbohydrate degradation and secondary metabolism are expanded in necrotrophs. Many genes involved in pathogenesis are located near repetitive sequences, which are believed to speed up their evolution. Blocks of genes with conserved gene order were identified. In addition to this we deduce that the mechanism for mesosynteny, a type of genome evolution particular to *Dothideomycetes*, is by intra-chromosomal inversions.

## Introduction

*Dothideomycetes* is the largest and most ecologically diverse class of fungi [1]. One or more members of this class infect almost every major crop, including those involved in the production of food, feed, fiber and biofuel. In addition to housing important plant pathogens, the class includes fungi with an unparalleled diversity of life history strategies and metabolic profiles. *Dothideomycetes* are present on every continent, including Antarctica, and are very important to ecosystem health and global carbon cycling as saprotrophs and degraders of plant biomass. Many are tolerant of environmental extremes including heat, cold, solar radiation and desiccation. Some produce enzymes that help degrade rocks [2] while others are associated with alcoholic vapors [3]. A few are pathogens of humans or livestock, and two of the species that are ubiquitous colonizers of dead plant biomass affect human health as well because they are important allergens known to exacerbate asthma [4]. Adaptations to fresh- or salt-water aquatic habitats have occurred multiple times within *Dothideomycetes* [5], [6]. Other *Dothideomycetes* are lichenized and grow on exposed surfaces of rocks, plants or manmade structures [7]. Some are associated with plants asymptomatically as endophytes or epiphytes. In addition, a single lineage exists in a symbiotic relationship with plant roots as ectomycorrhizae with a broad host and geographic range [8].

Dothideomycete taxonomy has been strongly influenced by classifications based on the development and morphology of the sexual structures (e.g., bitunicate asci or meiosporangia). However, the advent of DNA sequence comparisons indicated that species with these typical traits reside in two classes, *Dothideomycetes* and *Eurotiomycetes* (e.g., *Aspergillus* and relatives). *Dothideomycetes* share a most recent common ancestor with another class, *Arthoniomycetes*, a small group of mainly lichenized and lichenicolous fungi [9], [10]. Recent phylogenetic analyses also indicate that *Dothideomycetes*, *Arthoniomycetes* and *Eurotiomycetes* form a larger clade with a fourth diverse class of mainly lichenized fungi, *Lecanoromycetes*, but their interclass relationships remain poorly resolved and await additional evidence from genome-scale analyses. Importantly, the resolution of these relationships is necessary to further resolve the evolution of fungal ecologies (e.g., lichens, endophytes, etc. [11])

Current taxonomy of the *Dothideomycetes* divides the class into 12 orders containing more than 1,300 genera and 19,000 species [12], [13]. The majority of lineages in the class remains unsampled with DNA sequence data and resists cultivation. For example, there are recent DNA-based hints at diversity consistent with several additional orders [7], [14], [15]. Within the currently defined orders ecological diversity remains high. Although all members of one order, the *Jahnulales*, are aquatic from fresh water or very damp habitats [16] and the *Trypetheliales* contains lichenized species [7], members of the remaining orders are mostly terrestrial saprotrophs, with diverse lifestyles that have independently evolved multiple times [17].

Plant pathogens occur in at least six of the 12 orders. The two largest dothideomycete orders, *Pleosporales* and *Capnodiales*, each contain a large number of highly destructive plant pathogens. These include some of the most important diseases of the cereal crops wheat, barley and maize, trees such as pine and poplar, dicots including soybeans, canola and tomato, and tropical fruits including bananas. The allergens of *Davidiella tassiana* (aka *Cladosporium herbarum*) and *Alternaria alternata* are the most important of all known fungal allergens and represent two of the four allergens associated with these two orders.

Their high economic impact and intriguing biological diversity have stimulated much interest in genomic sequencing of *Dothideomycetes*. Key representatives have been sequenced through the Fungal Genome program at the U.S. Department of Energy Joint Genome Institute (JGI), which has had an emphasis on *Dothideomycetes* for several years [18]. The sequenced species are important to agriculture, especially those that are pathogens of bioenergy crops, or they represent phylogenetic and ecological diversity like AFTOL (Assembling the Fungal Tree of Life) targets [19], or they are of interest to bioenergy production because of their unusual physiology such as *Baudoinia compniacensis*, whose growth on outdoor surfaces is induced by fugitive ethanol vapor emissions from spirit maturation warehouses and bakeries [20]. These extensive efforts have yielded more sequences of fungi in the *Dothideomycetes* than any other class, providing an unparalleled opportunity for comparative genomics.

Here we report new genome sequences of 14 dothideomycete genomes and use them in comparative analyses with those of four *Dothideomycetes* published previously [21], [22], [23], [24], plus representative outgroups from the *Ascomycota* and *Basidiomycota* (Table 1), a total of 39 genomes. Among the 18 sequenced dothideomycete genomes, nine are from species of the order *Pleosporales*, seven from the order *Capnodiales* and two from the order *Hysteriales*. From the perspective of lifestyle, fifteen are from species of plant pathogens (six necrotrophs, eight hemibiotrophs and one biotroph) and three are saprotrophs.

**Table 1:** Species used in this comparative study.

Species	Taxonomy		Lifestyle	Host/substrate	Sequencing Center	Ref
	Class	Order				
***Dothideomycetes***						
*Cochliobolus heterostrophus C5*	*Dothideomycetes*	*Pleosporales*	Necrotrophic plant pathogen	Maize (*Zea mays*)	JGI (USA)	(unpublished data)^a^
*Cochliobolus heterostrophus C4*	*Dothideomycetes*	*Pleosporales*	Necrotrophic plant pathogen	Maize (*Zea mays*)	JGI (USA)	(unpublished data)^a^
*Cochliobolus sativus*	*Dothideomycetes*	*Pleosporales*	Hemibiotrophic plant pathogen	Wheat, barley and other grasses	JGI (USA)	(unpublished data)^a^
*Setosphaeria turcica*	*Dothideomycetes*	*Pleosporales*	Hemibiotrophic plant pathogen	Maize (*Zea mays*)	JGI (USA)	(unpublished data)^a^
*Alternaria brassicicola*	*Dothideomycetes*	*Pleosporales*	Necrotrophic plant pathogen	*Brassica* spp.	Washington State University (USA)	[78]
*Pyrenophora teres* forma *teres*	*Dothideomycetes*	*Pleosporales*	Necrotrophic plant pathogen	Barley (*Hordeum vulgare*)	Curtin University, (Australia)	[22]
*Pyrenophora tritici-repentis*	*Dothideomycetes*	*Pleosporales*	Necrotrophic plant pathogen	Wheat (*Triticum* spp.)	BROAD (USA)	[44]
*Leptosphaeria maculans*	*Dothideomycetes*	*Pleosporales*	Hemibiotrophic plant pathogen	*Brassica* spp.	INRA/Genoscope (France)	[23]
*Stagonospora nodorum*	*Dothideomycetes*	*Pleosporales*	Necrotrophic plant pathogen	Wheat (*Triticum* spp.)	BROAD (USA)	[21]
*Hysterium pulicare*	*Dothideomycetes*	*Hysteriales*	Saprotrophic	Tree bark and wood	Oregon State University (USA)	
*Rhytidhysteron rufulum*	*Dothideomycetes*	*Hysteriales*	Saprotrophic	Various including plants	Oregon State University (USA)	
*Mycosphaerella populicola*	*Dothideomycetes*	*Capnodiales*	(Hemi)biotrophic plant pathogen	Poplar trees (*Populus* spp.)	University of British Columbia (Canada)	(unpublished data)^a^
*Mycosphaerella populorum*	*Dothideomycetes*	*Capnodiales*	(Hemi)biotrophic plant pathogen	Poplar trees (*Populus* spp.)	JGI (USA)	(unpublished data)^a^
*Mycosphaerella fijiensis*	Dothideomycetes	Capnodiales	Hemibiotrophic plant pathogen	Banana (*Musa* spp.)	JGI (USA)	(unpublished data)^a^
*Cladosporium fulvum*	*Dothideomycetes*	*Capnodiales*	Biotrophic plant pathogen	Tomato (*Solanum lycopersicum*)	Wageningen University & Research centre (The Netherlands)	[73]
*Dothistroma septosporum*	*Dothideomycetes*	*Capnodiales*	Hemibiotrophic plant pathogen	Pines and other conifers	JGI (USA)	[73]
*Mycosphaerella graminicola*	*Dothideomycetes*	*Capnodiales*	Hemibiotrophic plant pathogen	Wheat (*Triticum* spp.)	JGI (USA)	[24]
*Baudoinia compniacensis*	*Dothideomycetes*	*Capnodiales*	Saprotrophic, extremophile	Ethanol vapor. Hard surfaces	JGI (USA)	(unpublished data)^a^
**Outgroup Ascomycota**						
*Aspergillus nidulans*	*Eurotiomycetes*	*Eurotiales*	Saprotrophic	Soil	AspGD consortium	[119]
*Botrytis cinerea*	*Leotiomycetes*	*Helotiales*	Necrotrophic plant pathogen	Fruit and leaves of many species	BROAD	[120]
*Chaetomium globosum*	*Sordariomycetes*	*Sordariales*	Saprotrophic	Dead plant material	BROAD	[121]
*Fusarium graminearum*	*Sordariomycetes*	*Hypocreales*	Necrotrophic plant pathogen	Wheat (*Triticum* spp.) and barley (*Hordeum vulgare*)	BROAD	[122]
*Fusarium oxysporum*	*Sordariomycetes*	*Hypocreales*	Hemibiotrophic plant pathogen/endophyte/saprotrophic	Many plant hosts; soil	BROAD	[123]
*Magnaporthe grisea*	*Sordariomycetes*	*Magnaporthales*	Hemibiotrophic plant pathogen	Rice (*Oryza sativa*)	BROAD	[124]
*Nectria haematococca*	*Sordariomycetes*	*Hypocreales*	Necrotrophic plant pathogen/saprotrophic	Many plant hosts; soil	JGI	[125]
*Neurospora crassa*	*Sordariomycetes*	*Sordariales*	Saprotrophic	Dead plant material, particularly after fires	BROAD	[126]
*Saccharomyces cerevisiae*	*Saccharomycetes*	*Saccharomycetales*	Saprotrophic	Fruit	SGD consortium	[127]
*Sclerotinia sclerotiorum*	*Leotiomycetes*	*Helotiales*	Necrotrophic plant pathogen	Many, primarily dicots	BROAD	[120]
*Trichoderma reesei*	*Sordariomycetes*	*Hypocreales*	Saprotrophic	Soil, woody surfaces	JGI	[128]
*Verticillium dahliae*	*Sordariomycetes*	*Hypocreales*	Necrotrophic plant pathogen	Many dicots	BROAD	[129]
**Outgroup Basidiomycota**						
*Coprinopsis cinerea*	*Agaricomycetes*	*Agaricales*	Saprotrophic	Dung, plant material	BROAD	[130]
*Cryptococcus neoformans* var. *grubii*	*Tremellomycetes*	*Tremellales*	Animal pathogen	Humans	BROAD	[131]
*Laccaria bicolor*	*Agaricomycetes*	*Agaricales*	Ectomycorrhizal	Many tree species	JGI	[35]
*Melampsora laricis-populina*	*Urediniomycetes*	*Uredinales*	Obligate biotrophic plant pathogen	Poplar trees (*Populus* spp.)	JGI	[132]
*Phanerochaete chrysosporium*	*Agaricomycetes*	*Polyporales*	Saprotrophic	Wood	JGI	[133]
*Postia placenta*	*Agaricomycetes*	*Polyporales*	Saprotrophic	Wood	JGI	[134]
*Puccinia graminis*	*Urediniomycetes*	*Uredinales*	Obligate biotrophic plant pathogen	Wheat (*Triticum* spp.)	BROAD	[132]
*Schizophyllum commune*	*Agaricomycetes*	*Agaricales*	Saprotrophic	Dead wood	JGI	[135]
*Ustilago maydis*	*Ustilaginomycetes*	*Ustilaginales*	Biotrophic plant pathogen	Maize (*Zea mays*) and teosinte (*Euchlena mexicana*)	BROAD	[136]

a Additional genome-centric papers are planned.

Taxonomy and lifestyle of the 18 *Dothideomycetes* used in this study, as well as the outgroups for comparative purposes.

The order *Pleosporales* comprises the necrotroph *Cochliobolus heterostrophus* (isolates C4 and C5) and the hemibiotroph *Setosphaeria turcica* which infect corn (*Zea mays*), the hemibiotroph *Cochliobolus sativus* which infects barley, wheat and several other cereal crops, the necrotrophs *Pyrenophora tritici-repentis* and *Stagonospora nodorum* which infect wheat (*Triticum aestivum*), the necrotroph *Pyrenophora teres* forma *teres* which infects barley (*Hordeum vulgare*), and the necrotroph *Alternaria brassicicola* and the hemibiotroph *Leptosphaeria maculans* which infect plants in the *Brassicaceae*.

The order *Capnodiales* comprises the hemibiotroph *Mycosphaerella graminicola* (*Zymoseptoria tritici*) which infects wheat, the hemibiotroph *Dothistroma septosporum* (*Mycosphaerella pini*) which infects more than 70 species of pine, the hemibiotrophs *Mycosphaerella populorum* (*Septoria musiva*) and *Mycosphaerella populicola* (*Septoria populicola*) which infect species of poplar, the hemibiotroph *Mycosphaerella fijiensis* which infects bananas (*Musa* spp.), the biotroph *Cladosporium fulvum* (*Passalora fulva*) which infects tomato (*Solanum lycopersicum*), and the extremophilic saprotroph *Baudoinia compniacensis*. The latter's primary known habitat is various exposed substrates near liquor maturation warehouses and commercial bakeries [20], where ambient ethanol vapors provoke its colonization.

The order *Hysteriales* comprises the two saprotrophs *Hysterium pulicare* and *Rhytidhysteron rufulum*. Phylogenetically these species form a sister group to the plant pathogens in the *Pleosporales* [25], and are usually associated with dead or dying plant tissues.

Comparative genomic analysis of 18 *Dothideomycetes* provides valuable insights into fundamental questions regarding fungal lifestyles, evolution and adaptation to diverse ecological niches, especially as they relate to plant pathogenicity and biomass conversion.

## Results/Discussion

### *Dothideomycetes* phylogeny and divergence time estimates

**Figure 1:**
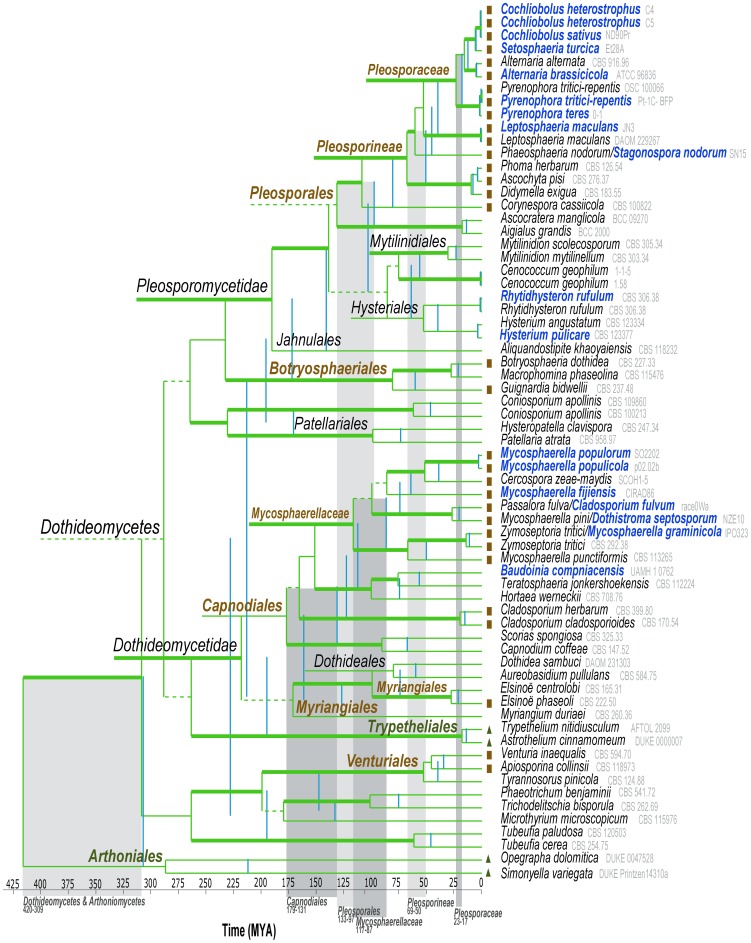
Estimated phylogeny and divergence times of *Dothideomycetes*, based on sequences of three protein-coding genes. Species with a sequenced genome that are included in this study are highlighted in dark blue. Vertical lines in blue and green indicate minimum and maximum ages for specific nodes, respectively. The age ranges for highlighted taxa are indicated by blocks with different shades of gray. Horizontal green lines indicate bootstrap recovery for specific nodes – thickened branches represent more than 70%, normal branches, 50–70% and less than 50% are indicated with dashed lines. In some cases relevant horizontal lines were stylistically extended to highlight node labels. Only families with multiple genomes are indicated. Orders, suborders and families that contain important plant-pathogenic species are colored brown and those containing majority lichenized species are green. Brown squares indicate plant pathogenic and green triangles lichenized species. Saprotrophs and fungi with other nutritional modes are not labeled.

**Figure 2:**
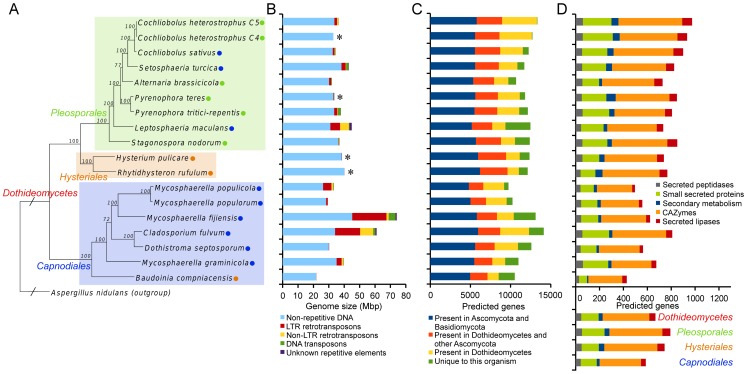
Phylogeny and genome characteristics of the 18 studied *Dothideomycetes*. **A.** Genome-based phylogenetic tree of 18 *Dothideomycetes* computed using 51 conserved protein families. Bootstrap values are indicated on the branches. Lifestyles and strategies of pathogenesis (green circle for necrotrophs, orange circle for saprotrophs and blue circle for [hemi]biotrophs) are indicated. *Aspergillus nidulans* was used as an outgroup and its branch on the tree is not drawn to scale. **B.** Genome size and repeat content. Repeat content varies widely among *Dothideomycetes*, but in general the largest part consists of long terminal repeats. Asterisks indicate genomes that were sequenced exclusively with Illumina technology. Repeat content in these genomes is likely an underestimate. **C.** Number of predicted genes, broken down by level of conservation. **D.** Gene counts of classes that have been implicated in plant pathogenesis. Members of *Capnodiales* have fewer genes in these classes than *Pleosporales* and *Hysteriales* (with the exception of *Cladosporium fulvum*). This trend is also illustrated by the estimated gene counts for the last common ancestors of the indicated taxa (below the x-axis), which correspond to the taxa in (A). See also Figure S3. Bars on all graphs (B, C, and D) correspond to the organisms on the tree in (A).

The class *Dothideomycetes* comprises a huge diversity of fungi. To place the sequenced species in a broader evolutionary context, a three-gene phylogenetic tree was made representing 11 of 12 currently accepted orders in *Dothideomycetes* (Figure 1). This 67-taxon phylogeny is congruent with a tree made from 51 orthologous genes obtained from the 18 genome-sampled strains (Figure 2A) and previous phylogenies [17]. Divergence time estimates are indicated and age ranges of the taxonomic groups relevant for this paper are indicated in differently shaded gray blocks in Figure 1.

The class of *Dothideomycetes* last shared a common ancestor more than 280 million years ago (MYA). Genome sampling in this class is currently focused on two large and diverse orders, *Pleosporales* and *Capnodiales*, and to a lesser extent on *Hysteriales*. The main radiation of *Capnodiales* likely happened between 179 and 131 MYA, while a similar event likely occurred for the *Pleosporales* at a later date, between 133 and 97 MYA. This latter estimation is very likely influenced by our limited sampling of early-diverging lineages in *Pleosporales*. However, differences in divergence times become more pronounced in the two highlighted families with more representative sampling. *Mycosphaerellaceae*, as defined currently, represents an ancient (diversifying at least 87 MYA) clade compared to *Pleosporaceae* (diversifying at least 17 MYA). The sampled *Hysteriales* shared a common ancestor at least 40 MYA. Figure 1 also illustrates that the strains currently labeled with the genus name *Mycosphaerella* diversified across a longer time than all species in *Pleosporaceae* and its several sister lineages. These lineages are included in suborder *Pleosporineae* which represents a well recovered phylogenetic node containing the four main families of plant pathogens in *Pleosporales*.

Additional considerations concerning phylogeny and nomenclature of *Dothideomycetes* are discussed in Text S1.

### Variation in genome sizes across diverse *Dothideomycetes*

Genome sizes show dramatic variation among the *Dothideomycetes* (Figure 2B, Table S1), from 21.88 Mbp in *Baudoinia compniacensis* to 74.14 Mbp in *Mycosphaerella fijiensis*. The correlation between genome size and repeat content (0.91) is larger than the correlation between genome size and gene count (0.59) or between genome size and gene content (0.71), suggesting that the repeat content generally plays the largest role in determining genome size. When the genomes that have been sequenced exclusively using Illumina technology are excluded (see Table S1), the correlation between genome size and repeat content is even higher (0.94). Repeat content in Illumina-sequenced genomes is likely to be underestimated, since short repetitive reads are difficult to assemble into long repeat regions (as discussed in [26]). This underestimation is most apparent when *C. heterostrophus* strain C5 (Sanger assembly, 8.64% repeat content) is compared to strain C4 (Illumina assembly, 0.83% repeat content). To better estimate the repetitive content of Illumina-sequenced genomes an additional analysis was performed using the unassembled sequence reads and the assembler ALLPATHS-LG [26]. This analysis estimates the percentage of sequence reads that are repetitive. These percentages are considerably higher than those in Figure 2B (20% for *P. teres* f. *teres*, 9% for *H. pulicare*, 18% for *R. rufulum*, and 20% for *C. heterostrophus* C4), but it should be noted that they were obtained using fundamentally different methods, making direct comparisons difficult. It is clear, however, that repeat content is underestimated in Illumina assemblies (and possibly also in Sanger/454 assemblies).

The smallest of the 18 genomes is that of the extremophile *B. compniacensis*, which has four features consistent with its size compared to the other *Dothideomycetes*: lower repeat content; lower number of genes; fewer genes with an intron; and shorter intergenic space (Table S1). The largest genomes, those of *M. fijiensis* and *C. fulvum*, contain 39.5% and 44.4% repeats, respectively, which are among the largest fractions reported in fungi.

### From macro- to mesosynteny

The range of evolutionary distances among the members of this group of organisms offers a unique perspective on evolution of genome organization. It has been shown previously that filamentous *Ascomycota* and particularly *Dothideomycetes* display a phenomenon recently designated as mesosynteny [27]. Mesosynteny is characterized by conservation within chromosomes of gene content but not gene order or orientation, and this was demonstrated by whole-genome DNA comparisons. In organisms displaying mesosynteny most chromosomal rearrangements are intra- rather than inter-chromosomal.

**Figure 3:**
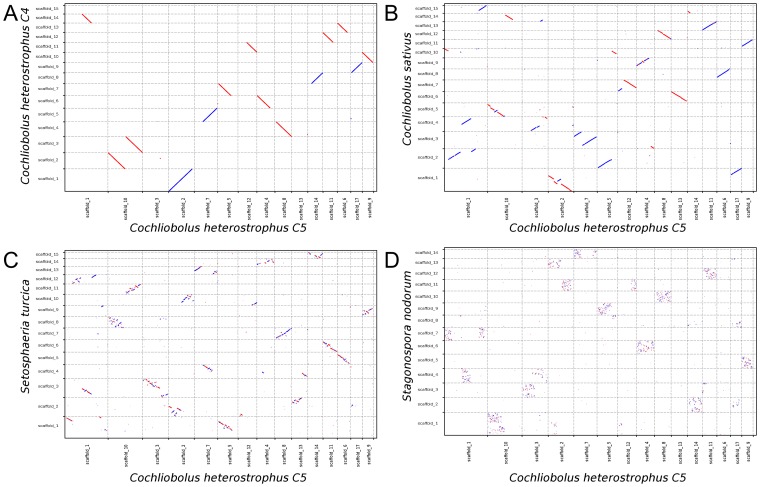
Whole-genome DNA comparison of *Cochliobolus heterostrophus* C5 to progressively distantly related organisms reveals the process leading from macrosynteny to mesosynteny. **A.** Strains C4 and C5 of *C. heterostrophus* are progeny of *C. heterostrophus* backcrosses and show clear macrosynteny. **B.** When *C. heterostrophus* C5 is compared to *C. sativus*, macrosynteny is observed. However, intra-chromosomal inversions are observed in several comparisons of scaffold pairs. **C.** Numerous intra-chromosomal inversions have occurred in all scaffolds when compared to *Setosphaeria turcica*. **D.** A pattern of mesosynteny is observed when compared to *Stagonospora nodorum*. Syntenic regions are short and spread across the scaffold pairs. Scaffolds in this figure are not drawn to scale and only a subset of the scaffolds is depicted.

When synteny analysis is extended from four [27] to 18 dothideomycete genomes, a range of syntenic relationships between organisms becomes apparent, from macro- to mesosynteny (Figure 3 and Table S2). Mesosynteny is found in the majority of genome-genome comparisons between species of *Dothideomycetes*. In contrast, macrosynteny is observed only in pairwise comparisons of the most closely related organisms: the three *Cochliobolus* genomes and between *M. populicola* and *M. populorum*. Nearly perfect macrosynteny is observed when strains C4 and C5 of *C. heterostrophus* are compared (Figure 3A), reflecting their close relationship as progeny of a backcross series. Mostly macrosyntenic conservation also is seen when either of the *C. heterostrophus* strains is compared to *C. sativus* (last common ancestor estimated less than 1 MYA, Figure 1). Interestingly, however, large intra-chromosomal inversions have taken place in several sequence pairs (Figure 3B). The same phenomenon is observed in a comparison between *M. populicola* and *M. populorum*. The signature of macrosynteny is less clear and the pattern of mesosynteny becomes stronger in a comparison between *C. heterostrophus* C5 and *S. turcica* (Figure 3C, last common ancestor estimated 5–6 MYA). Finally, when *C. heterostrophus* C5 is compared to the more distantly related *S. nodorum* a pattern of mesosynteny is observed (Figure 3D, last common ancestor estimated 45–61 MYA) that is very similar to that observed between other pairs of *Dothideomycetes* [27]. We hypothesize that the intra-chromosomal inversions observed between the genomes of *C. heterostrophus* and *C. sativus* are the first steps in the development of the mesosyntenic patterns observed between more distantly related *Dothideomycetes*.

**Figure 4:**
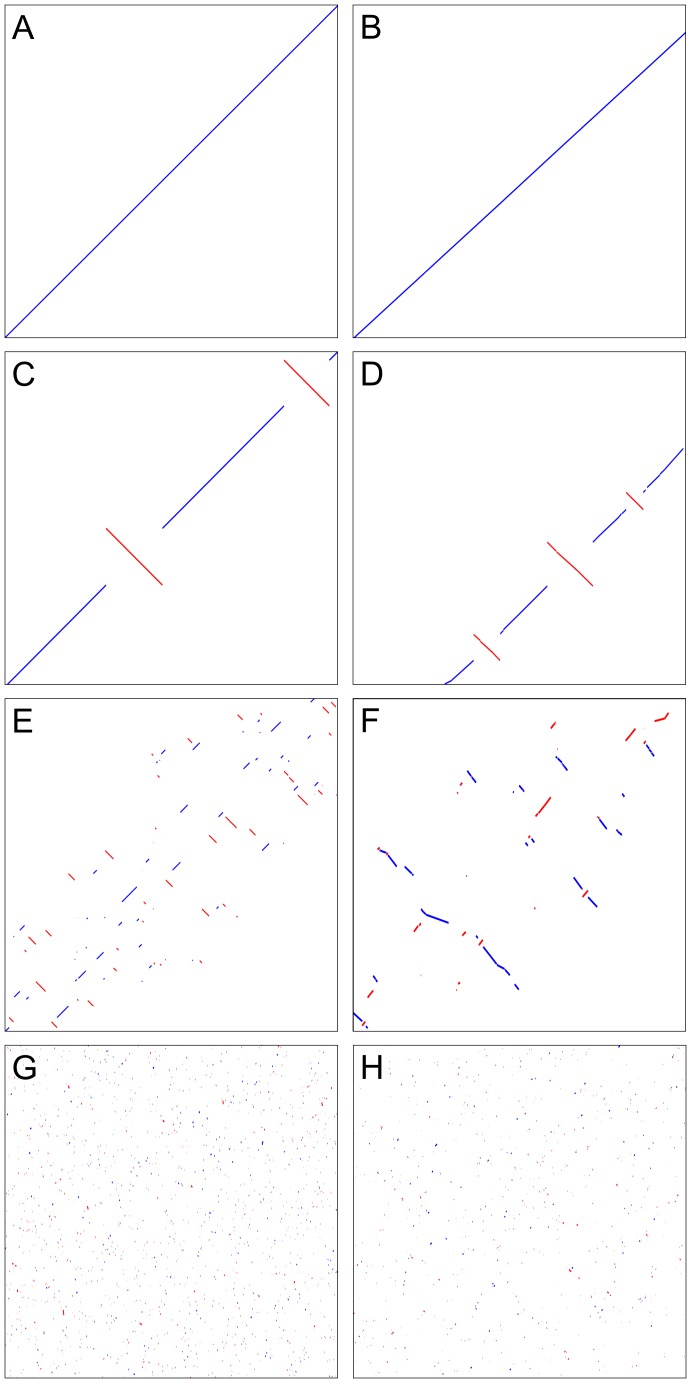
Simulation of chromosome evolution leading to mesosynteny. **A.** Two identical sequences show perfect macrosynteny. **B.** This is also the case for scaffold_1 of *Cochliobolus heterostrophus* C4 and scaffold_2 of *C. heterostrophus* C5, reflecting their close relationship as progeny. **C.** The two sequences from (A) have each undergone one random inversion. **D.** Scaffold_4 of *C. heterostrophus* C5 and scaffold_9 of *C. sativus* show a very similar pattern as in (C). **E.** The two sequences in (A) have each undergone 25 random inversions. **F.** Scaffold_8 of *Setosphaeria turcica* and part of scaffold_10 of *C. heterostrophus* C5 show a pattern of syntenic regions progressively spreading across the scaffolds similar to that in (E) **G.** The two sequences from (A) have each undergone 500 random inversions. Syntenic regions are short and spread homogeneously across the two scaffolds. **H.** Scaffold_1 of *Dothistroma septosporum* and scaffold_1 of *Mycosphaerella populorum* show a very similar pattern as in (G). Scaffolds in this figure are not drawn to scale.

To test whether inversions could generate the observed patterns of mesosynteny, we ran a simulation of the evolution of a diverging pair of chromosomes undergoing intra-chromosomal inversions. Initially, the chromosome pairs are identical and therefore fully macrosyntenic (Figure 4A), similar to the pattern observed between *C. heterostrophus* strains C4 and C5 (Figure 4B). After one random intra-chromosomal inversion in each chromosome (Figure 4C), the pattern is very similar to that observed between *C. heterostrophus* C5 and *C. sativus* (Figure 4D). After 25 random inversions, syntenic regions are progressively spreading across the scaffolds (Figure 4E), similar to what is observed for *C. heterostrophus* C5 and *S. turcica* (Figure 4F). After 500 random inversions (Figure 4G), the pattern is very similar to that observed between *D. septosporum* and *M. populorum* (Figure 4H), which diverged from the same ancestor an estimated 74–100 MYA (Figure 1). This simulation shows that intra-chromosomal inversions alone are sufficient to obtain a pattern of mesosynteny between two genomes during evolution.

In most genomes all the chromosomes/scaffolds show mesosynteny with at least one chromosome/scaffold from another *Dothideomycete*. The exceptions are *L. maculans* (lm_SuperContig_22_v2), *M. graminicola* (chr_16 and chr_19), and *M. fijiensis* (scaffold_11, scaffold_15, scaffold_17, scaffold_18, and scaffold_20). Interestingly, all these scaffolds/chromosomes are (predicted to be) dispensable (see section ‘Putatively dispensable chromosomes’, below).

Interestingly, the inversion breakpoints are associated with simple repeats (i.e., low-complexity DNA such as dinucleotide repeats). Among relatively closely related mesosyntenic scaffold pairs, these simple repeats are over-represented in the 500 bp up- and downstream of these breakpoints, compared to the rest of the respective scaffolds (comparisons 1, 2 and 3 in Table S3). In more distantly related scaffold pairs this pattern is not observed (comparison 4 in Table S3), presumably because ancient inversion sites have since changed considerably.

Although the exact mechanism leading to mesosynteny is still unknown, this extended analysis using 18 genomes of *Dothideomycetes* was consistent with the simulations and added sufficient resolution to be able to show that frequent intra-chromosomal inversions most likely played a major role in the origin of this phenomenon. Whether the frequency and placement of simple repeats is different in the *Dothideomycetes* than organisms that do not show patterns of mesosynteny is not known.

### Microsynteny is conserved across large groups of *Dothideomycetes*

Chromosomal rearrangement events (such as those leading to mesosynteny) will theoretically result eventually in a random distribution of genes across chromosomes, except for certain clusters of genes associated with a common function for which physical clustering is beneficial (e.g., secondary metabolism). Although physical clustering of functionally related fungal genes does occur, it is considerably more rare than in prokaryotes. The physical clustering of genes across related organisms can therefore give insight into functional relationships between genes.

In the genomes of the 18 *Dothideomycetes* two blocks of genes were identified that were conserved in 15 and 14 of the 18 studied strains. Both blocks consist of at least 5 genes that are located in a block of at most 10 genes (Tables S4 and S5). Block 1 consists of genes with annotations that do not seem obviously related from a functional point of view. In contrast, block 2 contains genes encoding two dehydrogenases and two (oxido)reductases, which strongly suggests a functional connection. These two blocks were not present in any of the outgroups used in this study.

Interestingly, in *L. maculans* 3 of 6 genes in block 1 are at least 2-fold down-regulated and all 5 genes in block 2 are at least 2-fold up-regulated in leaves 7 or 14 days after infection, when compared to expression in mycelium (reanalyzed expression data obtained from previously published whole-genome microarray data [23]; see also ‘comparative transcriptomics during pathogenesis’ in Text S3, Tables S6 and S7). This apparent co-regulation in *L. maculans* may be an effect of the physical clustering on the chromosome, but it also suggests a related functional role where co-location may provide a fitness advantage. Because the genes in block 2 in *L. maculans* were up-regulated in infected leaves, they could play a role in pathogenesis in that organism. Since they are conserved in nearly all sequenced genomes of *Dothideomycetes*, these blocks may have been present in the common ancestor of all *Dothideomycetes* and were maintained throughout their evolutionary history.

The same microsynteny analysis was performed on two *Dothideomycetes* subsets: the *Pleosporales* (excluding *C. heterostrophus* strain C4, since it is very similar to strain C5) and the *Mycosphaerellaceae* (see Figure 1). This resulted in 502 and 58 syntenic blocks of genes present in at least 75% of the studied organisms in each group, respectively (Tables S8 and S9). This difference can be explained (at least in part) by the much shorter evolutionary distances among the 8 examined *Pleosporales* (last common ancestor estimated 41–61 MYA, Figure 1), compared to those among the 6 studied *Mycosphaerellaceae* (last common ancestor estimated 87–117 MYA). An analysis of functional annotation terms of the genes in syntenic blocks reveals enrichment of genes involved in a wide variety of biological processes in the *Pleosporales* (Table S10). In the *Mycosphaerellaceae*, however, genes enriched in conserved blocks are mostly involved in transcriptional regulation (Table S11).

It was shown previously that the MAT1 mating type loci of several *Pleosporales* show conservation of gene order [28]. In our microsynteny analysis the MAT1 locus corresponds to the adjacent syntenic blocks 141 and 142 (Table S8). In addition to the 3 genes described previously, our study reveals that at least 10 genes have been conserved in location across at least 6 *Dothideomycetes*.

Despite the progressive reshuffling of the chromosomes by the processes behind mesosynteny, many syntenic blocks of genes have remained intact. For closely related species, this can be explained by the short evolutionary time in which chromosomal rearrangements could occur. The syntenic gene blocks identified across *Dothideomycetes*, however, most likely were selected for during evolution. Molecular manipulation of the genes in these syntenic blocks should help reveal function and possible reasons for the conservation of their gene order.

### Putatively dispensable chromosomes

The sequenced strain of *M. graminicola* has been shown previously to contain 8 dispensable chromosomes [24]. One or more of these chromosomes could be missing in progeny of sexual crosses and in field isolates. However, isolates missing one or more of these dispensable chromosomes show no obvious phenotypic changes compared to their parents or other progeny isolates [29]. Similarly dispensable chromosomes (in the literature also referred to as supernumerary chromosomes, B chromosomes or minichromosomes [30]) have been identified previously in the *Dothideomycetes L. maculans* [31], *C. heterostrophus* [32] and *A. alternata* [33], as well as in several other filamentous fungi (reviewed in [30]).

Compared to the core chromosomes, the dispensable chromosomes of *M. graminicola* are generally smaller, have a lower GC content, have higher repeat content, are less gene dense, and the percentage of predicted proteins with a PFAM domain is lower (Table 2). Using these criteria, we screened the other *Dothideomycetes* for chromosomes or scaffolds that are potentially dispensable. Only scaffolds larger than 100 kbp were taken into account. Scaffolds containing long rDNA repeats (as determined by RNAmmer [34]) were removed from the dataset as they probably represent unplaced contigs.

**Table 2:** Potential core and dispensable chromosomes in the genomes of *Dothideomycetes*.

Organism	Category	Size excluding gaps (bp)	GC-content (%)	Gene count	Gene density (genes/Mbp)	Gene products with PFAM domain (%)	Repeat content (%)	Name
*Mycosphaerella graminicola*	Core chromosomes	35077646	52.33	10317	294.12	50.01	11.32	
	Dispensable chromosomes	4602608	50.65	654	142.09	3.36	19.42	chr_14, chr_15, chr_16, chr_17, chr_18, chr_19, chr_20, chr_21
*Mycosphaerella fijiensis*	Putative core scaffolds	63951705	45.85	12699	198.57	50.76	37.12	
	Putative dispensable scaffolds	9779308	40.71	408	41.72	2.45	56.72	scaffold_11, scaffold_13, scaffold_14, scaffold_15, scaffold_16, scaffold_17, scaffold_18, scaffold_20, scaffold_21, scaffold_22, scaffold_23, scaffold_24, scaffold_25, scaffold_26
*Leptosphaeria maculans*	Putative core scaffolds	42453795	45.55	12423	292.62	45.79	31.59	
	Putative dispensable scaffolds	919483	35.42	42	45.68	9.52	89.36	lm_SuperContig_22_v2, lm_SuperContig_29_v2
*Setosphaeria turcica*	Putative core scaffolds	38177662	51.46	11695	306.33	55.94	14.46	
	Putative dispensable scaffolds	74746	41.92	7	93.65	14.29	72.32	scaffold_28
*Stagonospora nodorum*	Putative core scaffolds	36907047	50.52	12380	335.44	51.61	2.77	
	Putative dispensable scaffolds	142552	28.14	0	0.00	0.00	32.97	scaffold_46
*Cochliobolus heterostrophus C5*	Putative core scaffolds	36190278	49.81	13322	368.11	53.51	8.39	
	Putative dispensable scaffolds	134153	40.04	14	104.36	7.14	85.38	scaffold_27

*Mycosphaerella graminicola* has been shown previously to contain dispensable (i.e., not necessary for survival) chromosomes [24]. These chromosomes are smaller, less gene-dense and more repeat-rich than the core chromosomes. Proteins encoded by genes on these chromosomes less frequently contain a PFAM domain. Scaffolds with similar characteristics are also present in five other *Dothideomycetes*. Additional statistics for these scaffolds are given in Table S12.

Genome scaffolds with the above mentioned characteristics of the *M. graminicola* dispensome were identified in five other *Dothideomycetes*: 14 in *M. fijiensis*, 2 in *L. maculans*, and 1 each in *C. heterostrophus* C5, *S. turcica* and *S. nodorum* (Table 2, Table S12). *L. maculans* has been shown previously by pulsed-field gel electrophoresis to contain at least one dispensable chromosome of 650 to 950 kbp [31]. It was identified previously as supercontig 22 (730 kbp) [23], but it may also include supercontig 29 (200 kbp), since this supercontig shows very similar characteristics to supercontig 22 (Table S12). To our knowledge, no dispensable chromosomes have been identified previously in *S. nodorum* or *S. turcica*. In contrast to the *M. graminicola* sequence, none of these genomes is finished so it is possible that these potentially dispensable scaffolds are in fact part of larger core chromosomes, and additional dispensable chromosomes with other characteristics may also exist. Segregation patterns in progeny of a cross could determine whether these scaffolds indeed are dispensable.

The origin and evolutionary benefit of dispensable chromosomes is unknown, although horizontal transfer from other fungi has been suggested as a possible origin [24]. The observation that chromosomal rearrangements take place mostly within chromosomes (see above) causes these dispensable chromosomes to remain isolated and have a separate evolutionary history from the core chromosomes, regardless of whether they have a function.

### Gene content comparison across phylogeny and lifestyle

Predicted gene complements within the *Dothideomycetes* range from 9,739 in *M. populicola* to 13,336 in *C. heterostrophus* C5 (Figure 2C and Table S1). There is considerably less variation in gene count than in repeat content (Figure 2B). The 18 gene sets allowed us to identify gene core families conserved in all sequenced *Dothidemycetes* as well as those evolving in species-specific manner. Identifying multi-gene families, we clustered all 215,225 predicted proteins in the *Dothideomycetes* into 42,182 families. Next, based on these families, predicted proteins were classified as being either unique to an organism, present in two or more *Dothideomycetes* (but not other *Ascomycota*), present in *Dothideomycetes* and other *Ascomycota* (but not in *Basidiomycota*), or present in *Ascomycota* and *Basidiomycota* (see Table 1 for the outgroups used). The overall pattern of conservation is very similar across *Dothideomycetes*, with the exception that species with a sequenced close relative have fewer unique proteins, as expected (Figure 2C).

**Figure 5:**
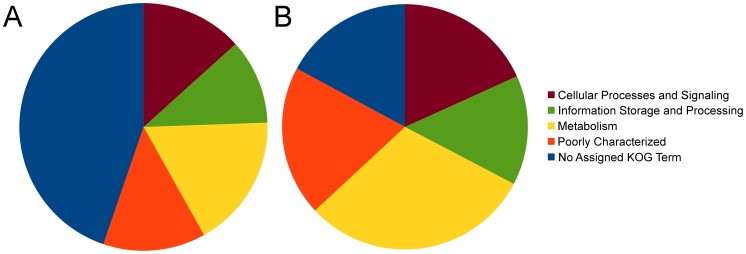
The full and core proteomes of the 18 *Dothideomycetes*. **A.** The full proteome of the *Dothideomycetes* contains 215,225 proteins and for the majority of these the function according to KOG [93] is unknown or poorly characterized. **B.** The core proteome contains the 66,761 proteins from multi-gene families that had at least one member in each *Dothideomycete*. Relative to (A), this set of proteins has more KOG annotations than the full proteome. In particular genes involved in metabolism are over-represented.

The core proteome was determined by identifying multi-gene families that contained at least one member in each of the *Dothideomycetes*. This resulted in 3,083 multi-gene families, containing a total of 66,761 proteins. Of these 3,083 families, 1,787 contained exactly 1 member in all *Dothideomycetes*, representing highly conserved single-copy gene families. The KOG annotations of the predicted proteins show that the core proteome is generally better annotated than the full set of proteins (Figure 5). Furthermore, proteins involved in metabolism are over-represented in the core proteome compared to the complete proteome. The proportion of the total proteome included in the core is indicated for individual *Dothideomycetes* in Figure S1. The counts of core proteins range from 3,884 in *M. populicola* to 4,811 in *R. rufulum*. Non-core proteins can give insight into species-specific processes. Functional annotation terms that are over-represented in the non-core proteome of the individual *Dothideomycetes* are given in Table S13. Numerous terms are under-represented in this set of proteins, including those related to metabolism (as expected), but also proteins with a transmembrane domain, peptidases, and glycoside hydrolase CAZymes. In contrast, small secreted proteins and carbohydrate esterase CAZymes are frequently over represented. These gene classes are further discussed below.

The availability of a large set of fungal genomes provides sufficient resolution for meaningful comparisons among groups of organisms based on phylogeny or lifestyle. Predicted proteomes from the 18 *Dothideomycetes* were compared to those of an outgroup consisting of 12 other *Ascomycota* and 9 *Basidiomycota*. Furthermore, taxonomic groups within the *Dothideomycetes* were compared to each other, as were groups based on lifestyle.

Although *Dothideomycetes* have few unique PFAM domains that are not found in the outgroup of 12 *Ascomycota* and 9 *Basidiomycota*, genes representing 233 PFAM domains are expanded in *Dothideomycetes* (Tables 3 and S14). Notable examples include a domain involved in signaling (response regulator receiver domain), metabolism (succinylglutamate desuccinylase/aspartoacylase domain), several glycoside hydrolases (see CAZY below) and a DNA photolyase domain.

**Table 3:** Summary of expansion and depletion of PFAM domains and multi-gene families in various comparisons based on phylogeny and lifestyle (Table 1).

	PFAM domain		Multi-gene families	
Comparison	Expanded (of which unique)	Depleted (of which absent)	Expanded (of which unique)	Depleted (of which absent)
*Dothideomycetes* versus ascomycete and basidiomycete outgroups	233 (2)	37 (3)	3358 (840)	280 (116)
Dothideomycete plant pathogens versus other plant pathogens	69 (10)	21 (9)	2098 (1411)	1209 (1081)
*Pleosporales* versus *Capnodiales*	137 (39)	67 (31)	2995 (2468)	2129 (1917)
Necrotrophic versus (hemi)biotrophic dothideomycete plant pathogens	4 (4)	21 (21)	299 (299)	1195 (1195)
Dothideomycete cereal pathogens versus other *Dothideomycetes*	6 (6)	14 (14)	492 (492)	359 (359)
Dothideomycete tree pathogens versus other *Dothideomycetes*	4 (4)	77 (77)	1220 (974)	2226 (2226)
Dothideomycete saprotrophs versus dothideomycete plant pathogens	7 (7)	25 (25)	516 (511)	551 (550)

All expanded and depleted PFAM domains and multi-gene families (as well as the statistics) are given in Tables S14 and S15, respectively.

Comparison of the dothideomycete plant pathogens to other fungal plant pathogens reveals 10 PFAM domains that are unique to *Dothideomycetes* pathogens and 69 PFAM domains that are expanded (Tables 3 and S14). This set includes a domain of the SUR7/PalI family (which is believed to be a membrane-bound sensor), a mannose-6-phosphate receptor domain and several domains of unknown function (DUFs). Although the exact roles of these proteins are currently unknown, they may be involved in a dothideomycete-specific strategy of pathogenesis.

The proteomes of the *Capnodiales* and the *Pleosporales* differ in part with respect to peptidases (see also proteases) and glycoside hydrolases (see also CAZY).

Cereal pathogens contain a lipase domain that is absent in other *Dothideomycetes*, as well as a putative DNA binding domain (DDT). Although these differences could be explained by phylogeny (most cereal pathogens analyzed except for *M. graminicola* belong to the *Pleosporales*) they are an interesting class of genes to investigate further. Tree pathogens are enriched in a specific hydrolase, whereas saprotrophs are enriched in a specific peptidase (Tables 3 and S14).

Since the PFAM database only contains previously described domains, novel gene families can be missed. For this reason, the same comparisons as above were made for multi-gene families that were identified based on similarity followed by Markov clustering. The resulting numbers are higher than for the PFAMs (Tables 3 and S15). Frequently these multi-gene families have no functional annotations assigned to them. For example, 3,358 multi-gene families are expanded in *Dothideomycetes*, compared to the outgroups used. Of those, 1,360 (41%) have no PFAM domains assigned to them, meaning that they contain mostly novel proteins. This again shows that *Dothideomycetes* contain many unique and novel proteins that may be involved in their specific lifestyle and strategy of pathogenesis.

Below we discuss specific classes of genes that have been shown to be involved in plant pathogenesis: small secreted proteins, genes involved in secondary metabolism, carbohydrate-active enzymes, peptidases, and lipases. In addition to these, kinases are discussed in Text S2, Table S16 and Figure S2.

### Small secreted proteins and candidate effectors

It is apparent that some small secreted proteins (SSPs) play an important role in plant-fungus interactions [35], [36]. SSPs were identified in the genomes of the 18 *Dothideomycetes* and of the outgroups (Table S17). Counts varied from 67 in the saprotroph *B. compniacensis* to 251 in *C. heterostrophus C4* and are within a similar range as other members of the *Ascomycota* (from 50 in *Saccharomyces cerevisiae* to 389 in *Magnaporthe grisea*) and *Basidiomycota* (from 40 in *Cryptococcus neoformans* to 540 in *Melampsora laricis-populina*) when using 200 amino acids as the upper limit for protein size. The three saprotrophs (*B. compniacensis*, *R. rufulum* and *H. pulicare*) are among the *Dothideomycetes* with the lowest number of predicted SSPs, confirming that SSPs are likely to be involved in plant-pathogen interactions. The *Pleosporales* generally have higher numbers of SSPs than the *Capnodiales*, which is also illustrated by the estimated numbers of SSPs in the last common ancestor of these respective taxonomic groups (189 and 134 for *Pleosporales* and *Capnodiales*, respectively [Figures 2D and S3]).

Of the predicted SSPs in *Dothideomycetes*, 8.3% had at least one PFAM domain. This is less than the 51.6% for all proteins, reflecting the fact that the function of SSPs is frequently unknown. The percentage of cysteine residues in the SSPs was higher than in the other proteins. Of all proteins, 9.8% resided in a singleton orthologous cluster (i.e., gene families with only one protein from only one organism). For the predicted SSPs this amount was 21.3%, reflecting the fact that this class of proteins is frequently species-specific.

### Secondary metabolism

Secondary metabolites were among the first factors shown to be required for virulence and host specificity of necrotrophs in the *Dothideomycetes* [37], [38]. Filamentous ascomycete genomes, including those of the *Dothideomycetes*, carry large numbers of genes encoding enzymes for secondary metabolite production (nonribosomal peptide synthetases (NPS), polyketide synthases (PKS) and terpene synthases (TPS) [39], [40]), in contrast to genomes of early diverging ascomycetes (yeasts) and basidiomycetes (Figure S4 and Table S18). With this in mind, we screened the 18 genomes for counterparts of highly curated *C. heterostrophus* NPSs, PKSs and the less well studied TPSs and found that most were not conserved and thus there is extreme diversity among species (Tables S19, S20, 21). This distribution supports the hypothesis that the metabolites biosynthesized by these enzymes are good candidates for involvement in species diversification, virulence, and/or host-specificity.

Generally, the numbers of genes encoding enzymes for secondary metabolite production are more numerous in the *Pleosporales* and *Hysteriales* than in the *Capnodiales* (Figures 2D and S3), and this is especially the case for the PKSs. This is also illustrated by the estimated number of genes encoding enzymes for secondary metabolite production in the respective last common ancestors of the *Pleosporales* (40 genes), *Hysteriales* (46 genes), and *Capnodiales* (24 genes).

The numbers of NPSs were high in the 18 *Dothideomycetes*, ranging from a low of 2 in the saprotroph *B. compniacensis* to a high of 44 in *P. teres* f. *teres*. These numbers were also high in the *Sordariomycetes*, *Eurotiomycetes*, and *Leotiomycetes*, in contrast to numbers in the yeasts and basidiomycetes (Figure S4 and Table S18). Numbers are higher in *Pleosporales* and *Hysteriales* than in *Capnodiales* (with the exception of *A. brassicicola*). In general, there are only a few fully conserved *NPS* genes/proteins across the fungi [39], including the 18 dothideomycete genomes examined here (Table S19). Only NPS10 (unknown product, mutants of *C. heterostrophus* are sensitive to oxidative stress) was perfectly conserved across the 18 dothideomycete genomes, in agreement with the earlier hypotheses [39] that NPS10 is among the more ancestral NPSs.

The next most highly conserved NPS (present in 17 of the 18 *Dothideomycetes*) is the counterpart of *C. heterostrophus* NPS2 (responsible for siderophore biosynthesis and intracellular iron storage). The latter is a critical cellular function, presumably required to prevent the Fenton reaction and concomitant accumulation of reactive oxygen species. Note that *B. compniacensis*, *C. fulvum*, *D. septosporum* and *M. populorum* have only two NPS orthologs each and that these are NPS10 and NPS2, discussed above. NPS2 proteins in *Pleosporales* and *Hysteriales* have four adenylating (AMP) domains, while those in the *Capnodiales* have three, similar to the other major groups of ascomycetes and basidiomycetes [41]. Next in degree of conservation is NPS4 (present in 10 of the 18 genomes, unknown product, *C. heterostrophus*, *A. brassicicola* and *F. graminearum nps4* mutant colonies are hydrophilic, rather than hydrophobic, like wild type [42]) and NPS6 (present in 11 of the 18 genomes; responsible for extracellular siderophore biosynthesis and thus competition for iron in the plant-fungal interaction). NPS6 has been shown to be involved in virulence of *C. heterostrophus* to corn, of *C. miyabeanus* to rice, of *A. brassicicola* to *Arabidopsis thaliana* and of *Fusarium graminearum* to wheat and for oxidative stress management (*in vitro*) [43].

The remaining *C. heterostrophus* NPS representatives are discontinuously distributed across the 18 genomes. The greatest conservation was found for members of the *Pleosporales*, and the fewest for the *Capnodiales*. These genes are known to be rapidly evolving and thus highly diverse, with a tendency to ‘pop up’ in disparate genomes. For example, the three-AMP-domain NPS for biosynthesis of *A. alternata* AM-toxin (Acc # BAI44739) has a perfect match in *M. graminicola* (JGI protein ID 56291) and the four-AMP-domain NPS, HTS1 (Acc # AAA33023), for *C. carbonum* HC-toxin biosynthesis, has orthologs in *S. turcica* and *P. tritici repentis* [44].

The numbers of Type I PKSs ranged from two in the saprotroph *B. compniacensis* to 34 in *R. rufulum* (Figure S4 and Table S18). Type III PKSs, known to be rare in filamentous fungi, had no members in the *Capnodiales* (with the exception of *M. graminicola*) and one member in the *Pleosporales*. Only one PKS protein, responsible for melanin biosynthesis, was universally conserved in all 18 genomes (with the exception of *A. brassicicola*) (Table S20). For many fungi, melanin is a virulence determinant [45], [46]. PKS1 and PKS2, required for T-toxin production and high virulence on maize, are found only in *C. heterostrophus* race T (strain C4). PKSs 4, 7, 20 and 25 are found only in all *C. heterostrophus* strains, while PKS11 and PKS24 were found in *C. heterostrophus* and *C. sativus* only. *C. heterostrophus* PKS24 is a hybrid NPS∶PKS (NPS7∶PKS24) and the entire protein is present in *C. sativus* (i.e., the NPS component is also present (Table S19). Some *C. heterostrophus* PKS orthologs (PKS6, 10, 13, 14, 16, 17, 21 and 22) were not present in *C. sativus*, yet were present in other species. With few exceptions (e.g., PKS17, which is present in *M. graminicola* and not other species in the *Pleosporales*), species that carried these genes tended to be those with a closer phylogenetic relationship.

The pattern of distribution of TPSs follows that of NPSs and PKSs in that few are conserved across the 18 *Dothideomycetes*. Most highly conserved is the *C. heterostrophus* protein ID 1098898, which shows >80% identity in all the genomes of *Pleosporales* and *Hysteriales*, but not of *Capnodiales* (Table S20). The best blast hit for this protein is lanosterol synthase, described as an integral membrane protein associated with the cytosolic side of the endoplasmic reticulum in eukaryotes. To our knowledge none of these TPSs has been functionally characterized in any of the 18 dothideomycete genomes and they thus represent untapped candidates for roles in species specificity, host specificity and/or virulence.

An example of a well described secondary metabolite pathway in *Dothideomycetes* is the biosynthesis of dothistromin [47], [48], [49], [50], [51]. Analyses with a core set of *D. septosporum* dothistromin genes suggested that only two of the other dothideomycete species, *C. fulvum* (sister species to *D. septosporum*) and *R. rufulum*, have a putative orthologous gene set (Text S4 and Figure S5), showing that it is discontinuously present across relatively distantly related *Dothideomycetes*.

The power of availability of multiple genomes for comparison cannot be over emphasized for fast-evolving genes such as those involved in secondary metabolism. Given that *PKS*, *NPS* and *TPS* orthologs are discontinuously distributed across genomes [39], [40] (Tables S19, S20, S21), a larger dataset is likely to uncover more orthologs in distantly related fungi. The debate continues regarding whether the tendency for duplication (gain) and loss, and recombination, coupled with the fast-evolving nature of these genes which erases evolutionary origin (for example due to RIP in the proximity of TE repeats, see above), are the basis of spotty distribution or whether there is support for the notion of horizontal transfer. We suggest both are likely.

### Carbohydrate-active enzymes

Plant cell wall polysaccharides function both as a physical barrier to plant pathogens and as a carbon source for plant pathogens and saprotrophs alike. Because of the enormous structural and functional diversity of these complex carbohydrates, the enzymes involved in their breakdown show a remarkable functional diversity. Carbohydrate-active enzymes (CAZymes) such as glycoside hydrolases (GH), polysaccharide lyases (PL) and carbohydrate esterases (CE), and CAZyme components such as the carbohydrate-binding modules (CBMs) therefore represent powerful reporters of the lifestyle of fungi, because (i) the latter achieve the digestion of complex carbohydrates extracellularly and (ii) sequence-based families of CAZymes correlate with structural and functional properties, although precise substrate specificities can be hard to predict [52]. In fact, whilst the sequence-based families of CAZymes frequently group together enzymes of varying substrate specificities, the functional correlation is often improved when considering broad substrate categories, especially among the different classes of plant polysaccharides (cellulose, hemicellulose, pectin). We have thus probed the CAZyme repertoires of the 18 *Dothideomycetes* to obtain clues to their digestive potential, especially against plant cell wall polysaccharides.

Table S22 shows that the genomes of the 18 examined *Dothideomycetes* encode almost 6,000 catabolic CAZyme catalytic domains (GHs, PLs, CEs) and CBMs but only 1,700 glycosyltransferases (GTs) involved in the assembly of fungal cell wall polysaccharides, N- and O-glycoproteins and reserve carbohydrates. The GTs, which assume roles that are not directly connected to the external environment show much less variation among the 18 genomes (min 83, max 110, average 96) than the digestive components: GHs (min 156, max 292, average 238); PLs (min 0, max 23, average 10); CEs (min 14, max 51, average 35); CBMs (min 17, max 101, average 48).

**Figure 6:**
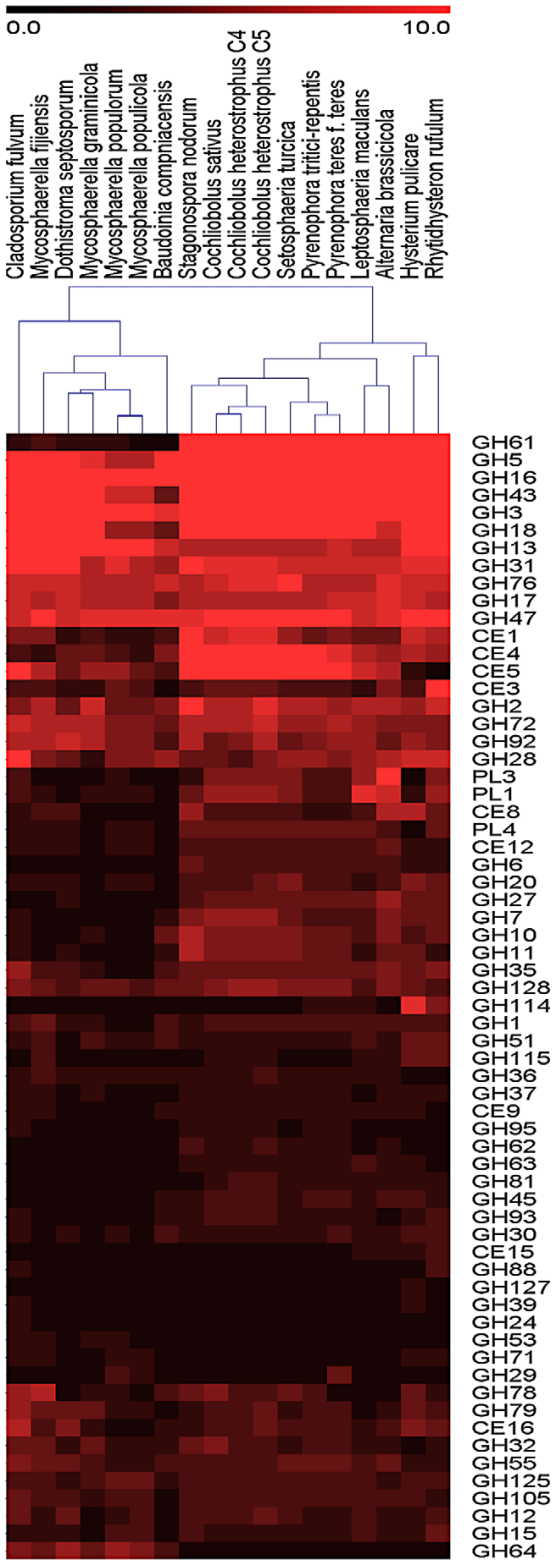
Heat map of CAZY families in the *Dothideomycetes*. Both the CAZY families and the organisms are hierarchically clustered. The clustering of organisms largely follows the phylogeny in Figure 2A. Notable exceptions are the observation that the biotroph *C. fulvum* clusters as an outgroup to the hemibiotrophs and saprotroph within the *Capnodiales*, and the observation that the two pathogens of *Brassica* spp. (*L. maculans* and *A. brassicicola*) cluster together.

Generally, the numbers of CAZymes are higher in the *Pleosporales* and *Hysteriales* than in the *Capnodiales* (Figure 2D). At the individual family level (Table S22) the differences are even more striking and hierarchical clustering based on CAZyme family numbers (Figure 6) divides the 18 genomes into two major groups, with the *Capnodiales* on one side and the *Hysteriales* and *Pleosporales* on the other. The division into these two groups is dominated by differences in the number of CAZymes acting on cellulose. The strongest difference is found with family GH61 (enzymes performing oxidative cleavage of cellulose [53]), where *Hysteriales* and *Pleosporales* have an average of 24 genes (min 20, max 30) but *Capnodiales* have between one and three only (Table S22). GH61 is not the sole cellulolytic family affected as families GH6, GH7, GH45 and CBM1 also show a clear expansion in *Pleosporales* and *Hysteriales* compared to *Capnodiales*, suggesting that the latter order of *Dothideomycetes* (containing mostly hemibiotrophs) does not extensively digest cellulose or that it employs another strategy for its digestion. This situation is reminiscent of the white rot/brown rot dichotomy [54], [55].

The difference between *Capnodiales* and other *Dothideomycetes* extends to the digestive enzymes directed against the other plant cell wall polysaccharides, specifically xylan and pectin. For instance, the two xylanase families GH10 and GH11 and the two acetylxylan esterase families CE1 and CE3 are significantly expanded in *Pleosporales* and *Hysteriales* compared to *Capnodiales* (Table S22). Patterns of enzymes involved in pectin digestion show a similar pattern, as the pectate lyases (families PL1 and PL3) and pectin methylesterases (family CE8) are expanded in *Pleosporales* (average 14.1 genes) compared to *Capnodiales* (average 6.0 genes) (Table S22). Also, *Capnodiales* encode fewer proteins with family CBM18 chitin-binding domains, than *Pleosporales* and *Hysteriales*. Chitin being produced by fungi and not plants, the mutiplication of these domains perhaps reflects different strategies of *Dothiodeomycetes* to evade recognition by plant defence mechanisms as shown for *C. fulvum* [36], [56], [57] and proposed for *M. graminicola* [24].

Not all CAZymes are under-represented in the *Capnodiales*. For example, family GH64 is more abundant in *Capnodiales* (av. 4.7) than in *Pleosporales* and *Hysteriales* (av. 1), and family GH114 is more abundant in the two *Hysteriales* saprotrophs (av. 7 genes) than *Pleosporales* (av. 1.6) and *Capnodiales* (av. 0.4). No fungal enzyme from these families has been characterized so far.

Altogether, genome mining revealed that the overall distribution of genes encoding enzymes for plant cell wall digestion globally follows the taxonomical division of *Capnodiales* and *Pleosporales*, and that it probably corresponds to different strategies for (or extents of) the breakdown of cellulose, as well as xylan and pectin. Constraints perhaps just as important as the precise composition of plant cell walls may well have shaped the carbohydrate-active enzyme profile of *Dothideomycetes*, such as the strategy of penetration through the outer layers of plant tissues, the strategy to break down crystalline cellulose and the strategy to evade plant defense mechanisms.

### Peptidases

Peptidases are important hydrolytic enzymes in plant pathogens that may have roles in signaling, nutrition, degradation of host plant tissues and digestion of proteins involved in the plant response against pathogens [58], [59], [60], [61]. Peptidase-encoding genes were catalogued in the predicted proteomes of the 18 genomes of *Dothideomycetes* and those of 21 other *Ascomycota* and *Basidiomycota* species according to the MEROPS database (Table S23, Figure S6). Secreted peptidases were studied separately, since these are more likely to be involved in pathogen-host interactions.

*Dothideomycetes* have a larger range of different exo- and endo-peptidases than plant pathogens found in other fungal classes (Figure S7A) [62]. These proteins include several secreted peptidases of the MEROPS subfamilies A01, S08, S09 and S10 expected to efficiently digest proteins and/or with an acidic optimum able to work in inhospitable environments of the extracellular matrix (A01, C13, G01, M35, M20 and S10) (Table S23, Figure S7B) [62]. The genomes of the *Dothideomycetes* contain fewer non-secreted and secreted aspartic peptidases (A01) than those of the plant-pathogenic necrotrophs of the *Leotiomycetes* (*Botrytis cinerea* and *Sclerotinia sclerotiorum*) and the saprotrophs and ectomycorrhizal symbionts of the *Agaricomycetes* (*C. cinereus*, *P. chrysosporium*, *P. placenta*, *S. commune* and *L. bicolor*), but this is compensated for by having the highest content in secreted metallo- (carboxypeptidases of the M14 subfamily and exopeptidases M28) and serine-peptidases of the carboxypeptidases S10 subfamily (Table S23, Figure S7AB). Within the *Dothideomycetes* the *Pleosporales* are specifically enriched in zinc-metallopeptidases of the M14 (5–9 models *vs.* 0–4 in *Capnodiales* and *Hysteriales*) and M28 subfamilies (10–13 models in *Pleosporales vs.* 6–9 in the others). Among the M14 secreted carboxypeptidases, three that are found in all members of the *Pleosporales* (and also found in the *Sordariomycetes*) have been lost in all of the fungi belonging to the orders *Hysteriales* and *Capnodiales* (Table S23, Figure S8).

Secreted zinc-metallopeptidases as well as trypsin (S01) and subtilisin (S08 and S53) serine-peptidases are known to have a potential role in pathogenicity and to be putatively involved in direct cell wall degradation by plant pathogens, as hydroxyproline-rich glycoproteins are possible targets of these enzymes [63], [64], [65]. Trypsin-like peptidases are limited in many fungal genomes to only one to five models found in each species, whereas plant pathogens are generally enriched in subtilisin-like proteins. Interestingly, within *Dothideomycetes* genes encoding secreted S08 subtilisin-like proteins is lower in the *Capnodiales* and *Hysteriales* (average 4.8 models *vs.* 7.3 in *Pleosporales*), whereas genes encoding aorsin and grifolisin-like peptidases of the S53 subfamily are higher (average 5.7 models *vs.* 2.3 models in *Pleosporales*). The selection of specific subfamilies of peptidases in each of these fungal orders suggests that differences in the properties of the enzymes could have provided functional advantages to their respective common ancestors.

Another notable difference within the *Dothideomycetes* is that the genomes of the wheat pathogen *M. graminicola* and poplar pathogens *M. populurum* and *M. populicola* encode more oligopeptidases of the M03B subfamily (six for *M. populorum*, four for *M. populicola* and 18 for *M. graminicola*) than any other fungus analyzed.

### Lipases

Several lipases are known to play important roles in plant pathogenicity. Fungal pathogens secrete lipases and cutinases that catalyze the hydrolysis of ester bonds from fatty acid polymers, facilitating fungal penetration through the cuticle [66], [67]. A genome-wide analysis of lipase-encoding genes among the *Dothideomycetes* revealed that 14 families are conserved among these fungi, with considerable variations between species and taxonomical groups (Table S24, Figure S9). Secreted lipases are more likely to be involved in pathogen-host interactions than non-secreted lipases. Seven families of secreted lipases are conserved among the *Dothideomycetes*. Generally, *Pleosporales* and *Hysteriales* have higher numbers of lipases and secreted lipases than the *Capnodiales* (Figure 2D). This difference is most apparent in the cutinases, which are esterases capable of breaking the thick cutin protection of external plant tissues. While the examined pathogens have an average of 8.9 and 4.5 genes encoding secreted cutinases in the *Pleosporales* and *Capnodiales*, respectively, the saprotrophs have 0 to 3 secreted cutinases each. The same distribution pattern is observed for plant pathogens versus non-pathogens in the outgroup, and can be explained by the fact that cutinases serve to break through the plant surface. Although the role of cutinases in fungal pathogenicity stayed controversial for a long time, the relationship with pathogenicity has been proven in several knockout studies [67], [68]. Furthermore, there are several examples of cutinases playing various roles in the establishment of infection by being involved in spore attachment [69], [70], surface signaling [71], and dissolution of the plant cuticle during penetration [72].

### Enrichment of potential effector genes in proximity to Transposable Elements

As mentioned above, there is a large variation in numbers of transposable elements among species, from approximately 40% in *C. fulvum* [73] and *M. fijiensis* to almost no repeats in *B. compniacensis* (Figure 2B, Tables 4 and S25). Repeats are under-represented in genomes sequenced exclusively using Illumina technology due to limitations of the technology so are not directly comparable to those sequenced by other means. The majority of TEs (over 40% of repeat content) in most genomes are long terminal repeat (LTR) retrotransposons. DNA transposons and non-LTR retrotransposons are observed in smaller proportions, with predominantly *C. fulvum*, *L. maculans* and *M. fijiensis* showing a considerable percentage of their genomes being comprised of repeats of these types. The most frequently identified family of transposable elements is Gypsy (Table S25). All 18 *Dothideomycetes* have the same components of the silencing machinery encoded in their genomes (Table S26), so this does not offer an explanation for the differences in numbers of TEs.

**Table 4:** Summary of gene classes that are over-represented in repeat regions (i.e., the 2000 bp flanking predicted transposable elements).

	TE repeat content (%)	Over-representation of gene classes in repeat regions			
		Small secreted proteins	All secreted proteins	Secondary metabolism	Expanded orphan multi-gene families
*Alternaria brassicicola*	5.58			•	
*Baudoinia compniacensis*	0.4				
*Cladosporium fulvum*	44.24				
*Cochliobolus heterostrophus C5*	7.77	•	•		•
*Cochliobolus heterostrophus C4* (*)	0				
*Cochliobolus sativus*	5.44	•		•	•
*Dothistroma septosporum*	0.67				
*Hysterium pulicare* (*)	0.57				
*Leptosphaeria maculans*	30.93	•	•	•	•
*Mycosphaerella fijiensis*	38.97		•		
*Mycosphaerella graminicola*	11.66	•			•
*Mycosphaerella populicola*	20.81			•	
*Mycosphaerella populorum*	3.56	•	•		•
*Pyrenophora tritici-repentis*	11.44	•	•	•	•
*Pyrenophora teres f. teres* (*)	1.98				•
*Rhytidhysteron rufulum* (*)	0.18				
*Setosphaeria turcica*	11.16	•	•	•	•
*Stagonospora nodorum*	2.37		•		•

See also Table S27 for more information. Genomes labeled with an asterisk (*) have been sequenced exclusively using Illumina technology.

Repetitive sequences in fungal genomes have been shown previously to be a target of the Repeat Induced Point mutation (RIP) machinery [74], [75]. To analyze the effect of proximity of a gene to a repeat region, the RIP index of these genes was calculated as a function of distance to the repeat sequences. Only repeats that belonged to a known family of transposable elements were taken into account (Figure 2B).

**Figure 7:**
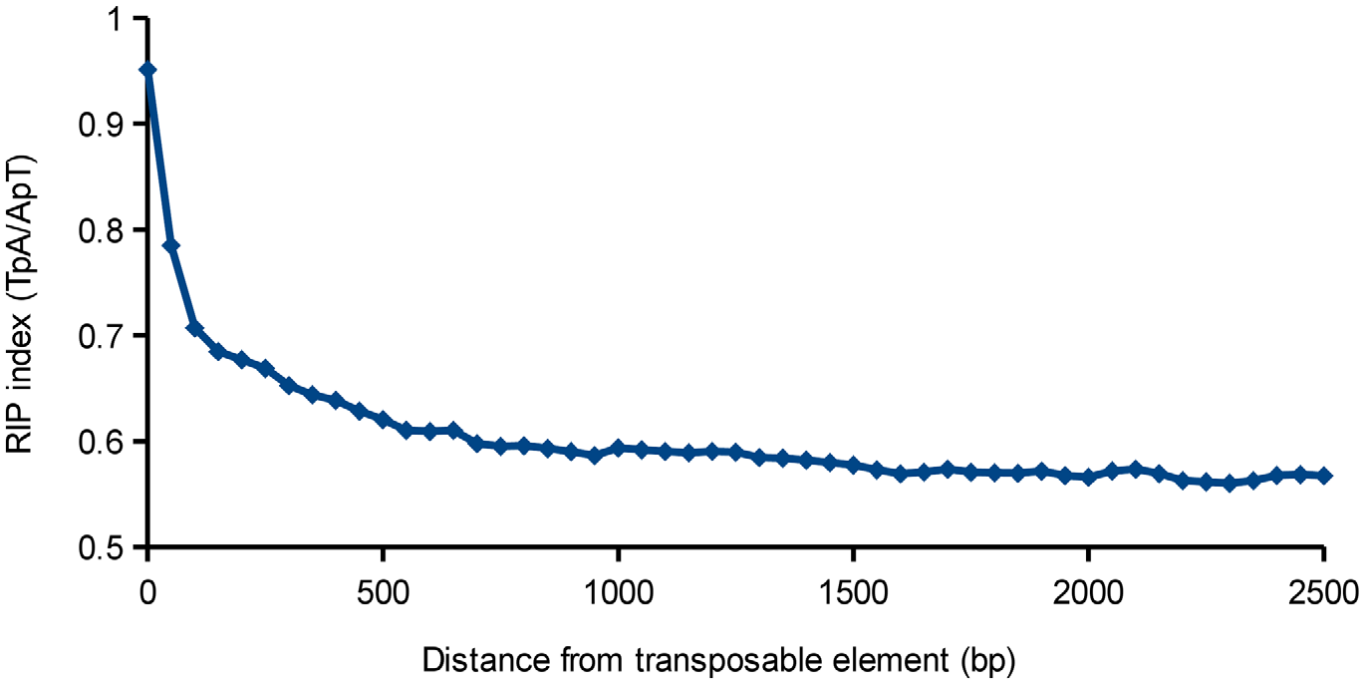
The RIP index (TpA/ApT) of genes as a function of the distance from a transposable element. The RIP index is highest near the transposable elements and levels off after approximately 2000 bp, signifying that these regions are subjected to repeat induced point mutations.

Overall, the closer a gene was located to a repeat, the more likely a RIP signature was detected (Figure 7). The RIP index TpA/ApT measures the frequency of TpA RIP products, correcting for false positives due to AT-rich regions. Higher values of the TpA/ApT RIP index indicate a stronger RIP response [74], [76]. Based on this index the effect is strongest within the first 500 bp nearest the repeat and then drops more slowly and disappears at approximately 2000 bp from the repeats.

Next, we determined what genes are over-represented in the 2000 bp around the repeats. Only genes that (at least partially) overlap this region, but that do not overlap a repeat were taken into account. This was done to exclude the pseudogenes that are frequently found inside TE repeat sequences, but were either included in gene sets or not depending on annotation strategy of the different sequencing centers. Interestingly, in several genomes the genes encoding small secreted proteins, proteins involved in secondary metabolism, or members of expanded orphan multi-gene families are over-represented in the regions flanking repeats compared to the rest of the genome (Tables 4 and S27). Most genomes with a TE content of at least 2% have at least one of these functional annotation terms over-represented in the flanking regions around repeats with the exception of *C. fulvum*, in which however several genes in the proximity of TEs were reported as affected by RIP [73]. An expanded orphan multi-gene family is defined here as a gene family with at least 2 members that is present in only one *Dothideomycete* and in none of the outgroups. Family members frequently include small secreted proteins, but relatively few PFAM domains (Table S28).

In *L. maculans*, AT-rich blocks composed of transposable elements were previously shown to occasionally harbor genes encoding small secreted proteins, and those genes were more subjected to RIP than other genes [23]. We show here that this is a widely occurring phenomenon among *Dothideomycetes*, although not universal. Our analysis shows that not only genes encoding small secreted proteins, but also genes involved in secondary metabolism are preferentially located in the vicinity of transposable elements. The products of some members of these classes of genes have been implicated as effectors in pathogenesis [36]. The potential evolutionary benefit of co-localization of repeat elements and effector genes is a higher rate of mutation due to RIP, which in turn may lead to a higher rate of evolution. This would allow the pathogen to adapt more quickly to the host plant's defenses. Furthermore, the observation that members of expanded orphan multi-gene families are over-represented near TEs suggests that TEs may have a function in species-specific gene family expansion in these organisms, presumably due to TE mobility.

### Conclusions

*Dothideomycetes* is one of the largest groups of fungal plant pathogens, the genomic sequences of which were largely unknown until now. Here we described 14 newly sequenced genomes of *Dothideomycetes* and compared them with each other, the four previously published *Dothideomycetes* and with 21 other previously sequenced fungal genomes. The 18 sequenced dothideomycete genomes are members of the three major orders of *Capnodiales*, *Pleosporales*, and *Hysteriales*, and represent a range of evolutionary distances within over 280 MYA since their common ancestor, as well as a variety of lifestyles and plant host associations. This added resolution makes it possible for the first time at such a large scale to explore genome organization, evolution, and differences between saprotrophic and the various modes of pathogenic lifestyles in *Dothideomycetes*.

There are large variations in genome size between the *Dothideomycetes*, which can be largely explained by the repetitive content of the individual genomes. Chromosome structural evolution in this class of fungi proceeds largely by intra-chromosomal rearrangements. A gradient of synteny from macro- to mesosynteny was observed in comparisons between species depending on evolutionary distance and agreed with simulation analyses of chromosomal evolution by frequent inversions. The high rate of inversions may be facilitated by the occurrence of simple repeats at the boundaries of inverted segments. Whether this phenomenon of frequent inversions is fortuitous or has been selected for to allow for rapid rates of evolution is not known. Gene order has not been completely reshuffled by these inversions, since blocks of genes with conserved order have been identified across *Dothideomycetes*. Their function and the reason for their conservation are currently unknown, but the observation that in one case in *L. maculans* all the genes in one conserved block of genes are up-regulated during plant infection suggests that co-regulation may be an important factor in pathogenesis.

A structural feature of the *Dothideomycetes* is the presence of seemingly dispensable chromosomes with no obvious function [24], [31], [32], [33]. Although dispensable chromosomes are known in other fungi, they usually are very few in number and have clear roles in niche adaptation, usually conditioning host specificity. Analyses of the 18 genomes of *Dothideomycetes* identified one to many scaffolds in multiple species that have the characteristics of dispensable chromosomes, so this phenomenon may occur commonly among the fungi in this class. Why and how these putatively dispensable chromosomes are maintained through long periods of evolutionary history is not known. However, the intra-chromosomal rearrangements leading to mesosynteny could keep dispensable chromosomes intact and may at least in part explain their apparent longevity.

The 18-genome comparative analysis also identified several functional adaptations of *Dothideomycetes* to their specific lifestyles. Genes encoding protein classes that were shown previously to play important roles as effectors in pathogenicity (e.g., enzymes for secondary metabolite production, carbohydrate-active enzymes, small secreted proteins, peptidases, and lipases) were found in all *Dothideomycetes*. However, large variations in these numbers exist between the different fungi. Generally, the *Pleosporales* and *Hysteriales* have higher numbers of these genes than the *Capnodiales*. This is also illustrated by the estimated gene counts of the respective last common ancestors for each of these groups. Possibly these last common ancestors each had different lifestyles or modes of pathogenesis. In the current set of organisms, necrotrophs are found exclusively in the *Pleosporales*, whereas six out of seven *Capnodiales* are (hemi)biotrophs. For a necrotroph, having a large arsenal of different types of effector genes presumably allows it to efficiently attack and kill the host plant in various ways. In contrast, (hemi)biotrophs spend an extended part of their life cycle in a stealth mode of pathogenicity, evading the host plant's defenses. In such a situation expressing a large arsenal of effectors could be detrimental, as it could lead to detection by the host plant and triggering of its defenses. The smaller set of effectors in members of the *Capnodiales* presumably allows them to evade this detection, as proposed previously for *M. graminicola* [24]. Another method would be to efficiently down-regulate these genes during stealth pathogenesis, which also may be the case for the three hemibiotrophs in the *Pleosporales*. An analysis of gene expression during the various stages of the life cycle should shed further light on this. For saprotrophs such as those in the *Hysteriales* having a large arsenal of these genes would be beneficial to efficiently obtain nutrients from their environment, and this is reflected in their gene complement. The extremophilic saprotroph *B. compniacensis* appears to have adopted a different strategy than other *Dothideomycetes* by reducing its genome size and complement of effectors.

In addition to the various modes of pathogenicity, we have identified numerous protein domains and multi-gene families that are expanded in pathogens of cereal or trees, when compared to the other Dothideomycetes. Their role is generally unknown, however. It should be noted that host plant specificity may be determined by a small set of genes and may therefore not show up in genome-wide comparisons. The exact role of these domains and of effectors in general cannot be predicted from large-scale comparative studies and require genome- or gene-focused analyses and experiments. However, an initial comparison of *in planta* transcriptomes can already suggest genes that may be important for pathogenicity.

We have shown that genes for effector proteins, previously shown to occur in AT-rich and gene-poor regions in the genome of *L. maculans* [23], occur often in close proximity to transposable elements (TEs) in several *Dothideomycetes*. TEs are frequently a target of Repeat Induced Point (RIP) mutations, and we have shown that RIP also occurs in the flanking regions surrounding these TEs. Co-localization of TEs and effector genes therefore exposes these genes to a higher rate of point mutations. This could possibly accelerate their rates of evolution and thereby provide an advantage in the arms race against their hosts. We also have shown that orphan multi-gene families (i.e. gene families with at least two members, but only found in one *Dothideomycete*) frequently co-localize with TEs. A possible explanation for this is that the high TE mobility rate functions as a driving force behind the duplication of these genes, allowing rapid species-specific gene family expansion and diversification.

As demonstrated by these results, the power of comparative genomics is huge and will become increasingly important as more genomes are sequenced. For fungi in general and *Dothideomycetes* in particular the field is ascending rapidly. Several other *Dothideomycetes* have been sequenced or are in progress and will provide greater representation of the extensive pathogenic and ecological diversity in this largest class of fungi. Completion of the 1000 Fungal Genomes project (http://jgi.doe.gov/fungi) at the US DOE Joint Genome Institute plus numerous genomes sequenced through other initiatives provide a huge wealth of virtually untapped resources for future progress in understanding fungal biology and evolution.

## Materials and Methods

### Data availability

All genome assemblies and annotations can be interactively accessed through the JGI fungal genome portal MycoCosm [77] at http://jgi.doe.gov/fungi. The 14 new Dothideomycete genomes discussed here are also deposited to DDBJ/EMBL/GenBank under the following accessions numbers *Alternaria brassicicola* ATCC 96836: ACIW00000000, *Baudoinia compniacensis* UAMH 10762: AEIF00000000, *Cladosporium fulvum* CBS131901 (race 0WU): AMRR00000000, *Cochliobolus heterostrophus* ATCC 48331 (race T, strain C4): AIHU00000000, *Cochliobolus heterostrophus* ATCC 48331 (race O, strain C5): AIDY00000000, *Cochliobolus sativus* ND90Pr: AEIN00000000, *Dothistroma septosporum* CBS128990 (NZE10): AIEN00000000, *Hysterium pulicare* CBS 123377: PRJNA81797, *Mycosphaerella fijiensis* CIRAD86: AIHZ00000000, *Mycosphaerella populicola* P02.02b: AIDU00000000, *Mycosphaerella populorum* SO2202: AEFD00000000, *Pyrenophora tritici-repentis* Pt-1C-BFP: AAXI00000000, *Rhytidhysteron rufulum* CBS 306.38: PRJNA81799, *Setosphaeria turcica* Et28A: AIHT00000000. The four additional genomes of *Leptosphaeria maculans* JN3 [23], *Mycosphaerella graminicola* IPO323 [24], *Pyrenophora teres f. teres* 0–1 [22], and *Stagonospora nodorum* SN15 [21] used in the comparative analyses were published earlier.

### Genome sequencing and assembly

The genomes of the 14 new *Dothideomycetes* discussed in this report were sequenced by several sequencing centers (Table 1) using various sequencing platforms and analyzed using different strategies, most of which are discussed in detail in genome-centric publications [44], [73], [78]. Additional genome-centric papers are planned for *M. fijiensis*, *C. heterostrophus C5*, *C. heterostrophus C4*, *C. sativus*, *S. turcica*, *M. populicola*, *M. populorum*, and *B. compniacensis*. The genomes of *A. brassicicola*, *C. heterostrophus* C5, *M. fijiensis* and *P. tritici-repentis* were sequenced using Sanger technology, assembled using Arachne [79], and improved using a genetic linkage map (*M. fijiensis*), optical map (*P. tritici-repentis*), or targeted finishing (*C. heterostrophus* C5). The genomes of *C. fulvum* and *M. populicola* were sequenced using 454 reads and assembled with Celera Assembler [73] and Newbler [80], respectively. The genomes of *C. heterostrophus* C4, *H. pulicare*, and *R. rufulum* were sequencing using Illumina technology only and assembled using ALLPATHS-LG (C4, [26]) and Velvet (both *Hysteriales*, [81]). The rest are hybrid assemblies sequenced using combinations of 454, Illumina and optionally Sanger reads, all assembled with Newbler [80]. Assembly characteristics are summarized in Figure 2B and Table S1.

### Genome annotation

Genomes sequenced by different organizations were annotated using different gene prediction pipelines. The *P. tritici-repentis* genome was annotated using the Broad Institute's pipeline [44], the *A. brassicicola* genome was annotated using an ENSEMBL annotation pipeline [78] and the *C. fulvum* assembled scaffolds were annotated using the Cyrille2 workflow management system [73]. For *H. pulicare* and *R. rufulum*, contigs greater than 300 bp were used for *ab initio* gene prediction in the software package Augustus [82] using the *Aspergillus fumigatus* gene predictions [83] for model guidance. The remaining 9 genomes were annotated using the JGI annotation pipeline, which combines multiple tools for gene prediction, annotation, and analysis, and deposits the results in the JGI Fungal Genome Portal MycoCosm (http://jgi.doe.gov/fungi) [77]. The assembled genomic scaffolds were masked using RepeatMasker [84] with the RepBase fungal library of 234 fungal repeats [85] and genome-specific libraries derived using RepeatScout [86] (see *Repeat content* below). Multiple sets of gene models were predicted for each assembly, and automated filtering based on homology and EST support was applied to produce a final non-redundant GeneCatalog representing the best gene model found at each genomic locus. The gene-prediction methods were: *EST-based predictions* with EST map (http://softberry.com) using raw ESTs and assembled EST contigs for each genome; *homology-based predictions* with Fgenesh+ [87] and Genewise [88], with homology seeded by BLASTx alignments of the GenBank non-redundant sequence database (NR: http://www.ncbi.nlm.nih.gov/BLAST/) to the genomic scaffolds; and *ab inito predictions* using Fgenesh [87]) and GeneMark [89]. Genewise models were extended to include 5′ start and/or 3′ stop codons when possible. Additional EST-extended sets were generated using BLAT-aligned [90] EST data to add 5′ UTRs, 3′ UTRs, and CDS regions that were supported by ESTs but had been omitted by the initial prediction methods.

The predicted gene models from the genomes of *Dothideomycetes* and the outgroups were functionally annotated using the same pipeline for each genome, allowing comparison across species. Functional annotation by similarity to genes from the GenBank non-redundant set using BLASTp alignments [91] and hardware-accelerated double-affine Smith-Waterman alignments (http://www.timelogic.com) against SwissProt (http://www.expasy.org/sprot), the Kyoto Encyclopedia of Genes and Genomes (KEGG) [92], and eukaryotic orthologous groups of proteins (KOG) [93]; analyzed for signal sequences and transmembrane domains with SignalP [94] and TMHMM [95]; and functional domains were predicted using InterProScan [96]. Enzyme commission (EC) numbers (http://www.expasy.org/enzyme) were assigned based on KEGG hits, and Gene Ontology (GO) terms [97] were assigned based on Interpro and SwissProt hits. Multi-gene families in all the species in Table 1 were predicted using the JGI clustering pipeline. First, an all-versus-all blastp analysis is performed using an E-value of 1e-5 as cut off value. Next, for each blastp hit pair a modified blast score is calculated: blast score * cov1 * cov2. Here, cov1 and cov2 are the alignment coverages for protein 1 and 2 of the pair, respectively. This alignment coverage is a fraction of 1. This modified blast score is used as input for the MCL Markov clustering program [98], [99], using an inflation parameter of 4. Each resulting cluster is considered a multi-gene family.

The functional annotations for all eighteen genomes are summarized in Figure 2 and in Table S1. In addition to this, repeats, lipases, CAZymes, peptidases, small secreted proteins, genes involved in secondary metabolism, and kinases in all 18 genomes were more extensively annotated as described below. The estimated gene counts of the last common ancestors of the taxa indicated in Figure 2D have been inferred using CAFÉ [100].

### Repeat content

For all 18 genomes RepeatScout [86] was used to generate *de novo* predictions of transposable elements (TE). The output of RepeatScout is a library of consensus sequences corresponding to each family of identified repeats. We selected all repeat families which had a Blastn (and Blastx) hits to Repbase sequences [85] and also included families with a copy number of more than 150 in the genome. All potential repeats were searched for structural elements usually found at termini of TE, such as LTRs (long terminal repeats), TIRs (terminal inverted repeats) and TSDs (target site duplications). TEs were classified by a combination of similarity to Repbase sequences [85] and availability of structural repeats according to the procedures outlined previously [101]. Simple repetitive sequences (i.e., low-complexity DNA regions) were identified using RepeatMasker [84].

For the Illumina-only genomes an additional analysis was performed to estimate repeat content, using the FindErrors module of the ALLPATHS-LG assembler [26]. Briefly, a k-mer histogram using K = 24 bp is constructed and partitioned into bins corresponding to likely sequencing error, unique genome sequence, possible polymorphism, possible repetitive genome sequence, and highly represented k-mers based on the locations of peaks in the k-mer histogram. The sum of the counts corresponding to the bin associated with repetitive content is reported as the estimate.

Over-representation of functional annotation terms in genes overlapping a 2-kpb flank upstream or downstream of a TE repeat (but that did not overlap any TE repeat) was calculated as described below. Genes overlapping TE repeats were excluded from this analysis because their predictions varied widely among the genomes; they were actively removed from some gene sets whereas they were left untouched in others. It was outside the scope of this paper to re-predict genes for each genome.

### Representation analysis

Custom scripts were developed in Python and R to analyze over- and under-representation of functional annotation terms in sets of genes using the Fisher Exact test. The Benjamini-Hochberg correction was used to correct for multiple testing using a *p*-value of 0.05, where applicable.

### Estimating phylogenetic relationships and divergence times

The broad phylogeny in Figure 1 was performed with partial sequences of translation elongation factor-1 alpha (*TEF1*) and the largest and second-largest subunits of DNA-directed RNA polymerase II (*RPB1, RPB2*). DNA sequences were downloaded from GenBank as indicated in Table S29 or obtained from genome data available at the Fungal Genome portal at JGI. Each of the individual genes was conceptually translated in BioEdit [102] after the introns were removed. The amino acid sequences were aligned in SATé [103] using MAFFT [104] as the external sequence alignment tool and RAxML [105] as the tree estimator. The final data matrix had 67 taxa and 1129 characters with 31% missing and gap characters. Two isolates of *Arthoniomycetes* (*Simonyella variegata* and *Opegrapha dolomitica*), the sister class to *Dothideomycetes*, were used as outgroups. The three protein sequence alignments were individually subjected to model testing with ProtTest v.2.4, using the Aikake information criterion (AIC) and default settings at http://darwin.uvigo.es/software/prottest2_server.html [106]. This resulted in a choice of the following models: TEF1: LG; RPB1: RTREV; and RPB2: LG. A maximum-likelihood phylogenetic analysis of the concatenated alignment with partitioned models for each gene marker was performed with a gamma model of rate heterogeneity at the CIPRES web portal [107] using RAxML v. 7.2.8 [105], [108]. Fifty maximum-likelihood (ML) searches were done, each one starting from a separate randomized tree, and the best-scoring tree was selected with a final likelihood score of −24058.182470. One thousand non-parametric bootstrap iterations were run and the resulting replicates plotted onto the best-scoring tree obtained previously. The RAxML tree was used to apply a penalized likelihood analysis in the program r8s v1.7 [109] to produce a chronogram. This meant that phylogenetic uncertainty was not incorporated in this analysis. We used two dates suggested in a more comprehensive analysis in previous work [110]. The root of the tree (and the split between *Dothideomycetes* and *Arthoniomycetes*) was set to ages of 420 and 309 MYA, respectively. These represent the upper and lower bound dates of a 95% confidence interval determined using a Bayesian approach in BEAST [111] as applied previously [110]. In our analysis the Langley-Fitch method and a truncated Newton method with bound constraints were applied following Taylor and Berbee [112].

For the genome-based tree in Figure 2, orthologous groups of genes (having exactly one gene for each organism in Table 1) were identified from the multi-gene family set described above. There were 51 of these orthologous groups and the corresponding proteins for the organisms belonging to the *Dothideomycetes* were concatenated. The sequences were aligned using MAFFT 6.717b [113] and well-aligned regions were extracted using Gblocks 0.91b [114]. The parallelized version of RAxML 7.2.8 [105], [108] with the PROTGAMMAWAG model with 100 rapid bootstrap partitions was used to calculate a species tree. The tree was visualized using Dendroscope 2.7.4 [115].

### Whole-genome DNA synteny

Whole-genome DNA synteny was calculated using VISTA [116] and visualized using the DotPlot function as implemented in the JGI Genome Portals [77]. Significance of whole-genome synteny was determined using the methods described previously [27], with modifications. For all combinations of genomes (genome A and genome B), all combinations of their sequences (sequence A from genome A and sequence B from genome B) were tested for significant sequence conservation. Only sequences of at least 500 kbp were used in these analyses. The probability of synteny (P_syn_) between sequence A of genome A and sequence B of genome B was calculated using a one-tailed cumulative binomial test described previously [27], with the modification that n = 100; x = (length conserved in sequence A * length conserved in sequence B)/(length of sequence A * length of sequence B) * n and rounded to the nearest integer; p = (Total length conserved in genome A * Total length conserved in genome B)/(Total length genome A * Total length genome B)/number of sequence pair combinations. A sequence pair was considered to have significant amounts of sequence conservation if P_syn_≥0.999. The determination of the whole-genome synteny and its type (mesosynteny or macrosynteny) were made using the significant pair ratio formula and the 20-kbp cut off, respectively [27]. The level of synteny degradation was computed using the pair exclusivity ratio [(Total length of conserved regions between sequences A and B)/(Total length of conserved regions for sequence A and all sequences of genome B+Total length of conserved regions for sequence B and all sequences of genome A)]. This formula is similar to that described previously [27] with modifications and the result is a fraction of 1. If the maximum value of all pair exclusivities of a genome pair was less than 0.75, synteny was classified as degraded.

### Gene order conservation

Gene order conservation across the 18 *Dothideomycetes* was studied using a custom script written in Python. Multi-gene families were determined as described above. Each gene of all organisms was thus assigned to a multi-gene family and orthologous relations between the genes could be determined. These relations can be one-to-one, one-to-many or many-to-many. The location of the first codon of the genes was used to determine the order of those genes on a given scaffold. Next, a sliding window with the size of 10 genes on a given scaffold of a given organism was compared to all possible windows on all scaffolds in all organisms. If a scaffold contained fewer than 10 genes, then all genes on that scaffold were considered in one window. For each comparison, the number of represented orthologous groups that was present in both windows was determined. If this number was at least 5, then these two windows were considered to be syntenic. If this syntenic window was present at least once in at least 70% of the studied organisms, then it was considered to be a conserved syntenic window. This analysis was done for all possible windows in all studied organisms. Next, overlapping conserved syntenic windows were combined into conserved syntenic blocks.

### Expansion and depletion of PFAM domains and multi-gene families

The expansion and depletion of PFAM domains and multi-gene families was determined by comparing genomes that were grouped according to phylogeny, host or lifestyle. In each comparison, an in-group of organisms was compared to an out-group. PFAM domains and multi-gene families were only included if they were present with at least one count in at least 50% of the organisms in at least one of these groups. If a PFAM domain or multi-gene family was unique to either the in-group or the out-group, or if it was expanded in either the in-group or the out-group (as determined by t-test and Wilcox rank test), then it was reported.

### Small secreted proteins

Small secreted proteins (SSPs) are defined here as proteins that are smaller than 200 aa, have a secretion signal as determined by SignalP 3.0 [94] and have no transmembrane domain (TMM) as determined by TMHMM 2.0 [117]. However, one transmembrane domain is allowed when present in the N-terminal 40 amino acids, since this often corresponds to the secretion signal. An SSP was labeled as ‘high cysteine’ when the percentage of cysteine residues in the protein was at least twice as high as the average percentage of cysteine residues in all predicted proteins of that organism.

### Secondary metabolism

The Hidden Markov Model (HMM) signatures previously described for the AntiSMASH pipeline [118] were used to identify and annotate putative polyketide synthase (*PKS*) and terpene synthase (*TPS*) genes in all 18 genomes after validation on the previously manually curated *C. heterostrophus* set. The same cut-off values and logic were applied. Nonribosomal peptide synthetase (*NPS*) encoding genes were identified using the method described by Bushley and Turgeon [39], since AntiSMASH [118] performed poorly on the previously manually curated set of *C. heterostrophus NPS* genes. In all cases we used the annotated *C. heterostrophus* proteins to query NPS, PKS, and TPS protein datasets extracted from the 18 dothideomycete genomes using a best-hit Blastp search.

### CAZyme annotation

The detection, module composition and family assignment of all carbohydrate-active enzymes was performed just as for the daily updates of the CAZy database (http://www.cazy.org) and described previously [52]. Briefly, the method combines BLAST and HMMer searches conducted against sequence libraries and HMM profiles made of the individual functional modules featured in the CAZy database. All positive hits were manually examined by human curators for final validation. For the heat map only the GH, PL and CE families were considered. Hierarchical clustering of both families and organisms was performed with the program MeV, which is part of the TM4 Software Suite [83]. Euclidian distance was used as distance metric and complete linkage clustering as linkage method. In Figure 6 only CAZymes with more than 1 member in at least one organism were included, for clarity.

### Peptidases

Peptidases were predicted from the protein model catalogs of 40 fungi (Table 1). For each fungal genome considered, the protein models were used as blastp query against full-length sequences of the Merops database (e-value = 1e-04) (release 9.5; http://merops.sanger.ac.uk). False positives were eliminated following unsuccessful searches against peptidase units and peptidase domains of the MEROPS (e-value = 1e-04) and the Pfam (V. 26.0; HMMER searches, e-value = 1.0) databases, respectively. Similarity of the models to putative peptidases was finally cross checked by parsing hits obtained following a blastp search (e-value = 1e-04) on the NCBI nr protein database. Prediction of putative secreted peptidases was then carried out using a combination of the SignalP 4.0 and TargetP 1.1 servers (http://www.cbs.dtu.dk/services/).

### Lipases

Putative lipases were classified according to BLASTp (E-value cut-off of 1e-04) results obtained against the Lipase Engineering Database (http://www.led.uni-stuttgart.de/). False positives were eliminated by parsing hits obtained for the presence of lipase-specific domains. Prediction of putative secreted lipases was processed as described above for secreted peptidases.

## Supporting Information

Figure S1 **Ratio of predicted core and non-core proteins in the 18 genomes of** ***Dothideomycetes*** **.**
(TIFF)

Figure S2 **Phylogeny and gene count of predicted casein kinase 1 (CK1) genes in the genomes of 18** ***Dothideomycetes*** **.**
(TIFF)

Figure S3 **Gene counts of classes that have been implicated in plant pathogenesis.** Gene counts in extant species (indicated right of the tree) and estimated gene counts in inferred last common ancestors (indicated on the tree nodes) are given. **A**. Genes encoding small secreted proteins. **B**. Genes encoding proteins involved in secondary metabolite production. **C**. Genes encoding carbohydrate-active enzymes (CAZymes). **D**. Genes encoding secreted peptidases. **E**. Genes encoding secreted lipases.
(TIFF)

Figure S4 **Predicted genes involved in secondary metabolism.** Genomes of organisms belonging to the *Pleosporales* and *Hysteriales* generally contain more genes involved in this process than the *Capnodiales*, especially in the case of Polyketide synthase Type I.
(TIFF)

Figure S5 **Presence of aflatoxin-like genes in** ***Dothideomycetes*** **and other fungi.** Aflatoxin-like genes were found using BLASTP (e-value of 10^−5^) of predicted amino acid sequences of *Dothistroma septosporum* dothistromin genes (main block of 14 *Ds* genes) or *Aspergillus flavus* sterigmatocystin (*verA* [*aflN*] and *omtB* [*aflO*]) or aflatoxin genes (*omtA* [*aflP*], *ordA* [*aflQ*]) (Yu et al 2004), against gene catalogues for all genomes shown, with reciprocal BLAST back to the *D. septosporum* or *A. flavus* genomes. Dark gray boxes indicate presence of a reciprocal best hit, light gray indicates one-directional best hit, and white indicates no hit. Dothideomycetes are *D. septosporum* (Ds), *Cladosporium fulvum* (Cf), *Mycosphaerella fijiensis* (Mf), *Mycosphaerella graminicola* (Mg), *Septoria musiva* (Sm), *Septoria populicola* (Sp), *Baudoinia compniacensis* (Bc), *Leptosphaeria maculans* (Lm), *Pyrenophora teres f. teres* (Pt), *Cochliobolus sativus* (Cs), *Cochliobolus heterostrophus* (Ch), *Setosphaeria turcica* (St), *Stagonospora nodorum* (Sn), *Pyrenophora tritici-repentis* (Pr), *Alternaria brassicicola* (Ab), *Rhytidhysteron rufulum* (Rr), *Hysterium pulicare* (Hp). Eurotiomycetes (Euro) are *Aspergillus nidulans* (An), *A. flavus* (Af). Leotiomycetes (Leo) are *Sclerotinia sclerotiorum* (Ss), *Botrytis cinerea* (Bc). Sordariomycetes (Sord) are *Verticillium dahliae* (Vd), *Magnaporthe grisea* (Mg), *Fusarium oxysporum* (Fo), *Nectria haematococca* (Nh), *Neurospora crassa* (Nc), *Trichoderma reesei* (Tr), *Chaetomium globosum* (Cg). Saccharomycotina (S) is *Saccharomyces cerevisiae* (Sc). Basidiomycete outgroups are the Agaricomycotina (Agar) *Postia placenta* (Pp), *Laccaria bicolor* (Lb), *Cryptococcus neoformans var. grubii* (Cn), *Coprinopsis cinerea* (Cc), *Schizophyllum commune* (Sc), *Phanerochaete chrysosporium* (Ph); Pucciniomycotina (Puc) *Puccinia graminis* (Pg), *Melampsora laricis-populina* (Ml); Ustilaginomycotina (Ust) *Malassezia globosa* (Mg), *Ustilago maydis* (Um). Protein ID numbers of query genes are 66976, 181128, 192192, 192193, 75691, 57312, 48495, 75546, 75609, 139960, 75656, 75692, 75547, 75566 (*D. septosporum* genome) and AFL2G_07219.2, 07216.2, 07215.2, 07214.2 (*A. flavus* genome). Note only *Ds-HexA, PksA, DotA, AdhA, VbsA and AflR* have been functionally confirmed as dothistromin genes by gene knockouts (Schwelm and Bradshaw 2010 and Bradshaw unpublished); other Ds- genes are considered to be dothistromin genes on the basis of similarity to AF genes and due to their proximities to known dothistromin genes in the *D. septosporum* genome. BLASTP of *Ds-HexA* and *Ds-HexB* both hit the same gene in each Agaricomycotina species showing gray shading. In contrast, the top matches of *Ds-HexA* and *Ds-HexB* were to a paralogous divergent pair of putative fatty acid synthase genes in all Dothideomycetes except Bc, Pt, St, Sn, Pr and all Sordariomycetes except Fo. See also Text S4.
(TIFF)

Figure S6 **Principal component analysis and hierarchical clustering of protease family assignments of 18** ***Dothideomycetes*** **and outgroups.** A matrix was constructed containing the number of proteases assigned to each MEROPS family in each fungal species. Analyses were performed with the Rcmdr package of R, and the results graphed along the first two components. Colored squares represent the centroid of each cluster of species, indicated with the same color. Taxonomy: Ag., Agaricales; Cap., Capnodiales; Dia., Diaporthales; Hel., Helotiales; Hyp., Hypocreales; Hys., Hysteriales; Mag., Magnaporthales; Mal., Malasseziales; Pat., Patellariales; Ple., Pleosporales; Pol. Polyporales; Sac., Saccharomycetales; Ure., Uredinales; Ust., Ustilaginales; Tre., Tremellales. Lifestyles: B., Biotroph; Ec., Ectomycorrhizal; En., Endophyte; OB., Obligate biotroph; S., Saprotroph; N., Necrotroph; H., Hemi-biotroph; U., Undetermined.
(TIFF)

Figure S7 **Distribution of peptidase families in** ***Dothideomycetes*** **and other fungal classes.** Putative peptidases were classified based on the MEROPS database. **A**. Non-secreted and secreted peptidases. **B**. Secreted peptidases. Letters above columns indicate significant differences between peptidase content means as determined by a Tukey's HSD test after significant one-way ANOVA.
(TIFF)

Figure S8 **Number of genes encoding peptidases in the genomes of** ***Capnodiales*** **,** ***Pleosporales*** **and** ***Hysteriales*** **fungi.**
(TIFF)

Figure S9 **Principal component analysis and hierarchical clustering of lipase family assignments of 18** ***Dothideomycetes*** **and outgroups.** Colored squares represent the centroid of each cluster of species, indicated with the same color. Taxonomy: Ag., Agaricales; Cap., Capnodiales; Dia., Diaporthales; Hel., Helotiales; Hyp., Hypocreales; Hys., Hysteriales; Mag., Magnaporthales; Mal., Malasseziales; Pat., Patellariales; Ple., Pleosporales; Pol. Polyporales; Sac., Saccharomycetales; Ure., Uredinales; Ust., Ustilaginales; Tre., Tremellales. Lifestyles: B., Biotroph; Ec., Ectomycorrhizal; En., Endophyte; OB., Obligate biotroph; S., Saprotroph; N., Necrotroph; H., Hemi-biotroph; U., Undetermined.
(TIFF)

Table S1 **Assembly and annotation statistics of the genomes of 18** ***Dothideomycetes*** **described in this report.**
(XLS)

Table S2 **Classification of synteny type in comparisons of 18** ***Dothideomycetes*** **.** The genome of *Aspergillus nidulans* was used as an outgroup.
(XLS)

Table S3 **Simple repeats are over-represented near the inversion breakpoints in relatively closely related mesosyntenic scaffold pairs.** The number of breakpoints that has at least one simple repeat within 500 bp distance is significantly higher than can be explained by chance.
(XLS)

Table S4 **Two syntenic blocks of genes are identified in the genomes of the** ***Dothideomycetes*** **.** Genes are located sequentially on the scaffolds. Each protein ID represents one predicted gene. The cluster ID represents the ID of the multi-gene family to which each gene belongs. Each gene that is part of a syntenic block has a syntenic ID.
(XLS)

Table S5 **Annotations of the genes in the two syntenic blocks from Table S4.** Each syntenic block contains 5 multi-gene families.
(XLS)

Table S6 **Orthologous pairs of genes between** ***Mycosphaerella graminicola*** **and** ***Leptosphaeria maculans*** **that are up- or down-regulated in both organisms when expression during relatively early infection is compared to expression relatively late in infection.** Each row represents an orthologous pair of genes. The protein IDs, expression ratio, GO annotation and PFAM annotation of each gene are indicated.
(XLS)

Table S7 **Functional annotation terms that are over- or under-represented in genes that are part of an orthologous pair that is co-regulated in** ***Mycosphaerella graminicola*** **and** ***Leptosphaeria maculans*** **.** See also Table S6.
(XLS)

Table S8 **Syntenic blocks of genes are identified in the genomes of eight** ***Pleosporales*** **.** Genes are located sequentially on the scaffolds. Each protein ID represents one predicted gene. The cluster ID represents the ID of the multi-gene family to which each gene belongs. Each gene that is part of a syntenic block has a syntenic ID.
(XLS)

Table S9 **Syntenic blocks of genes are identified in the genomes of six** ***Mycosphaerellaceae*** **.** Genes are located sequentially on the scaffolds. Each protein ID represents one predicted gene. The cluster ID represents the ID of the multi-gene family to which each gene belongs. Each gene that is part of a syntenic block has a syntenic ID.
(XLS)

Table S10 **Functional annotation terms that are over- or under-represented in genes that are located in at least one syntenic block (described in Table S8).** For each species the over-represented terms are indicated.
(XLS)

Table S11 **Functional annotation terms that are over- or under-represented in genes that are located in at least one syntenic block (described in Table S9).** For each species the over-represented terms are indicated.
(XLS)

Table S12 **Additional statistics on the putatively dispensable chromosomes.**
(XLS)

Table S13 **Functional annotation terms that are over- or under-represented in genes of which the product is not part of the core proteome.**
(XLS)

Table S14 **PFAM domains that are expanded or depleted in the various comparisons in** **Table 3** **.** In each comparison the organisms are marked as part of either the in-group or the out-group. Note that each comparison has its own tab.
(XLS)

Table S15 **Multi-gene families that are expanded or depleted in the various comparisons in** **Table 3** **.** In each comparison the organisms are marked as part of either the in-group or the out-group. Note that each comparison has its own tab.
(XLS)

Table S16 **Predicted kinases in the genomes of the 18** ***Dothideomycetes*** **and in those of the outgroups.** For each kinase type in each genome, the absolute number is indicated, as well as the percentage of total kinases in that genome. CAMK (calmodulin-regulated kinases); CMGC family (cyclin-dependent kinases, mitogen-activated protein kinases, CDK-like kinases, and glycogen synthase kinase); STE (including many kinases functioning upstream of the MAPKs); AGC (including cyclic-nucleotide and calcium-phospholipid-dependent kinases, ribosomal S6-phosphorylating kinases, G protein-coupled kinases, and related kinases); CK1 family (casein kinase 1, and close relatives); the TK family (tyrosine kinases); and the TKL family (tyrosine kinase-like kinases (TKLs), which resemble TK but are actually serine-threonine kinases).
(XLS)

Table S17 **Statistics of predicted small secreted proteins (SSPs) in the genomes of the 18** ***Dothideomycetes*** **and in those of the outgroups.**
(XLS)

Table S18 **Predicted genes involved in secondary metabolism.**
(XLS)

Table S19 **Distribution of nonribosomal peptide synthetases across the 18** ***Dothideomycetes*** **relative to the highly curated set in** ***Cochliobolus heterostrophus*** **set** [**39**] **.**
(XLS)

Table S20 **Distribution of polyketide synthases across the 18** ***Dothideomycetes*** **relative to the highly curated set in** ***Cochliobolus heterostrophus*** **set** [**40**] **.**
(XLS)

Table S21 **Distribution of terpene synthases across the 18** ***Dothideomycetes*** **relative to the** ***Cochliobolus heterostrophus*** **JGI set.** Cutoff is greater than 70% identity at the amino acid level.
(XLS)

Table S22 **Individual carbohydrate-active enzyme (CAZyme) family counts in the genomes of 18** ***Dothideomycetes*** **and the outgroups.**
(XLS)

Table S23 **Predicted peptidases in the genomes of 18** ***Dothideomycetes*** **and in those of the outgroups used.** Both the full set of peptidases and the subset of secreted peptidases are indicated.
(XLS)

Table S24 **Predicted lipases in the genomes of** ***Dothideomycetes*** **.** Both the full set of lipases and the subset of secreted lipases are indicated.
(XLS)

Table S25 **Repetitive content and transposable element (TE) annotation for the 18** ***Dothideomycetes*** **.**
(XLS)

Table S26 **Presence of components of the repeat induced point mutations (RIP) and silencing machineries in the genomes of** ***Dothideomycetes*** **.** All *Dothideomycetes* contain the same complement of components. For each component in each genome the protein ID of the best blastp hit and the corresponding E-value are indicated. In some cases the gene was missed during gene prediction, and in these cases the genomic location of the best tblastn hit and the corresponding E-value are indicated.
(XLS)

Table S27 **Functional annotation terms that are over- or under-represented in genes that are located in regions up to 2 kbp from Transposable Elements.**
(XLS)

Table S28 **Statistics on members of expanded orphan multi-gene families.** An expanded orphan multi-gene family is defined here as a gene family with at least 2 members that is present in only one *Dothideomycete* and in none of the outgroups. Family members frequently include small secreted proteins, but relatively few PFAM domains
(XLS)

Table S29 **Accession numbers of sequences that were used to estimate the phylogenetic relationships between the 67 organisms in** **Figure 1** **.**
(XLS)

Text S1 **Additional considerations concerning** ***Dothideomycetes*** **phylogeny and divergence time estimates.**
(DOC)

Text S2 **Predicted kinases in the genomes of 18** ***Dothideomycetes*** **and in those of the outgroups used.**
(DOC)

Text S3 **Comparative transcriptomics during pathogenesis.**
(DOC)

Text S4 **Dothistromin and aflatoxin biosynthetic pathways in** ***Dothideomycetes*** **.**
(DOC)

## References

[1] Kirk P, Cannon P, Minter D, Stalpers J (2008) Ainsworth and Bisby's dictionary of the Fungi, 10th ed. Wallingford, UK: CAB International.

[2] Ruibal C, Gueidan C, Selbmann L, Gorbushina AA, Crous PW, et al (2009) Phylogeny of rock-inhabiting fungi related to Dothideomycetes. Stud Mycol 64: 123–133S127. 20169026 10.3114/sim.2009.64.06 PMC2816969

[3] Ewaze JO, Summerbell RC, Scott JA (2008) Ethanol physiology in the warehouse-staining fungus, Baudoinia compniacensis. Mycol Res 112: 1373–1380. 18951774 10.1016/j.mycres.2008.05.003

[4] Gioulekas D, Damialis A, Papakosta D, Spieksma F, Giouleka P, et al (2004) Allergenic fungi spore records (15 years) and sensitization in patients with respiratory allergy in Thessaloniki-Greece. J Investig Allergol Clin Immunol 14: 225–231. 15552717

[5] Suetrong S, Schoch CL, Spatafora JW, Kohlmeyer J, Volkmann-Kohlmeyer B, et al (2009) Molecular systematics of the marine Dothideomycetes. Stud Mycol 64: 155–173S156. 20169029 10.3114/sim.2009.64.09 PMC2816972

[6] Shearer CA, Raja HA, Miller AN, Nelson P, Tanaka K, et al (2009) The molecular phylogeny of freshwater Dothideomycetes. Stud Mycol 64: 145–153S144. 20169028 10.3114/sim.2009.64.08 PMC2816971

[7] Nelsen M, Lücking R, Mbatchou J, Andrew C, Spielmann A, et al (2011) New insights into relationships of lichen-forming Dothideomycetes. Fungal Diversity 51: 155–162.

[8] Spatafora J, Owensby C, Douhan G, Boehm E, Schoch C (2011) Phylogenetic placement of Cenococcum in Gloniaceae (Dothideomycetes). Mycologia 104: 758–65. 10.3852/11-233 22453119

[9] Ertz D, Tehler A (2011) The phylogeny of Arthoniales (Pezizomycotina) inferred from nucLSU and RPB2 sequences. Fungal Diversity 49: 47–71.

[10] Schoch CL, Sung GH, Lopez-Giraldez F, Townsend JP, Miadlikowska J, et al (2009) The Ascomycota tree of life: a phylum-wide phylogeny clarifies the origin and evolution of fundamental reproductive and ecological traits. Syst Biol 58: 224–239. 20525580 10.1093/sysbio/syp020

[11] Arnold AE, Miadlikowska J, Higgins KL, Sarvate SD, Gugger P, et al (2009) A Phylogenetic Estimation of Trophic Transition Networks for Ascomycetous Fungi: Are Lichens Cradles of Symbiotrophic Fungal Diversification? Systematic Biology 58: 283–297. 20525584 10.1093/sysbio/syp001

[12] Lumbsch HT, Huhndorf SM (2010) Myconet Volume 14. Part One. Outline of Ascomycota—2009. Part Two. Notes on Ascomycete Systematics. Nos. 4751–5113. Fieldiana Life and Earth Sciences 1–64.

[13] Zhang Y, Crous P, Schoch C, Bahkali A, Guo L, et al (2011) A molecular, morphological and ecological re-appraisal of Venturiales—a new order of Dothideomycetes. Fungal Diversity 51: 249–277. 22368534 10.1007/s13225-011-0141-x PMC3285419

[14] Hofmann T, Kirschner R, Piepenbring M (2010) Phylogenetic relationships and new records of Asterinaceae (Dothideomycetes) from Panama. Fungal Diversity 43: 39–53.

[15] Wu H, Schoch C, Boonmee S, Bahkali A, Chomnunti P, et al (2011) A reappraisal of Microthyriaceae. Fungal Diversity 51: 189–248. 22408574 10.1007/s13225-011-0143-8 PMC3293405

[16] Suetrong S, Boonyuen N, Pang K-L, Ueapattanakit J, Klaysuban A, et al (2011) A taxonomic revision and phylogenetic reconstruction of the Jahnulales (Dothideomycetes), and the new family Manglicolaceae. Fungal Diversity 51: 163–188.

[17] Schoch CL, Crous PW, Groenewald JZ, Boehm EW, Burgess TI, et al (2009) A class-wide phylogenetic assessment of Dothideomycetes. Stud Mycol 64: 1–15S10. 20169021 10.3114/sim.2009.64.01 PMC2816964

[18] Grigoriev IV, Cullen D, Goodwin SB, Hibbett D, Jeffries TW, et al (2011) Fueling the future with fungal genomics. Mycology 2: 192–209.

[19] McLaughlin DJ, Hibbett DS, Lutzoni F, Spatafora JW, Vilgalys R (2009) The search for the fungal tree of life. Trends Microbiol 17: 488–497. 19782570 10.1016/j.tim.2009.08.001

[20] Scott JA, Untereiner WA, Ewaze JO, Wong B, Doyle D (2007) Baudoinia, a new genus to accommodate Torula compniacensis. Mycologia 99: 592–601. 18065010 10.3852/mycologia.99.4.592

[21] Hane JK, Lowe RG, Solomon PS, Tan KC, Schoch CL, et al (2007) Dothideomycete plant interactions illuminated by genome sequencing and EST analysis of the wheat pathogen Stagonospora nodorum. Plant Cell 19: 3347–3368. 18024570 10.1105/tpc.107.052829 PMC2174895

[22] Ellwood SR, Liu Z, Syme RA, Lai Z, Hane JK, et al (2010) A first genome assembly of the barley fungal pathogen Pyrenophora teres f. teres. Genome Biol 11: R109. 21067574 10.1186/gb-2010-11-11-r109 PMC3156948

[23] Rouxel T, Grandaubert J, Hane JK, Hoede C, van de Wouw AP, et al (2011) Effector diversification within compartments of the Leptosphaeria maculans genome affected by Repeat-Induced Point mutations. Nat Commun 2: 202. 21326234 10.1038/ncomms1189 PMC3105345

[24] Goodwin SB, M'Barek S B, Dhillon B, Wittenberg AH, Crane CF, et al (2011) Finished genome of the fungal wheat pathogen Mycosphaerella graminicola reveals dispensome structure, chromosome plasticity, and stealth pathogenesis. PLoS Genet 7: e1002070. 21695235 10.1371/journal.pgen.1002070 PMC3111534

[25] Boehm EWA, Schoch CL, Spatafora JW (2009) On the evolution of the Hysteriaceae and Mytilinidiaceae (Pleosporomycetidae, Dothideomycetes, Ascomycota) using four nuclear genes. Mycological Research 113: 461–479. 19422072 10.1016/j.mycres.2008.12.001

[26] Gnerre S, Maccallum I, Przybylski D, Ribeiro FJ, Burton JN, et al (2011) High-quality draft assemblies of mammalian genomes from massively parallel sequence data. Proc Natl Acad Sci U S A 108: 1513–1518. 21187386 10.1073/pnas.1017351108 PMC3029755

[27] Hane JK, Rouxel T, Howlett BJ, Kema GH, Goodwin SB, et al (2011) A novel mode of chromosomal evolution peculiar to filamentous Ascomycete fungi. Genome Biol 12: R45. 21605470 10.1186/gb-2011-12-5-r45 PMC3219968

[28] Cozijnsen AJ, Howlett BJ (2003) Characterisation of the mating-type locus of the plant pathogenic ascomycete Leptosphaeria maculans. Current Genetics 43: 351–357. 12679880 10.1007/s00294-003-0391-6

[29] Wittenberg AH, van der Lee TA, Ben M'barek S, Ware SB, Goodwin SB, et al (2009) Meiosis drives extraordinary genome plasticity in the haploid fungal plant pathogen Mycosphaerella graminicola. PLoS ONE 4: e5863. 19516898 10.1371/journal.pone.0005863 PMC2689623

[30] Covert SF (1998) Supernumerary chromosomes in filamentous fungi. Current Genetics 33: 311–319. 9618581 10.1007/s002940050342

[31] Leclair S, Ansan-Melayah D, Rouxel T, Balesdent M (1996) Meiotic behaviour of the minichromosome in the phytopathogenic ascomycete Leptosphaeria maculans. Current Genetics 30: 541–548. 8939816 10.1007/s002940050167

[32] Tzeng TH, Lyngholm LK, Ford CF, Bronson CR (1992) A restriction fragment length polymorphism map and electrophoretic karyotype of the fungal maize pathogen Cochliobolus heterostrophus. Genetics 130: 81–96. 1346261 10.1093/genetics/130.1.81 PMC1204808

[33] Hatta R, Ito K, Hosaki Y, Tanaka T, Tanaka A, et al (2002) A conditionally dispensable chromosome controls host-specific pathogenicity in the fungal plant pathogen Alternaria alternata. Genetics 161: 59–70. 12019223 10.1093/genetics/161.1.59 PMC1462115

[34] Lagesen K, Hallin P, Rodland EA, Staerfeldt HH, Rognes T, et al (2007) RNAmmer: consistent and rapid annotation of ribosomal RNA genes. Nucleic Acids Res 35: 3100–3108. 17452365 10.1093/nar/gkm160 PMC1888812

[35] Martin F, Aerts A, Ahren D, Brun A, Danchin EG, et al (2008) The genome of Laccaria bicolor provides insights into mycorrhizal symbiosis. Nature 452: 88–92. 18322534 10.1038/nature06556

[36] Stergiopoulos I, Wit dPJGM (2009) Fungal effector proteins. Annual Review of Phytopathology 47: 233–263. 10.1146/annurev.phyto.112408.132637 19400631

[37] Yang G, Rose MS, Turgeon BG, Yoder OC (1996) A Polyketide Synthase Is Required for Fungal Virulence and Production of the Polyketide T-Toxin. The Plant Cell Online 8: 2139–2150. 10.1105/tpc.8.11.2139 PMC161341 8953776

[38] Panaccione DG, Scott-Craig JS, Pocard JA, Walton JD (1992) A cyclic peptide synthetase gene required for pathogenicity of the fungus Cochliobolus carbonum on maize. Proc Natl Acad Sci U S A 89: 6590–6594. 11607305 10.1073/pnas.89.14.6590 PMC49547

[39] Bushley KE, Turgeon BG (2010) Phylogenomics reveals subfamilies of fungal nonribosomal peptide synthetases and their evolutionary relationships. BMC Evol Biol 10: 26. 20100353 10.1186/1471-2148-10-26 PMC2823734

[40] Kroken S, Glass NL, Taylor JW, Yoder OC, Turgeon BG (2003) Phylogenomic analysis of type I polyketide synthase genes in pathogenic and saprobic ascomycetes. Proc Natl Acad Sci U S A 100: 15670–15675. 14676319 10.1073/pnas.2532165100 PMC307626

[41] Bushley K, Ripoll D, Turgeon BG (2008) Module evolution and substrate specificity of fungal nonribosomal peptide synthetases involved in siderophore biosynthesis. BMC Evolutionary Biology 8: 328. 19055762 10.1186/1471-2148-8-328 PMC2644324

[42] Turgeon BG, Oide S, Bushley K (2008) Creating and screening Cochliobolus heterostrophus non-ribosomal peptide synthetase mutants. Mycol Res 112: 200–206. 18280721 10.1016/j.mycres.2007.10.012

[43] Oide S, Moeder W, Krasnoff S, Gibson D, Haas H, et al (2006) NPS6, Encoding a Nonribosomal Peptide Synthetase Involved in Siderophore-Mediated Iron Metabolism, Is a Conserved Virulence Determinant of Plant Pathogenic Ascomycetes. The Plant Cell Online 18: 2836–2853. 10.1105/tpc.106.045633 PMC1626607 17056706

[44] Manning VA, Pandelova I, Dhillon B, Wilhelm LJ, Goodwin SB, et al.. (2012) Comparative Genomics of a Plant-Pathogenic Fungus, Pyrenophora tritici-repentis, Reveals Transduplication and the Impact of Repeat Elements on Pathogenicity and Population Divergence. G3. In Press. 10.1534/g3.112.004044 PMC3538342 23316438

[45] Guillen A, Turgeon BG, Thorson PR, Bronson CR, Yoder OC (1994) Linkage among melanin biosynthetic mutations in Cochliobolus heterostrophus. Fungal Genet Newsl 41: 41–42.

[46] Langfelder K, Streibel M, Jahn B, Haase G, Brakhage AA (2003) Biosynthesis of fungal melanins and their importance for human pathogenic fungi. Fungal Genetics and Biology 38: 143–158. 12620252 10.1016/s1087-1845(02)00526-1

[47] Assante G, Locci R, Camarada L, Merlini L, Nasini G (1977) Screening of the genus *Cercospora* for secondary metabolites. Phytochemistry 16: 243–247.

[48] Bradshaw RE, Jin HP, Morgan BS, Schwelm A, Teddy OR, et al (2006) A polyketide synthase gene required for biosynthesis of the aflatoxin-like toxin, dothistromin. Mycopathologia 161: 283–294. 16649078 10.1007/s11046-006-0240-5

[49] Shaw GJ, Chick M, Hodges R (1978) A ^13^C-NMR study of the biosynthesis of the anthraquinone dothistromin by *Dothistroma pini* . Phytochemistry 17: 1743–1745.

[50] Henry KM, Townsend CA (2005) Ordering the reductive and cytochrome P450 oxidative steps in demethylsterigmatocystin formation yields general insights into the biosynthesis of aflatoxin and related fungal metabolites. Journal of the American Chemical Society 127: 3724–3733. 15771506 10.1021/ja0455188

[51] Schwelm A, Bradshaw RE (2010) Genetics of dothistromin biosynthesis of *Dothistroma septosporum*: an update. Toxins 2: 2680–2698. 22069571 10.3390/toxins2112680 PMC3153176

[52] Cantarel BL, Coutinho PM, Rancurel C, Bernard T, Lombard V, et al (2009) The Carbohydrate-Active EnZymes database (CAZy): an expert resource for Glycogenomics. Nucleic Acids Res 37: D233–238. 18838391 10.1093/nar/gkn663 PMC2686590

[53] Harris PV, Welner D, McFarland KC, Re E, Navarro Poulsen JC, et al (2010) Stimulation of lignocellulosic biomass hydrolysis by proteins of glycoside hydrolase family 61: structure and function of a large, enigmatic family. Biochemistry 49: 3305–3316. 20230050 10.1021/bi100009p

[54] Eastwood DC, Floudas D, Binder M, Majcherczyk A, Schneider P, et al (2011) The plant cell wall-decomposing machinery underlies the functional diversity of forest fungi. Science 333: 762–765. 21764756 10.1126/science.1205411

[55] Baldrian P, Valaskova V (2008) Degradation of cellulose by basidiomycetous fungi. FEMS Microbiol Rev 32: 501–521. 18371173 10.1111/j.1574-6976.2008.00106.x

[56] Stergiopoulos I, van den Burg HA, Ökmen B, Beenen HG, van Liere S, et al (2010) Tomato Cf resistance proteins mediate recognition of cognate homologous effectors from fungi pathogenic on dicots and monocots. Proceedings of the National Academy of Sciences 107: 7610–7615. 10.1073/pnas.1002910107 PMC2867746 20368413

[57] van Esse HP, Bolton MD, Stergiopoulos I, de Wit PJGM, Thomma BPHJ (2007) The Chitin-Binding Cladosporium fulvum Effector Protein Avr4 Is a Virulence Factor. Molecular Plant-Microbe Interactions 20: 1092–1101. 17849712 10.1094/MPMI-20-9-1092

[58] Carlile AJ, Bindschedler LV, Bailey AM, Bowyer P, Clarkson JM, et al (2000) Characterization of SNP1, a cell wall-degrading trypsin, produced during infection by Stagonospora nodorum. Mol Plant Microbe Interact 13: 538–550. 10796020 10.1094/MPMI.2000.13.5.538

[59] Olivieri F, Eugenia Zanetti M, Oliva CR, Covarrubias AA, Casalongué CA (2002) Characterization of an Extracellular Serine Protease of Fusarium eumartii and its Action on Pathogenesis Related Proteins. European Journal of Plant Pathology 108: 63–72.

[60] Plummer KM, Clark SJ, Ellis LM, Loganathan A, Al-Samarrai TH, et al (2004) Analysis of a Secreted Aspartic Peptidase Disruption Mutant of Glomerella cingulata. European Journal of Plant Pathology 110: 265–274.

[61] Thon MR, Nuckles EM, Takach JE, Vaillancourt LJ (2002) CPR1: A Gene Encoding a Putative Signal Peptidase That Functions in Pathogenicity of Colletotrichum graminicola to Maize. Molecular Plant-Microbe Interactions 15: 120–128. 11876424 10.1094/MPMI.2002.15.2.120

[62] Monod M, Capoccia S, Lechenne B, Zaugg C, Holdom M, et al (2002) Secreted proteases from pathogenic fungi. Int J Med Microbiol 292: 405–419. 12452286 10.1078/1438-4221-00223

[63] Dow JM, Davies HA, Daniels MJ (1998) A metalloprotease from Xanthomonas campestris that specifically degrades proline/hydroxyproline-rich glycoproteins of the plant extracellular matrix. Mol Plant Microbe Interact 11: 1085–1093. 9805395 10.1094/MPMI.1998.11.11.1085

[64] Sreedhar L, Kobayashi DY, Bunting TE, Hillman BI, Belanger FC (1999) Fungal proteinase expression in the interaction of the plant pathogen Magnaporthe poae with its host. Gene 235: 121–129. 10415340 10.1016/s0378-1119(99)00201-2

[65] Di Pietro A, Huertas-Gonzalez MD, Gutierrez-Corona JF, Martinez-Cadena G, Meglecz E, et al (2001) Molecular characterization of a subtilase from the vascular wilt fungus Fusarium oxysporum. Mol Plant Microbe Interact 14: 653–662. 11332729 10.1094/MPMI.2001.14.5.653

[66] Kolattukudy PE (1985) Enzymatic penetration of the plant cuticle by fungal pathogens. Proceedings of the National Academy of Sciences, USA 92: 4080–4087.

[67] Voigt CA, Schäfer W, Salomon S (2005) A secreted lipase of Fusarium graminearum is a virulence factor required for infection of cereals. The Plant Journal 42: 364–375. 15842622 10.1111/j.1365-313X.2005.02377.x

[68] Rogers LM, Flaishman MA, Kolattukudy PE (1994) Cutinase gene disruption in Fusarium solani f sp pisi decreases its virulence on pea. Plant Cell 6: 935–945. 8069105 10.1105/tpc.6.7.935 PMC160490

[69] Deising H, Nicholson RL, Haug M, Howard RJ, Mendgen K (1992) Adhesion Pad Formation and the Involvement of Cutinase and Esterases in the Attachment of Uredospores to the Host Cuticle. Plant Cell 4: 1101–1111. 12297669 10.1105/tpc.4.9.1101 PMC160200

[70] Pascholati SF, Deising H, Leiti B, Anderson D, Nicholson RL (1993) Cutinase and non-specific esterase activities in the conidial mucilage of Colletotrichum graminicola. Physiological and Molecular Plant Pathology 42: 37–51.

[71] Kolattukudy PE, Rogers LM, Li D, Hwang CS, Flaishman MA (1995) Surface signaling in pathogenesis. Proc Natl Acad Sci U S A 92: 4080–4087. 7753774 10.1073/pnas.92.10.4080 PMC41890

[72] Feng J, Wang F, Hughes GR, Kaminskyj S, Wei Y (2011) An important role for secreted esterase in disease establishment of the wheat powdery mildew fungus Blumeria graminis f. sp. tritici. Can J Microbiol 57: 211–216. 21358762 10.1139/W10-120

[73] De Wit PJGM, Van der Burgt A, Ökmen B, Stergiopoulos I, Aerts AL, et al (2012) The genomes of the fungal plant pathogens Cladosporium fulvum and Dothistroma septosporum reveal adaptation to different hosts and lifestyles but also signatures of common ancestry. PLoS Genetics In Press. 10.1371/journal.pgen.1003088 PMC3510045 23209441

[74] Hane JK, Oliver RP (2008) RIPCAL: a tool for alignment-based analysis of repeat-induced point mutations in fungal genomic sequences. BMC Bioinformatics 9: 478. 19014496 10.1186/1471-2105-9-478 PMC2621366

[75] Selker EU (1990) Premeiotic instability of repeated sequences in Neurospora crassa. Annu Rev Genet 24: 579–613. 2150906 10.1146/annurev.ge.24.120190.003051

[76] Margolin BS, Garrett-Engele PW, Stevens JN, Fritz DY, Garrett-Engele C, et al (1998) A methylated Neurospora 5S rRNA pseudogene contains a transposable element inactivated by repeat-induced point mutation. Genetics 149: 1787–1797. 9691037 10.1093/genetics/149.4.1787 PMC1460257

[77] Grigoriev IV, Nordberg H, Shabalov I, Aerts A, Cantor M, et al (2011) The Genome Portal of the Department of Energy Joint Genome Institute. Nucleic Acids Research 40: D26–D32. 22110030 10.1093/nar/gkr947 PMC3245080

[78] Cho Y, Srivastava A, Ohm RA, Lawrence CB, Wang KH, et al (2012) Transcription factor Amr1 induces melanin biosynthesis and suppresses virulence in Alternaria brassicicola. PLoS Pathogens In Press. 10.1371/journal.ppat.1002974 PMC3486909 23133370

[79] Jaffe DB, Butler J, Gnerre S, Mauceli E, Lindblad-Toh K, et al (2003) Whole-genome sequence assembly for mammalian genomes: Arachne 2. Genome Res 13: 91–96. 12529310 10.1101/gr.828403 PMC430950

[80] Margulies M, Egholm M, Altman WE, Attiya S, Bader JS, et al (2005) Genome sequencing in microfabricated high-density picolitre reactors. Nature 437: 376–380. 16056220 10.1038/nature03959 PMC1464427

[81] Zerbino DR, Birney E (2008) Velvet: algorithms for de novo short read assembly using de Bruijn graphs. Genome Res 18: 821–829. 18349386 10.1101/gr.074492.107 PMC2336801

[82] Stanke M, Keller O, Gunduz I, Hayes A, Waack S, et al (2006) AUGUSTUS: ab initio prediction of alternative transcripts. Nucleic Acids Res 34: W435–439. 16845043 10.1093/nar/gkl200 PMC1538822

[83] Nierman WC, Pain A, Anderson MJ, Wortman JR, Kim HS, et al (2005) Genomic sequence of the pathogenic and allergenic filamentous fungus Aspergillus fumigatus. Nature 438: 1151–1156. 16372009 10.1038/nature04332

[84] Smith AFA, Hubley R, Green P (2004) RepeatMasker, version Open-3.0. Available: http://www.repeatmasker.org. Accessed 17 October 2011.

[85] Jurka J, Kapitonov VV, Pavlicek A, Klonowski P, Kohany O, et al (2005) Repbase Update, a database of eukaryotic repetitive elements. Cytogenet Genome Res 110: 462–467. 16093699 10.1159/000084979

[86] Price AL, Jones NC, Pevzner PA (2005) De novo identification of repeat families in large genomes. Bioinformatics 21 Suppl 1: i351–358. 15961478 10.1093/bioinformatics/bti1018

[87] Salamov AA, Solovyev VV (2000) Ab initio gene finding in Drosophila genomic DNA. Genome Res 10: 516–522. 10779491 10.1101/gr.10.4.516 PMC310882

[88] Birney E, Durbin R (2000) Using GeneWise in the Drosophila Annotation Experiment. Genome Research 10: 547–548. 10779496 10.1101/gr.10.4.547 PMC310858

[89] Isono K, McIninch JD, Borodovsky M (1994) Characteristic Features of the Nucleotide Sequences of Yeast Mitochondrial Ribosomal Protein Genes as Analyzed by Computer Program GeneMark. DNA Research 1: 263–269. 7719921 10.1093/dnares/1.6.263

[90] Kent WJ (2002) BLAT—The BLAST-Like Alignment Tool. Genome Research 12: 656–664. 11932250 10.1101/gr.229202 PMC187518

[91] Altschul SF, Gish W, Miller W, Myers EW, Lipman DJ (1990) Basic local alignment search tool. J Mol Biol 215: 403–410. 2231712 10.1016/S0022-2836(05)80360-2

[92] Kanehisa M, Goto S, Kawashima S, Okuno Y, Hattori M (2004) The KEGG resource for deciphering the genome. Nucleic Acids Res 32: D277–280. 14681412 10.1093/nar/gkh063 PMC308797

[93] Koonin EV, Fedorova ND, Jackson JD, Jacobs AR, Krylov DM, et al (2004) A comprehensive evolutionary classification of proteins encoded in complete eukaryotic genomes. Genome Biol 5: R7. 14759257 10.1186/gb-2004-5-2-r7 PMC395751

[94] Bendtsen JD, Nielsen H, von Heijne G, Brunak S (2004) Improved prediction of signal peptides: SignalP 3.0. J Mol Biol 340: 783–795. 15223320 10.1016/j.jmb.2004.05.028

[95] Melen K, Krogh A, von Heijne G (2003) Reliability measures for membrane protein topology prediction algorithms. J Mol Biol 327: 735–744. 12634065 10.1016/s0022-2836(03)00182-7

[96] Zdobnov EM, Apweiler R (2001) InterProScan – an integration platform for the signature-recognition methods in InterPro. Bioinformatics 17: 847–848. 11590104 10.1093/bioinformatics/17.9.847

[97] Ashburner M, Ball CA, Blake JA, Botstein D, Butler H, et al (2000) Gene ontology: tool for the unification of biology. The Gene Ontology Consortium. Nat Genet 25: 25–29. 10802651 10.1038/75556 PMC3037419

[98] Enright AJ, Van Dongen S, Ouzounis CA (2002) An efficient algorithm for large-scale detection of protein families. Nucleic Acids Res 30: 1575–1584. 11917018 10.1093/nar/30.7.1575 PMC101833

[99] Van Dongen S (2000) Graph Clustering by Flow Simulation [PhD dissertation]. Utrecht University, The Netherlands. http://igitur-archive.library.uu.nl/dissertations/1895620/inhoud.htm.

[100] De Bie T, Cristianini N, Demuth JP, Hahn MW (2006) CAFE: a computational tool for the study of gene family evolution. Bioinformatics 22: 1269–1271. 16543274 10.1093/bioinformatics/btl097

[101] Wicker T, Sabot F, Hua-Van A, Bennetzen JL, Capy P, et al (2007) A unified classification system for eukaryotic transposable elements. Nat Rev Genet 8: 973–982. 17984973 10.1038/nrg2165

[102] Hall TA (1999) BioEdit: a user-friendly biological sequence alignment editor and analysis program for Windows 95/98/NT. Nucleic Acids Symposium Series 41: 95–98.

[103] Liu K, Raghavan S, Nelesen S, Linder CR, Warnow T (2009) Rapid and accurate large-scale coestimation of sequence alignments and phylogenetic trees. Science 324: 1561–1564. 19541996 10.1126/science.1171243

[104] Katoh K, Asimenos G, Toh H (2009) Multiple alignment of DNA sequences with MAFFT. Methods Mol Biol 537: 39–64. 19378139 10.1007/978-1-59745-251-9_3

[105] Stamatakis A (2006) RAxML-VI-HPC: maximum likelihood-based phylogenetic analyses with thousands of taxa and mixed models. Bioinformatics 22: 2688–2690. 16928733 10.1093/bioinformatics/btl446

[106] Abascal F, Zardoya R, Posada D (2005) ProtTest: selection of best-fit models of protein evolution. Bioinformatics 21: 2104–2105. 15647292 10.1093/bioinformatics/bti263

[107] Miller MA, Pfeiffer W, Schwartz T. (2010) Creating the CIPRES Science Gateway for inference of large phylogenetic trees. In: Proceedings of the Gateway Computing Environments Workshop (GCE), 14 Nov. 2010, New Orleans, Louisiana, United States of America. Available: http://www.phylo.org/sub_sections/portal/sc2010_paper.pdf. Accessed: 11 June 2011. .

[108] Stamatakis A, Hoover P, Rougemont J (2008) A rapid bootstrap algorithm for the RAxML Web servers. Syst Biol 57: 758–771. 18853362 10.1080/10635150802429642

[109] Sanderson MJ (2003) r8s: inferring absolute rates of molecular evolution and divergence times in the absence of a molecular clock. Bioinformatics 19: 301–302. 12538260 10.1093/bioinformatics/19.2.301

[110] Gueidan C, Ruibal C, de Hoog GS, Schneider H (2011) Rock-inhabiting fungi originated during periods of dry climate in the late Devonian and middle Triassic. Fungal Biol 115: 987–996. 21944211 10.1016/j.funbio.2011.04.002

[111] Drummond AJ, Rambaut A (2007) BEAST: Bayesian evolutionary analysis by sampling trees. BMC Evol Biol 7: 214. 17996036 10.1186/1471-2148-7-214 PMC2247476

[112] Taylor JW, Berbee ML (2006) Dating divergences in the Fungal Tree of Life: review and new analyses. Mycologia 98: 838–849. 17486961 10.3852/mycologia.98.6.838

[113] Katoh K, Kuma K, Toh H, Miyata T (2005) MAFFT version 5: improvement in accuracy of multiple sequence alignment. Nucleic Acids Res 33: 511–518. 15661851 10.1093/nar/gki198 PMC548345

[114] Castresana J (2000) Selection of conserved blocks from multiple alignments for their use in phylogenetic analysis. Mol Biol Evol 17: 540–552. 10742046 10.1093/oxfordjournals.molbev.a026334

[115] Huson DH, Richter DC, Rausch C, Dezulian T, Franz M, et al (2007) Dendroscope: An interactive viewer for large phylogenetic trees. BMC Bioinformatics 8: 460. 18034891 10.1186/1471-2105-8-460 PMC2216043

[116] Frazer KA, Pachter L, Poliakov A, Rubin EM, Dubchak I (2004) VISTA: computational tools for comparative genomics. Nucleic Acids Res 32: W273–279. 15215394 10.1093/nar/gkh458 PMC441596

[117] Krogh A, Larsson B, von Heijne G, Sonnhammer EL (2001) Predicting transmembrane protein topology with a hidden Markov model: application to complete genomes. J Mol Biol 305: 567–580. 11152613 10.1006/jmbi.2000.4315

[118] Medema MH, Blin K, Cimermancic P, de Jager V, Zakrzewski P, et al (2011) antiSMASH: rapid identification, annotation and analysis of secondary metabolite biosynthesis gene clusters in bacterial and fungal genome sequences. Nucleic Acids Res 39: W339–346. 21672958 10.1093/nar/gkr466 PMC3125804

[119] Galagan JE, Calvo SE, Cuomo C, Ma LJ, Wortman JR, et al (2005) Sequencing of Aspergillus nidulans and comparative analysis with A. fumigatus and A. oryzae. Nature 438: 1105–1115. 16372000 10.1038/nature04341

[120] Amselem J, Cuomo CA, van Kan JA, Viaud M, Benito EP, et al (2011) Genomic analysis of the necrotrophic fungal pathogens Sclerotinia sclerotiorum and Botrytis cinerea. PLoS Genet 7: e1002230. 21876677 10.1371/journal.pgen.1002230 PMC3158057

[121] Berka RM, Grigoriev IV, Otillar R, Salamov A, Grimwood J, et al (2011) Comparative genomic analysis of the thermophilic biomass-degrading fungi Myceliophthora thermophila and Thielavia terrestris. Nat Biotech 29: 922–927. 10.1038/nbt.1976 21964414

[122] Cuomo CA, Guldener U, Xu JR, Trail F, Turgeon BG, et al (2007) The Fusarium graminearum genome reveals a link between localized polymorphism and pathogen specialization. Science 317: 1400–1402. 17823352 10.1126/science.1143708

[123] Ma LJ, van der Does HC, Borkovich KA, Coleman JJ, Daboussi MJ, et al (2010) Comparative genomics reveals mobile pathogenicity chromosomes in Fusarium. Nature 464: 367–373. 20237561 10.1038/nature08850 PMC3048781

[124] Dean RA, Talbot NJ, Ebbole DJ, Farman ML, Mitchell TK, et al (2005) The genome sequence of the rice blast fungus Magnaporthe grisea. Nature 434: 980–986. 15846337 10.1038/nature03449

[125] Coleman JJ, Rounsley SD, Rodriguez-Carres M, Kuo A, Wasmann CC, et al (2009) The genome of Nectria haematococca: contribution of supernumerary chromosomes to gene expansion. PLoS Genet 5: e1000618. 19714214 10.1371/journal.pgen.1000618 PMC2725324

[126] Galagan JE, Calvo SE, Borkovich KA, Selker EU, Read ND, et al (2003) The genome sequence of the filamentous fungus Neurospora crassa. Nature 422: 859–868. 12712197 10.1038/nature01554

[127] Goffeau A, Barrell G, Bussey H, Davis RW, Dujon B, et al (1996) Life with 6000 genes. Science 274: 546–567. 8849441 10.1126/science.274.5287.546

[128] Martinez D, Berka RM, Henrissat B, Saloheimo M, Arvas M, et al (2008) Genome sequencing and analysis of the biomass-degrading fungus Trichoderma reesei (syn. Hypocrea jecorina). Nat Biotechnol 26: 553–560. 18454138 10.1038/nbt1403

[129] Klosterman SJ, Subbarao KV, Kang S, Veronese P, Gold SE, et al (2011) Comparative genomics yields insights into niche adaptation of plant vascular wilt pathogens. PLoS Pathog 7: e1002137. 21829347 10.1371/journal.ppat.1002137 PMC3145793

[130] Stajich JE, Wilke SK, Ahrén D, Au CH, Birren BW, et al (2010) Insights into evolution of multicellular fungi from the assembled chromosomes of the mushroom Coprinopsis cinerea (Coprinus cinereus). Proceedings of the National Academy of Sciences of the United States of America 107: 11889–11894. 20547848 10.1073/pnas.1003391107 PMC2900686

[131] Loftus BJ, Fung E, Roncaglia P, Rowley D, Amedeo P, et al (2005) The genome of the basidiomycetous yeast and human pathogen Cryptococcus neoformans. Science 307: 1321–1324. 15653466 10.1126/science.1103773 PMC3520129

[132] Duplessis S, Cuomo CA, Lin YC, Aerts A, Tisserant E, et al (2011) Obligate biotrophy features unraveled by the genomic analysis of rust fungi. Proc Natl Acad Sci U S A 108: 9166–9171. 21536894 10.1073/pnas.1019315108 PMC3107277

[133] Martinez D, Larrondo LF, Putnam N, Gelpke MD, Huang K, et al (2004) Genome sequence of the lignocellulose degrading fungus Phanerochaete chrysosporium strain RP78. Nat Biotechnol 22: 695–700. 15122302 10.1038/nbt967

[134] Martinez D, Challacombe J, Morgenstern I, Hibbett D, Schmoll M, et al (2009) Genome, transcriptome, and secretome analysis of wood decay fungus Postia placenta supports unique mechanisms of lignocellulose conversion. Proc Natl Acad Sci U S A 106: 1954–1959. 19193860 10.1073/pnas.0809575106 PMC2644145

[135] Ohm RA, De Jong JF, Lugones LG, Aerts A, Kothe E, et al (2010) Genome sequence of the model mushroom Schizophyllum commune. Nature Biotechnology 28: 957–963. 10.1038/nbt.1643 20622885

[136] Kamper J, Kahmann R, Bolker M, Ma LJ, Brefort T, et al (2006) Insights from the genome of the biotrophic fungal plant pathogen Ustilago maydis. Nature 444: 97–101. 17080091 10.1038/nature05248

