# Correction: Diverse Lifestyles and Strategies of Plant Pathogenesis Encoded in the Genomes of Eighteen *Dothideomycetes* Fungi

**DOI:** 10.1371/annotation/fcca88ac-d684-46e0-a483-62af67e777bd

**Published:** 2013-03-05

**Authors:** Robin A. Ohm, Nicolas Feau, Bernard Henrissat, Conrad L. Schoch, Benjamin A. Horwitz, Kerrie W. Barry, Bradford J. Condon, Alex C. Copeland, Braham Dhillon, Fabian Glaser, Cedar N. Hesse, Idit Kosti, Kurt LaButti, Erika A. Lindquist, Susan Lucas, Asaf A. Salamov, Rosie E. Bradshaw, Lynda Ciuffetti, Richard C. Hamelin, Gert H. J. Kema, Christopher Lawrence, James A. Scott, Joseph W. Spatafora, B. Gillian Turgeon, Pierre J. G. M. de Wit, Shaobin Zhong, Stephen B. Goodwin, Igor V. Grigoriev

There were errors in the Funding statement. The correct funding information is as follows: "The work done by U.S. Department of Energy Joint Genome Institute is supported by the Office of Science of the U.S. Department of Energy under contract number DE-AC02-05CH11231. This work was supported in part by USDA-ARS CRIS project 3602-22000-015-00D and by Genome Canada and Genome BC LSARPC project 2112. CLS acknowledges support from the Intramural Research Program of the NIH, National Library of Medicine. The funders had no role in study design, data collection and analysis, decision to publish, or preparation of the manuscript."

