# Supplementary figures and images for: Diverse Lifestyles and Strategies of Plant Pathogenesis Encoded in the Genomes of Eighteen Dothideomycetes Fungi
Source: PLoS Pathog. 2012 Dec 6;8(12):e1003037. doi: 10.1371/journal.ppat.1003037 (PMC3516569; doi:10.1371/journal.ppat.1003037)

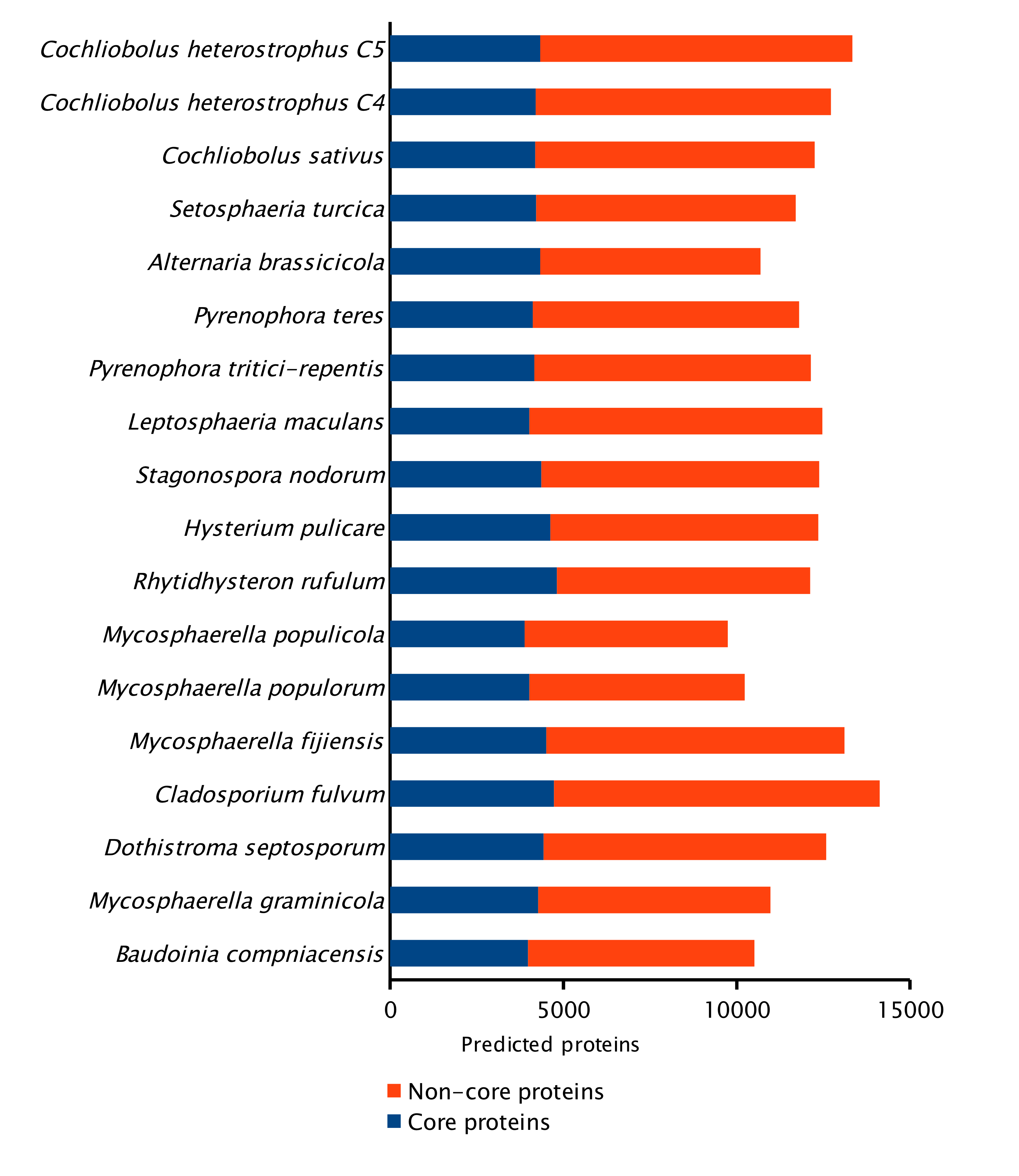

Supplement: Figure S1 — Ratio of predicted core and non-core proteins in the 18 genomes of Dothideomycetes . (TIFF) [file ppat.1003037.s001.tiff]

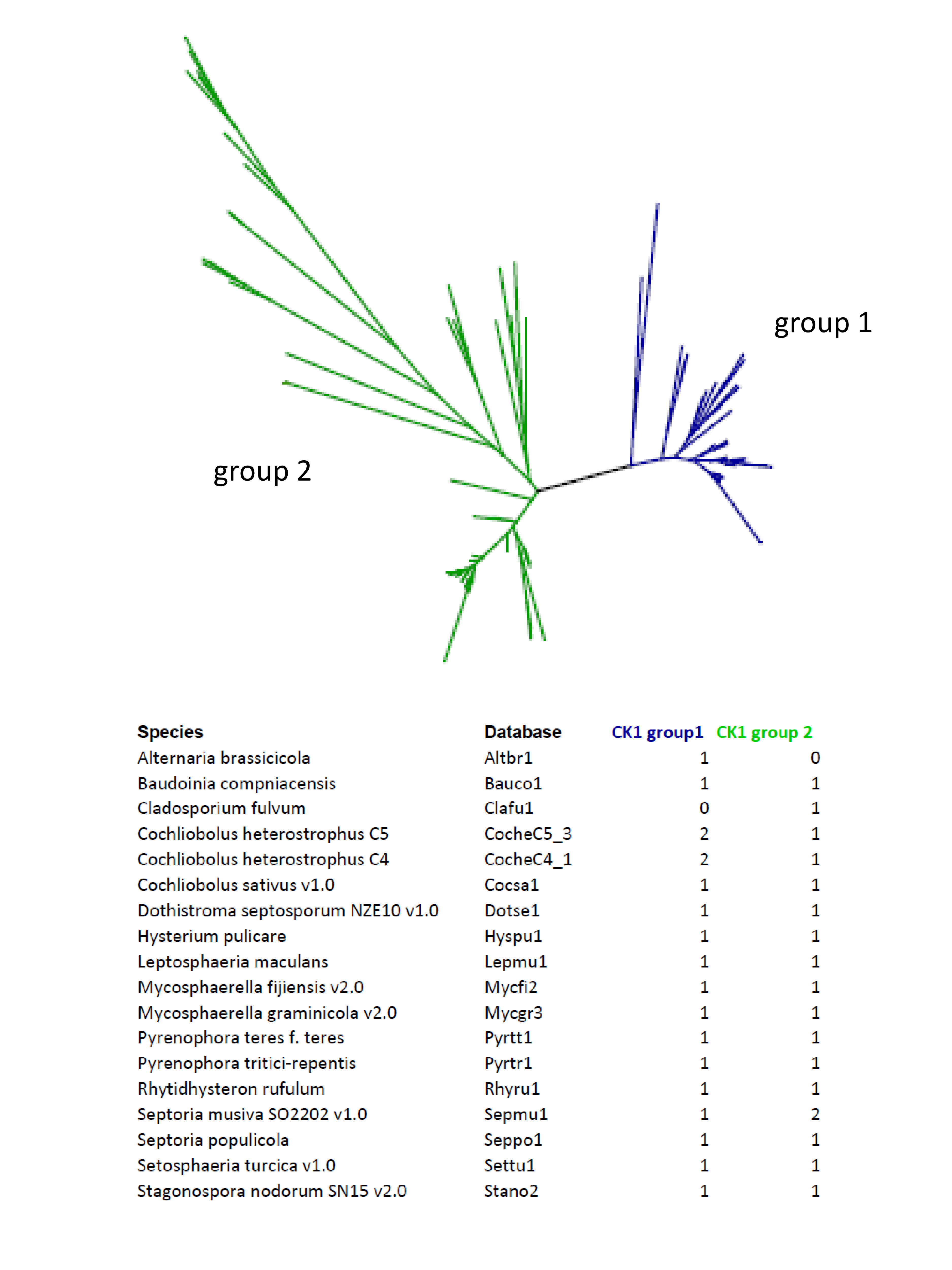

Supplement: Figure S2 — Phylogeny and gene count of predicted casein kinase 1 (CK1) genes in the genomes of 18 Dothideomycetes . (TIFF) [file ppat.1003037.s002.tiff]

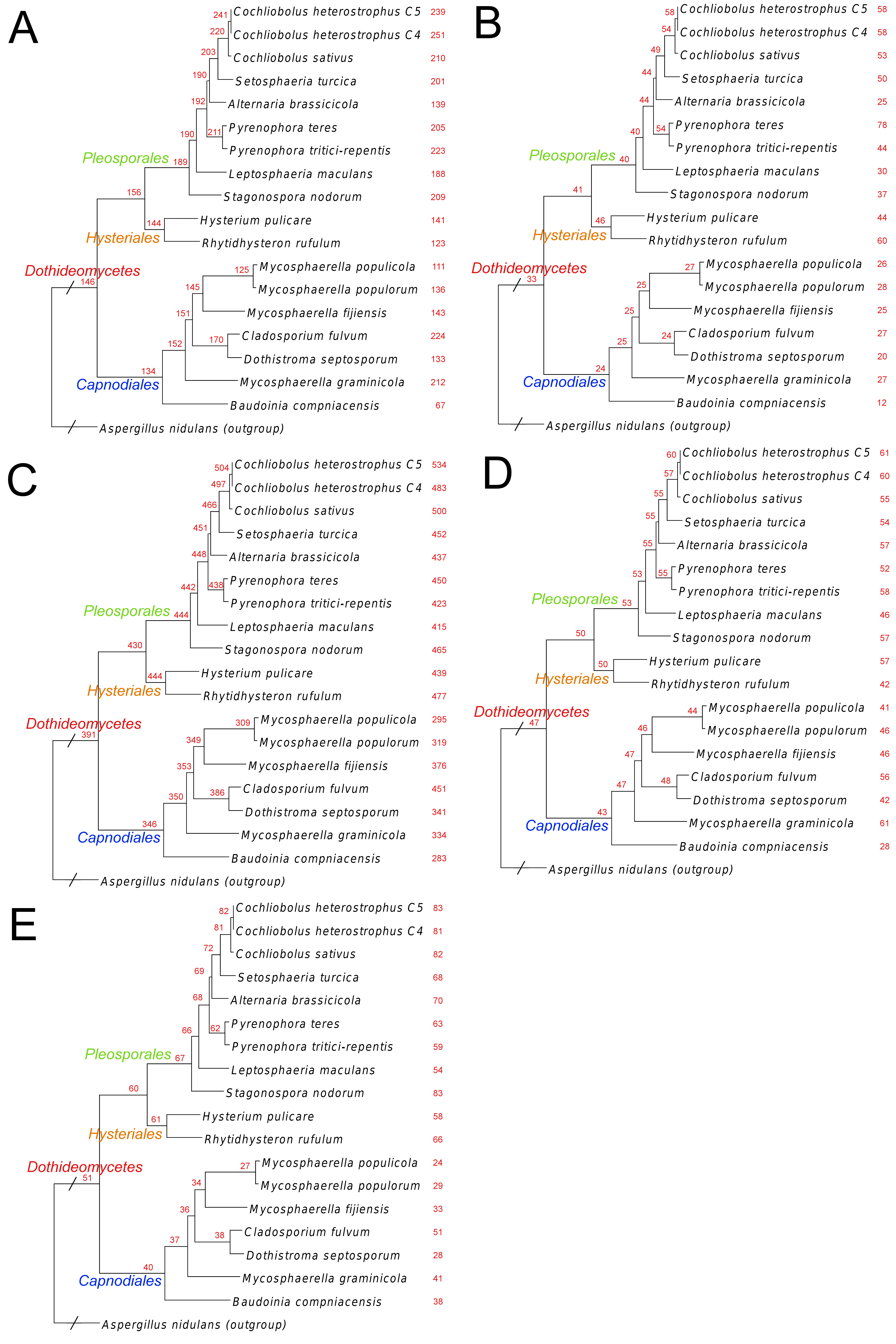

Supplement: Figure S3 — Gene counts of classes that have been implicated in plant pathogenesis. Gene counts in extant species (indicated right of the tree) and estimated gene counts in inferred last common ancestors (indicated on the tree nodes) are given. A. Genes encoding small secreted proteins. B. Genes encoding proteins involved in secondary metabolite production. C. Genes encoding carbohydrate-active enzymes (CAZymes). D. Genes encoding secreted peptidases. E. Genes encoding secreted lipases. (TIFF) [file ppat.1003037.s003.tiff]

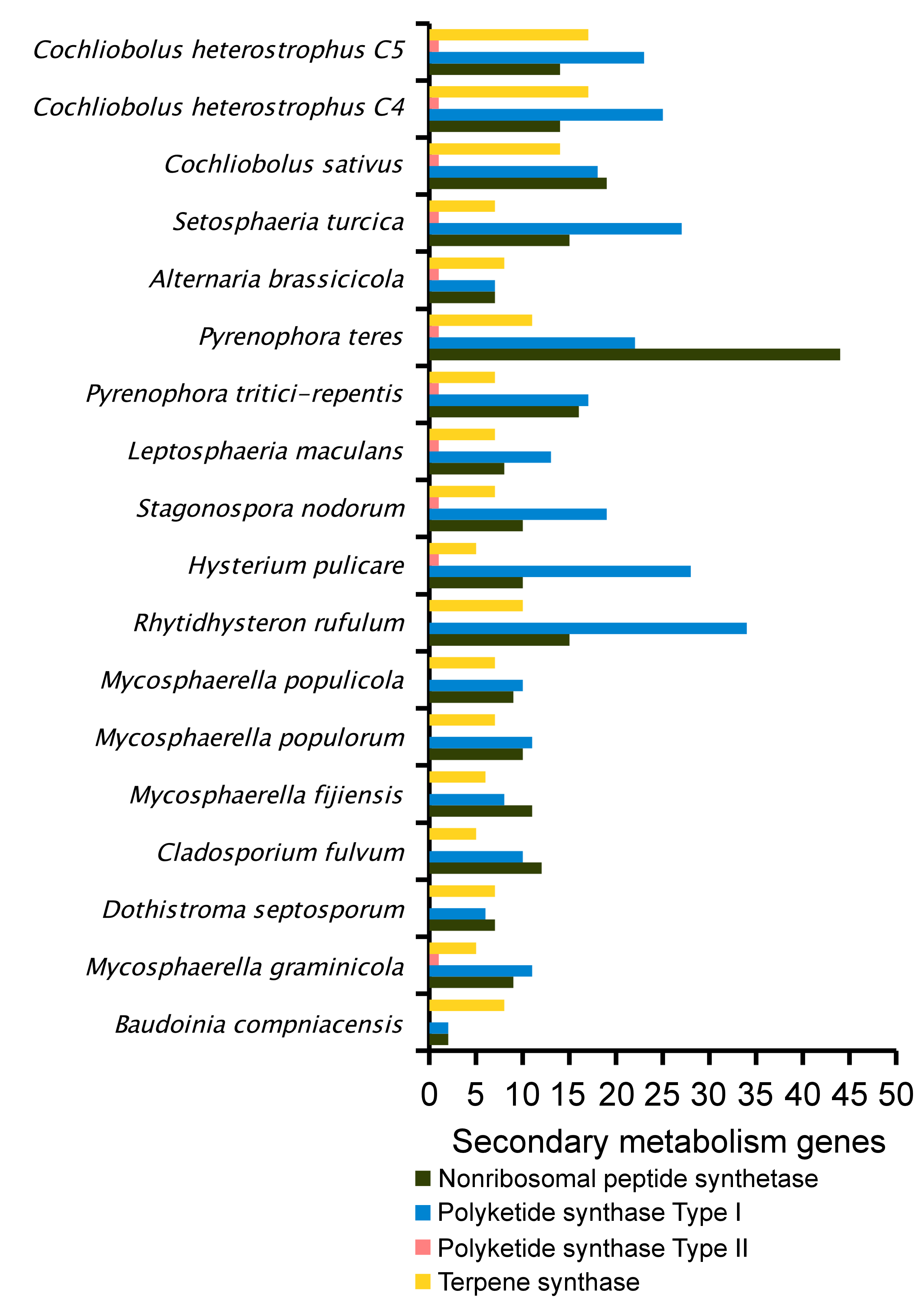

Supplement: Figure S4 — Predicted genes involved in secondary metabolism. Genomes of organisms belonging to the Pleosporales and Hysteriales generally contain more genes involved in this process than the Capnodiales, especially in the case of Polyketide synthase Type I. (TIFF) [file ppat.1003037.s004.tiff]

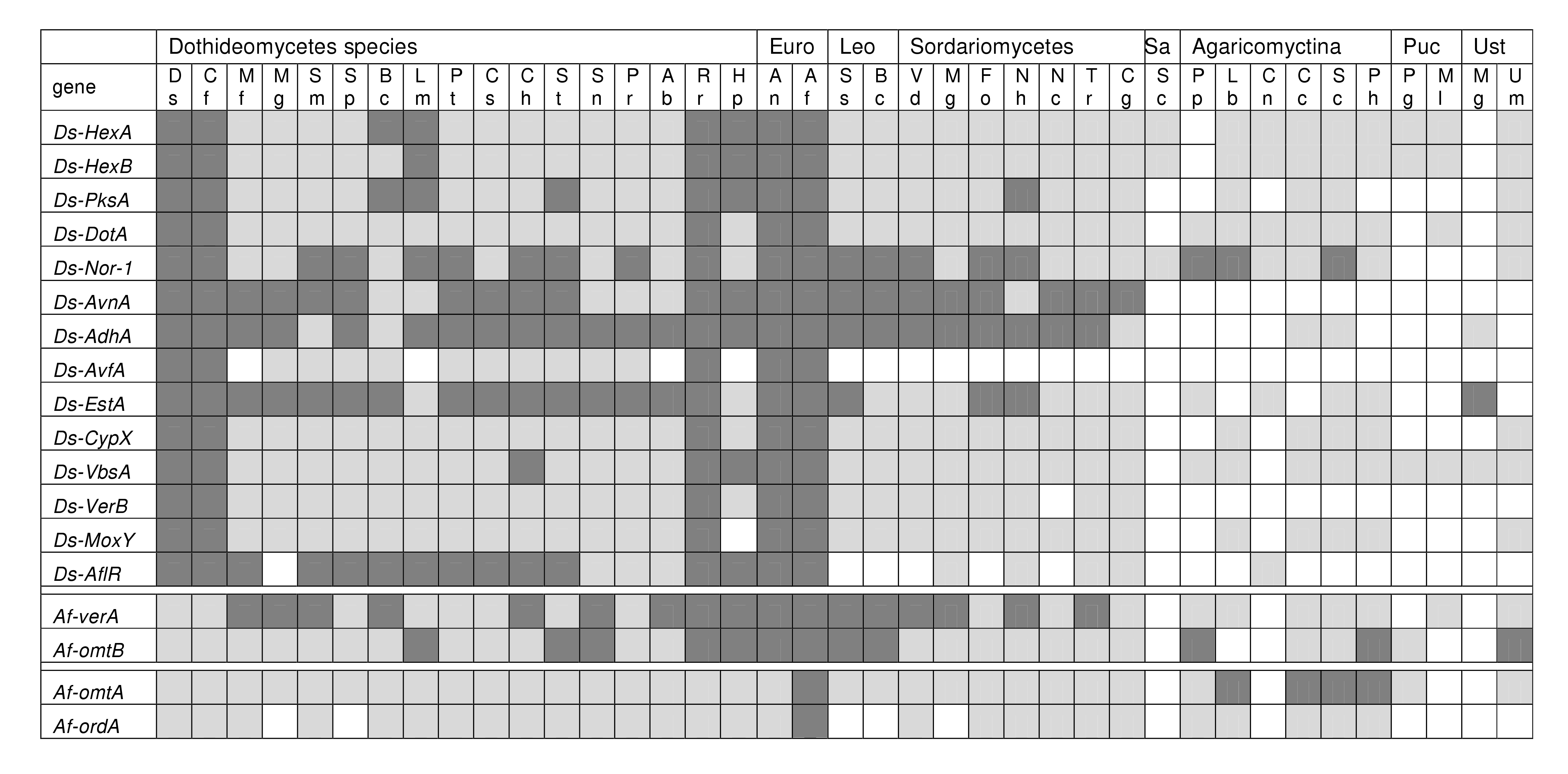

Supplement: Figure S5 — Presence of aflatoxin-like genes in Dothideomycetes and other fungi. Aflatoxin-like genes were found using BLASTP (e-value of 10−5) of predicted amino acid sequences of Dothistroma septosporum dothistromin genes (main block of 14 Ds genes) or Aspergillus flavus sterigmatocystin (verA [aflN] and omtB [aflO]) or aflatoxin genes (omtA [aflP], ordA [aflQ]) (Yu et al 2004), against gene catalogues for all genomes shown, with reciprocal BLAST back to the D. septosporum or A. flavus genomes. Dark gray boxes indicate presence of a reciprocal best hit, light gray indicates one-directional best hit, and white indicates no hit. Dothideomycetes are D. septosporum (Ds), Cladosporium fulvum (Cf), Mycosphaerella fijiensis (Mf), Mycosphaerella graminicola (Mg), Septoria musiva (Sm), Septoria populicola (Sp), Baudoinia compniacensis (Bc), Leptosphaeria maculans (Lm), Pyrenophora teres f. teres (Pt), Cochliobolus sativus (Cs), Cochliobolus heterostrophus (Ch), Setosphaeria turcica (St), Stagonospora nodorum (Sn), Pyrenophora tritici-repentis (Pr), Alternaria brassicicola (Ab), Rhytidhysteron rufulum (Rr), Hysterium pulicare (Hp). Eurotiomycetes (Euro) are Aspergillus nidulans (An), A. flavus (Af). Leotiomycetes (Leo) are Sclerotinia sclerotiorum (Ss), Botrytis cinerea (Bc). Sordariomycetes (Sord) are Verticillium dahliae (Vd), Magnaporthe grisea (Mg), Fusarium oxysporum (Fo), Nectria haematococca (Nh), Neurospora crassa (Nc), Trichoderma reesei (Tr), Chaetomium globosum (Cg). Saccharomycotina (S) is Saccharomyces cerevisiae (Sc). Basidiomycete outgroups are the Agaricomycotina (Agar) Postia placenta (Pp), Laccaria bicolor (Lb), Cryptococcus neoformans var. grubii (Cn), Coprinopsis cinerea (Cc), Schizophyllum commune (Sc), Phanerochaete chrysosporium (Ph); Pucciniomycotina (Puc) Puccinia graminis (Pg), Melampsora laricis-populina (Ml); Ustilaginomycotina (Ust) Malassezia globosa (Mg), Ustilago maydis (Um). Protein ID numbers of query genes are 66976, 181128, 192192, 192193, 75691, 573 [file ppat.1003037.s005.tiff]

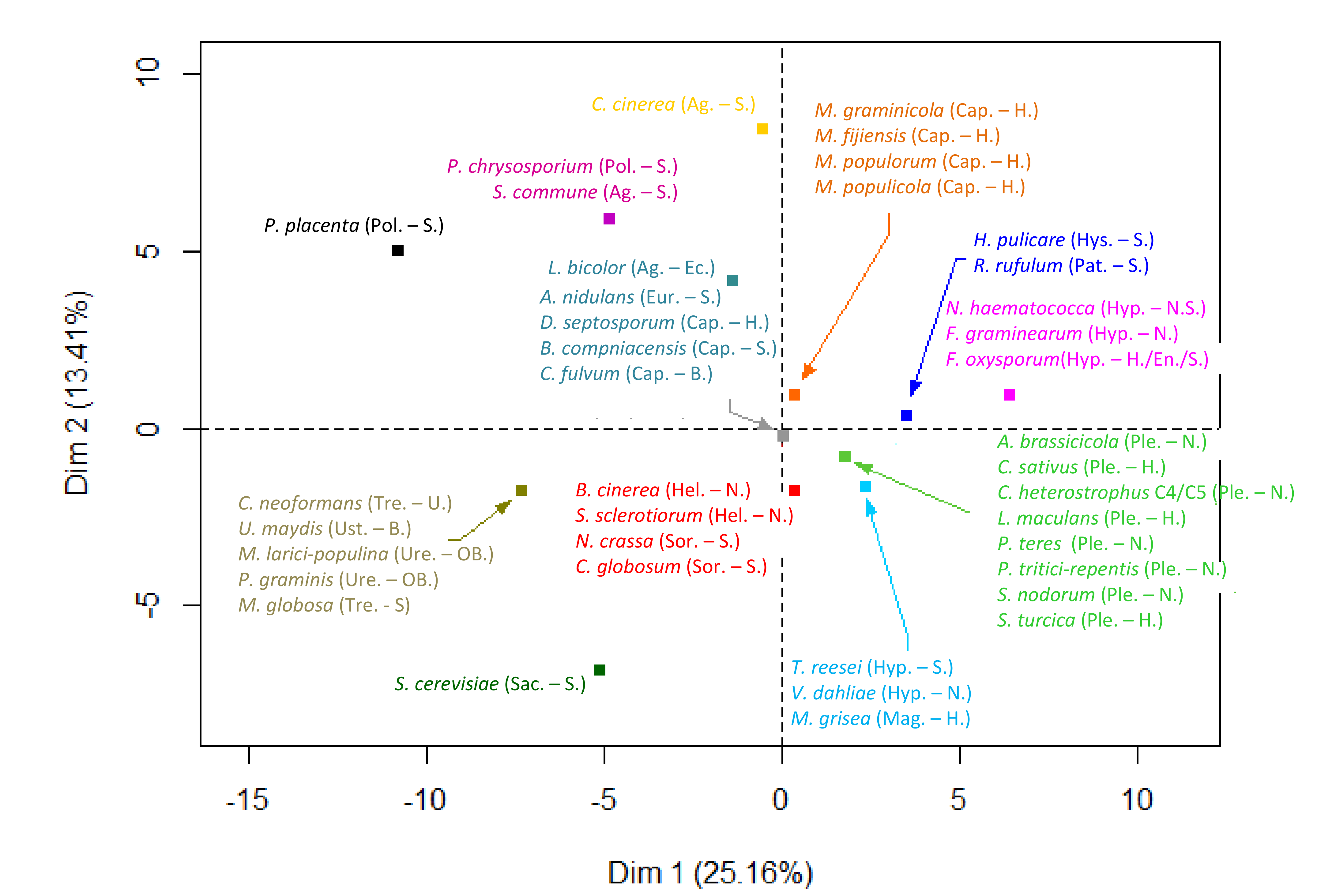

Supplement: Figure S6 — Principal component analysis and hierarchical clustering of protease family assignments of 18 Dothideomycetes and outgroups. A matrix was constructed containing the number of proteases assigned to each MEROPS family in each fungal species. Analyses were performed with the Rcmdr package of R, and the results graphed along the first two components. Colored squares represent the centroid of each cluster of species, indicated with the same color. Taxonomy: Ag., Agaricales; Cap., Capnodiales; Dia., Diaporthales; Hel., Helotiales; Hyp., Hypocreales; Hys., Hysteriales; Mag., Magnaporthales; Mal., Malasseziales; Pat., Patellariales; Ple., Pleosporales; Pol. Polyporales; Sac., Saccharomycetales; Ure., Uredinales; Ust., Ustilaginales; Tre., Tremellales. Lifestyles: B., Biotroph; Ec., Ectomycorrhizal; En., Endophyte; OB., Obligate biotroph; S., Saprotroph; N., Necrotroph; H., Hemi-biotroph; U., Undetermined. (TIFF) [file ppat.1003037.s006.tiff]

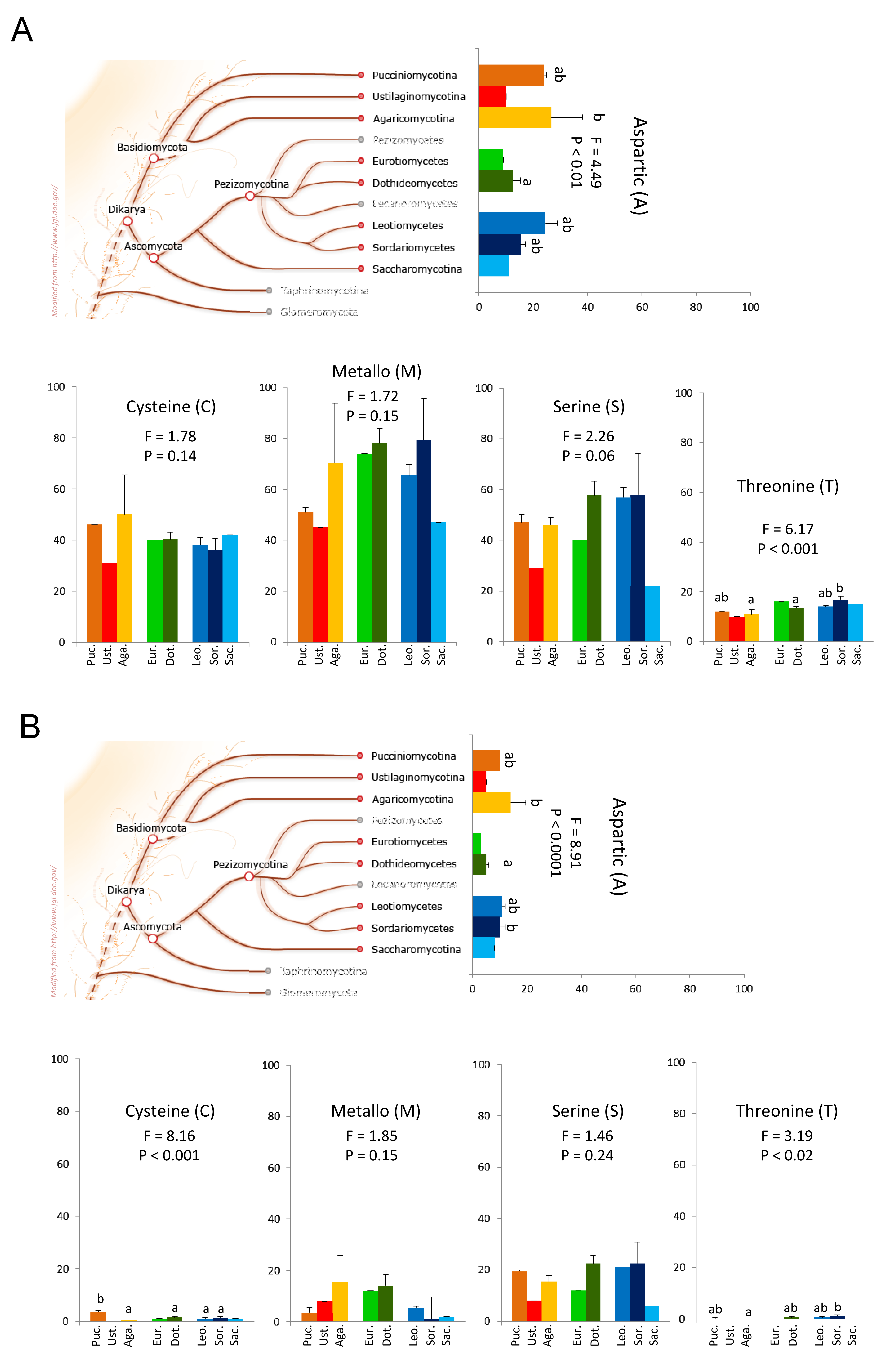

Supplement: Figure S7 — Distribution of peptidase families in Dothideomycetes and other fungal classes. Putative peptidases were classified based on the MEROPS database. A. Non-secreted and secreted peptidases. B. Secreted peptidases. Letters above columns indicate significant differences between peptidase content means as determined by a Tukey's HSD test after significant one-way ANOVA. (TIFF) [file ppat.1003037.s007.tiff]

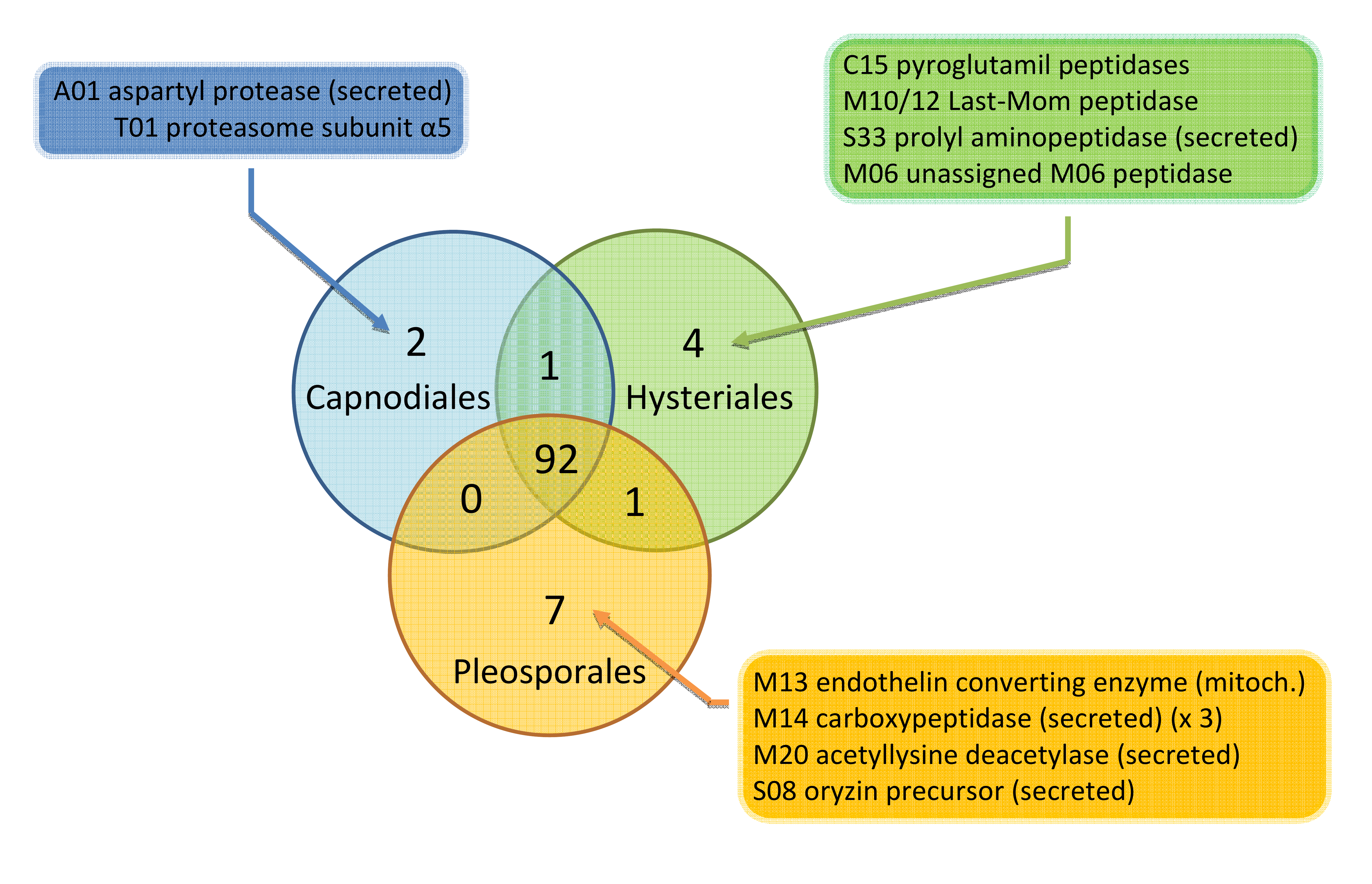

Supplement: Figure S8 — Number of genes encoding peptidases in the genomes of Capnodiales , Pleosporales and Hysteriales fungi. (TIFF) [file ppat.1003037.s008.tiff]

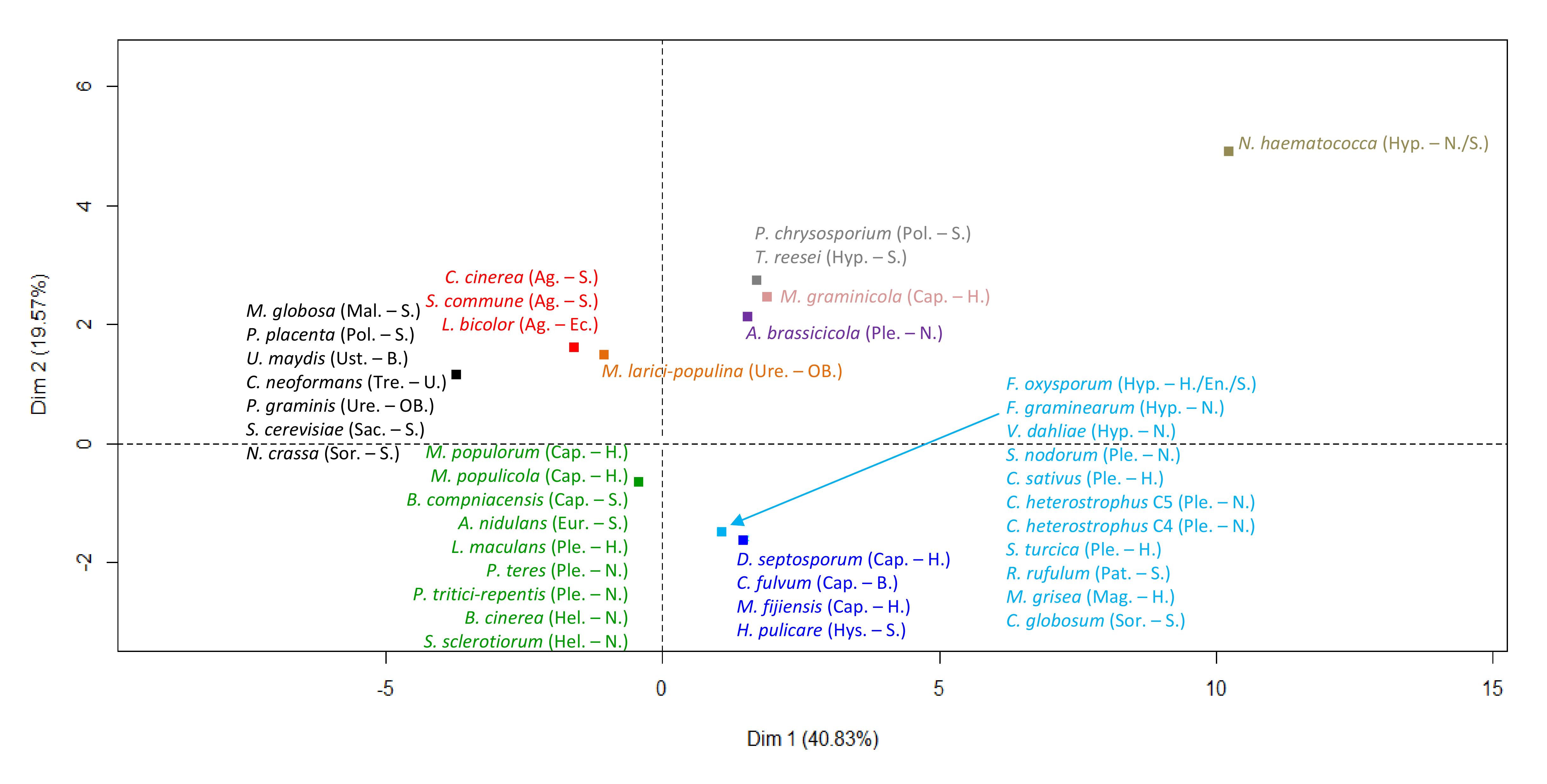

Supplement: Figure S9 — Principal component analysis and hierarchical clustering of lipase family assignments of 18 Dothideomycetes and outgroups. Colored squares represent the centroid of each cluster of species, indicated with the same color. Taxonomy: Ag., Agaricales; Cap., Capnodiales; Dia., Diaporthales; Hel., Helotiales; Hyp., Hypocreales; Hys., Hysteriales; Mag., Magnaporthales; Mal., Malasseziales; Pat., Patellariales; Ple., Pleosporales; Pol. Polyporales; Sac., Saccharomycetales; Ure., Uredinales; Ust., Ustilaginales; Tre., Tremellales. Lifestyles: B., Biotroph; Ec., Ectomycorrhizal; En., Endophyte; OB., Obligate biotroph; S., Saprotroph; N., Necrotroph; H., Hemi-biotroph; U., Undetermined. (TIFF) [file ppat.1003037.s009.tiff]
