# Supplementary material for: Diverse Lifestyles and Strategies of Plant Pathogenesis Encoded in the Genomes of Eighteen Dothideomycetes Fungi
Source: PLoS Pathog. 2012 Dec 6;8(12):e1003037. doi: 10.1371/journal.ppat.1003037 (PMC3516569; doi:10.1371/journal.ppat.1003037)
Supplement: Text S1 — Additional considerations concerning Dothideomycetes phylogeny and divergence time estimates. (DOC) [file ppat.1003037.s039.doc]

**Additional consideration concerning *Dothideomycetes* phylogeny and divergence time estimates**

The phylogenetic tree shown in Figure 1 represents 11 of 12 currently accepted orders in the class. *Acrospermales*, a small order consisting of a single family and 2 genera was omitted due to lack of sufficient protein-coding gene data in GenBank. A small number of nodes, recovered in previous phylogenies are not well supported in Figure 1. One example is *Myriangiales* which could not be recovered as monophyletic. It should be noted that Figure 1 represents only an approximation of the described genetic diversity in *Dothideomycetes*. For instance, our analysis focused on plant-pathogenic families and lineages with data available. In fact, only 5 of 26 currently accepted *Pleosporales* families are represented . The determination of divergence times in Figure 1 did not incorporate phylogenetic uncertainty and relied on a single calibration point taken from a previous analysis . Any analysis of fungal divergence times suffers from a lack of enough reliable calibration points, as well preserved fungal fossils are exceedingly rare . In spite of this these hypothetical age ranges remain a useful way to compare various lineages for equivalency in divergence.

The multiple names used for the genome-sampled species in Figure 1 reflect recent changes in concepts of these species. DNA-based phylogenies have now shown that many fungal taxa were initially circumscribed based on convergent morphological characters. As is evident in Figure 1, large and diverse genera such as *Cladosporium* do not reflect single entities. In addition to this, recently proposed changes in fungal nomenclature will result in a break from the practice of using separate names for the sexual and asexual states . Name stability is an important consideration and in several cases the most commonly applied names will be selected to represent a genus or species. This is a process that will commence over the next year and conclude over a longer period.

The divergence time estimates in Figure 1 provide a reminder that equivalent taxa in the current hierarchical system such as orders, families and genera often present very different ranges of evolutionary diversity, posing a challenge to the way comparative studies are often considered.

The phylogeny in Figure 1 provides good support for another characteristic commonly noted in phylogenies of the class*. Pleosporales* and *Capnodiales* with their sibling orders form part of two main lineages (labeled as suborders) diversifying at roughly similar times in the life history of Dothideomycetes. These groups often correlate with a clear morphological marker - the absence (*Dothideomycetidae*) and presence (*Pleosporomycetidae*) of pseudoparaphyses. Pseudoparaphyses are structural hyphae that develop in a specific way between the sexual spore-bearing asci. Some ecological differences between *Pleosporomycetidae* and *Dothideomycetidae* are also evident. For example, the majority of currently analyzed aquatic lineages are found in *Pleosporomycetidae* . This group also includes the only reported lineage of mycorrhizal species in *Dothideomycetes*, *Cenococcum* . *Dothideomycetidae*, on the other hand, contain most of the extremophile *Dothideomycetes* sequenced to date .

The placement of a number of additional lineages, with bootstrap values for relevant nodes below 70%, remain unresolved in Figure 1. These include *Botryosphaeriales*, which contain numerous important plant pathogens, often on woody plants and a lineage of rock-inhabiting fungi that includes *Coniosporium apollinis* . The presence of another lineage suggests a third major diversification event in the class centered on the plant-pathogenic species in *Venturia* within the newly proposed order *Venturiales* . Many potentially related species in this group remain unsampled with DNA. Another poorly resolved group is the lichenized *Dothideomycetes.* These are represented by the order *Trypetheliales* in Figure 1 but a few additional lineages are not included . The presented phylogeny leaves the possibility of a lichenized ancestor for *Dothideomycetes* still unresolved.

1. Zhang Y, Crous P, Schoch C, Hyde K (2011) Pleosporales. Fungal Diversity: 1-221.

2. Gueidan C, Ruibal C, de Hoog GS, Schneider H (2011) Rock-inhabiting fungi originated during periods of dry climate in the late Devonian and middle Triassic. Fungal Biol 115: 987-996.

3. Berbee ML, Taylor JW (2010) Dating the molecular clock in fungi – how close are we? Fungal Biology Reviews 24: 1-16.

4. Hawksworth Dl (2011) A new dawn for the naming of fungi: impacts of decisions made in Melbourne in July 2011 on the future publication and regulation of fungal names. IMA Fungus 2: 155-162.

5. Shearer CA, Raja HA, Miller AN, Nelson P, Tanaka K, et al. (2009) The molecular phylogeny of freshwater Dothideomycetes. Stud Mycol 64: 145-153S144.

6. Suetrong S, Schoch CL, Spatafora JW, Kohlmeyer J, Volkmann-Kohlmeyer B, et al. (2009) Molecular systematics of the marine Dothideomycetes. Stud Mycol 64: 155-173S156.

7. Spatafora J, Owensby C, Douhan G, Boehm E, Schoch C (2011) Phylogenetic placement of Cenococcum in Gloniaceae (Dothideomycetes). Mycologia (in press).

8. Ruibal C, Gueidan C, Selbmann L, Gorbushina AA, Crous PW, et al. (2009) Phylogeny of rock-inhabiting fungi related to Dothideomycetes. Stud Mycol 64: 123-133S127.

9. Sterflinger K, De Baere R, de Hoog GS, De Wachter R, Krumbein WE, et al. (1997) Coniosporium perforans and C. apollinis, two new rock-inhabiting fungi isolated from marble in the Sanctuary of Delos (Cyclades, Greece). Antonie Van Leeuwenhoek 72: 349-363.

10. Zhang Y, Crous P, Schoch C, Bahkali A, Guo L, et al. (2011) A molecular, morphological and ecological re-appraisal of Venturiales―a new order of Dothideomycetes. Fungal Diversity 51: 249-277.

11. Nelsen M, Lücking R, Mbatchou J, Andrew C, Spielmann A, et al. (2011) New insights into relationships of lichen-forming Dothideomycetes. Fungal Diversity 51: 155-162.
