# Supplementary material for: Diverse Lifestyles and Strategies of Plant Pathogenesis Encoded in the Genomes of Eighteen Dothideomycetes Fungi
Source: PLoS Pathog. 2012 Dec 6;8(12):e1003037. doi: 10.1371/journal.ppat.1003037 (PMC3516569; doi:10.1371/journal.ppat.1003037)
Supplement: Text S2 — Predicted kinases in the genomes of 18 Dothideomycetes and in those of the outgroups used. (DOC) [file ppat.1003037.s040.doc]

**Kinases**

Protein phosphorylation is central to regulation and signaling. Protein kinases and phosphatases control basic cellular functions such as cell division, as well as the response to cues originating outside the cell. Protein kinase genes have been studied in filamentous fungi by targeted gene deletion and have central roles in development and virulence .

The genomes were screened for protein kinases using a previously described pipeline that was developed based on the Kinomer HMM procedure . The resulting trees were plotted using Dendroscope .

The overall kinase counts are given in Table S16, and are similar across *Dothideomycetes*. However, in the CK1 class (casein kinase 1) variation among *Dothideomycetes* is apparent (Figure S2). Although most of the species have one representative in each of the two CK1 subgroups (color coded blue and green in Figure S2), some species have only a single CK1 (*A. brassicicola* and *C. fulvum*), or two genes belonging to one group, and one to the other group (*C. heterostrophus* and *M. populorum*). CK1 has multiple functions in mammalian cells including in the circadian clock . Together with CK2, CK1 is responsible for FRQ-dependent phosphorylation of the white-collar complex in the *Neurospora* circadian clock .

Even when the same numbers of genes are present, however, the signaling networks may be “wired” differently. Such plasticity is illustrated by a comparison of the roles of the two protein kinase A (PKA) catalytic subunit genes of *Cryptococcus neoformans* . If a particular kinase gene is absent or duplicated, one might expect even greater consequences for the phenotype. Gain, loss or modification of protein kinase genes could have far-reaching effects on fitness in a particular niche or host. Thus, it will be worthwhile to validate each case of species-specific variation, and look for biological significance. A promising class might be CK1, because this is a small kinase group, with apparent variation at the genome level between *Dothideomycete* species.

1. Zhao X, Mehrabi R, Xu JR (2007) Mitogen-activated protein kinase pathways and fungal pathogenesis. Eukaryotic cell 6: 1701-1714.

2. Kosti I, Mandel-Gutfreund Y, Glaser F, Horwitz BA (2010) Comparative analysis of fungal protein kinases and associated domains. BMC Genomics 11: 133.

3. Martin DM, Miranda-Saavedra D, Barton GJ (2009) Kinomer v. 1.0: a database of systematically classified eukaryotic protein kinases. Nucleic Acids Res 37: D244-250.

4. Huson DH, Richter DC, Rausch C, Dezulian T, Franz M, et al. (2007) Dendroscope: An interactive viewer for large phylogenetic trees. BMC Bioinformatics 8: 460.

5. Cheong JK, Virshup DM (2011) Casein kinase 1: Complexity in the family. The international journal of biochemistry & cell biology 43: 465-469.

6. He Q, Cha J, Lee HC, Yang Y, Liu Y (2006) CKI and CKII mediate the FREQUENCY-dependent phosphorylation of the WHITE COLLAR complex to close the Neurospora circadian negative feedback loop. Genes & development 20: 2552-2565.

7. Hicks JK, Heitman J (2007) Divergence of protein kinase A catalytic subunits in Cryptococcus neoformans and Cryptococcus gattii illustrates evolutionary reconfiguration of a signaling cascade. Eukaryotic cell 6: 413-420.
