# Supplementary material for: Diverse Lifestyles and Strategies of Plant Pathogenesis Encoded in the Genomes of Eighteen Dothideomycetes Fungi
Source: PLoS Pathog. 2012 Dec 6;8(12):e1003037. doi: 10.1371/journal.ppat.1003037 (PMC3516569; doi:10.1371/journal.ppat.1003037)
Supplement: Text S3 — Comparative transcriptomics during pathogenesis. (DOC) [file ppat.1003037.s041.doc]

**Comparative transcriptomics during pathogenesis**

Few whole-genome expression analyses during pathogenesis have been reported for the *Dothideomycetes*. Exceptions are fungal-plant interaction studies with *M. graminicola* and *L. maculans* and their respective hosts, wheat and *Brassica* species . Here, we compared changes in gene expression from relatively early infection (7 days for *L. maculans* and 9 days for *M. graminicola*) to relatively late infection (14 days for both species) for orthologous pairs of genes from these two fungi. There were 98 orthologous pairs for which genes in both organisms were at least two-fold up-regulated and 10 orthologous pairs of genes that were down-regulated from early to late infection (Table S6). Functional annotation terms that were over-represented in up-regulated genes included predominantly enzymes with oxidoreductase activity (Table S7). These enzymes may be involved in the defense against oxidative stress caused by hydrogen peroxide and other reactive oxygen species, which are produced by the fungus, the host plant, or both. This phenomenon has been shown previously in *M. graminicola* and we here observe a similar process in *L. maculans*, at least on the level of gene expression. There were no over-represented functional annotation terms in down-regulated genes. Interestingly, there were three putative transcription factor genes (containing a fungal-specific, a C2H2 zinc finger, and a CP2 transcription factor domain, respectively) that were strongly up-regulated in both organisms. These transcription factors may be involved in regulating the pathogenesis process and are interesting targets for further analysis. This approach of comparative transcriptomics between organisms has resulted in new leads to study the pathogenesis process of these two *Dothideomycetes*. In future experiments, a broader approach involving more species and additional comparable stages of the pathogenesis process may very well reveal more conserved gene responses, leading to new target genes for further study. This is particularly interesting in pathogen-host combinations for which the host also has been sequenced (e.g., maize , poplar and *Brassica oleracea* ), since this allows simultaneous analysis of pathogen and host gene-expression responses.

**Material and Methods**

Microarray gene-expression analyses using EST unisequences were previously reported for *Mycosphaerella graminicola* . The EST unisequences were aligned to the genome of this organism using BLAT and those overlapping with a predicted gene model were considered to originate from this gene model. If an EST unisequence overlapped with more than one gene model, the gene model with the largest overlap was chosen. Using this approach, 2571 of the 2816 unisequences (91.3%) could be mapped to a gene model and 2245 of the 10971 gene models (20.5%) had at least one unisequence mapped to it. The ratio of gene expression 14 days after inoculation and 9 days after inoculation was calculated for each gene for which expression data was available. Microarray data were also available from *Leptosphaeria maculans* . In this organism, the ratio of gene expression 14 days after inoculation and 7 days after inoculation were calculated for each gene with expression data. A bidirectional best-hit approach was taken to identify putative orthologs between *M. graminicola* and *L. maculans*. An all-versus-all BLASTp analysis was performed using an E-value cutoff of 1e-10. In case of a bidirectional best hit between two sequences of the two organisms, these sequences were considered to be putative orthologs. Orthologous pairs were identified for which the change in expression was at least two-fold up in both organisms or at least two-fold down in both organisms.

**References**

1. Keon J, Antoniw J, Carzaniga R, Deller S, Ward JL, et al. (2007) Transcriptional adaptation of Mycosphaerella graminicola to programmed cell death (PCD) of its susceptible wheat host. Mol Plant Microbe Interact 20: 178-193.

2. Rouxel T, Grandaubert J, Hane JK, Hoede C, van de Wouw AP, et al. (2011) Effector diversification within compartments of the Leptosphaeria maculans genome affected by Repeat-Induced Point mutations. Nat Commun 2: 202.

3. Shetty NP, Mehrabi R, Lutken H, Haldrup A, Kema GH, et al. (2007) Role of hydrogen peroxide during the interaction between the hemibiotrophic fungal pathogen Septoria tritici and wheat. New Phytol 174: 637-647.

4. Schnable PS, Ware D, Fulton RS, Stein JC, Wei F, et al. (2009) The B73 maize genome: complexity, diversity, and dynamics. Science 326: 1112-1115.

5. Tuskan GA, Difazio S, Jansson S, Bohlmann J, Grigoriev I, et al. (2006) The genome of black cottonwood, Populus trichocarpa (Torr. & Gray). Science 313: 1596-1604.

6. Ayele M, Haas BJ, Kumar N, Wu H, Xiao Y, et al. (2005) Whole genome shotgun sequencing of Brassica oleracea and its application to gene discovery and annotation in Arabidopsis. Genome Res 15: 487-495.
