# Supplementary material for: Diverse Lifestyles and Strategies of Plant Pathogenesis Encoded in the Genomes of Eighteen Dothideomycetes Fungi
Source: PLoS Pathog. 2012 Dec 6;8(12):e1003037. doi: 10.1371/journal.ppat.1003037 (PMC3516569; doi:10.1371/journal.ppat.1003037)
Supplement: Text S4 — Dothistromin and aflatoxin biosynthetic pathways in Dothideomycetes . (DOC) [file ppat.1003037.s042.doc]

**Dothistromin and aflatoxin biosynthetic pathways in *Dothideomycetes***

The well-described biosynthesis of dothistromin is an example of a discontinuously present secondary metabolite pathway, initially recognized in *Dothideomycetes* (Figure S5). Several *Dothideomycete* species produce dothistromin and related polyketide compounds such as versicolorins, which are chemically similar to aflatoxin biosynthetic intermediates . Genetic and biochemical studies suggest common steps in the biosynthetic pathways for dothistromin and the aflatoxin precursor versicolorin B both in terms of the chemistry and the enzymes required for their biosynthesis . Aflatoxins are amongst the most carcinogenic and toxic natural products known and are produced by many species of *Aspergillus* . Due to their high toxicity and the presence of related biosynthetic pathways in *D. septosporum*, we investigated the genetic potential of *Dothideomycetes* to produce secondary metabolites related to aflatoxins.

Reciprocal BLAST analyses with a core set of *D. septosporum* dothistromin genes suggested that only two of the other dothideomycete species, *C. fulvum* (sister species to *D. septosporum*) and *R. rufulum*, have a putative orthologous gene set (Figure S5). A search for homologs of *Aspergillus flavus* genes associated with later steps of aflatoxin biosynthesis suggested that none of the *Dothideomycetes* has the genetic potential to make aflatoxin, although this possibility cannot be ruled out on the basis of BLAST analyses alone. But strikingly the saprotroph *R. rufulum* contains orthologs of genes (*VerA, OmtB*) associated with biosynthesis of sterigmatocystin, a toxic precursor of aflatoxin. As well as being produced by many *Aspergillus* spp., sterigmatocystin production has been reported for other fungi outside the *Eurotiomycetes*, including the *Sordariomycetes* *Chaetomium* spp. (reviewed in ) and *Podospora anserina,* for which acquisition by horizontal gene transfer was proposed . Intriguingly there is an early report of sterigmatocystin production by *C. sativus* (*Bipolaris sorokiniana*) *,* one of the *Dothideomycetes* for which we found scant evidence for sterigmatocystin pathway genes. This leads to the possibility that loss or gain of these pathway genes has occurred within as well as between dothideomycete species.

Although many of the *Dothideomycetes* and other filamentous ascomycetes (*Eurotiomycetes*, *Leotiomycetes*, *Sordariomycetes*) did not appear to have orthologs of dothistromin/aflatoxin/sterigmatocystin genes based on reciprocal BLAST analyses, the abundance of non-reciprocal matches suggests the presence of paralogs for many of the genes (Figure S5). This reflects the ubiquitous occurrence within these fungi of common genes such as fatty acid synthases, cytochrome P450 monooxygenases and reductases that are commonly associated with secondary metabolite gene clusters.

1. Assante G, Locci R, Camarada L, Merlini L, Nasini G (1977) Screening of the genus *Cercospora* for secondary metabolites. Phytochemistry 16: 243-247.

2. Bradshaw RE, Jin HP, Morgan BS, Schwelm A, Teddy OR, et al. (2006) A polyketide synthase gene required for biosynthesis of the aflatoxin-like toxin, dothistromin. Mycopathologia 161: 283-294.

3. Shaw GJ, Chick M, Hodges R (1978) A ^13^C-NMR study of the biosynthesis of the anthraquinone dothistromin by *Dothistroma pini*. Phytochemistry 17: 1743-1745.

4. Henry KM, Townsend CA (2005) Ordering the reductive and cytochrome P450 oxidative steps in demethylsterigmatocystin formation yields general insights into the biosynthesis of aflatoxin and related fungal metabolites. Journal of the American Chemical Society 127: 3724-3733.

5. Schwelm A, Bradshaw RE (2010) Genetics of dothistromin biosynthesis of *Dothistroma septosporum*: an update. Toxins 2: 2680-2698.

6. Williams JH, Phillips TD, Jolly PE, Stiles JK, Jolly CM, et al. (2004) Human aflatoxicosis in developing countries: a review of toxicology, exposure, potential health consequences, and interventions. . American Journal of Clinical Nutrition 80: 1106–1122.

7. Varga J, Frisvad JC, Samson RA (2009) A reappraisal of fungi producing aflatoxins. World Mycotoxin Journal 2: 263-277.

8. Yu J, Chang P-K, Ehrlich KC, Cary JW, Bhatnagar D, et al. (2004) Clustered pathway genes in aflatoxin biosynthesis. Applied and Environmental Microbiology 70: 1253-1262.

9. Slot JC, Rokas A (2011) Horizontal transfer of a large and highly toxic secondary metabolic gene cluster between fungi. Current Biology 21: 134-139.

10. Rabie CJ, Lubben A, Steyn M (1976) Production of sterigmatocystin by *Aspergillus versicolor* and *Bipolaris sorokiniana* on semisynthetic liquid and solid media. Applied and Environmental Microbiology 32: 206-208.
